# Evolutionary and biomedical insights from a marmoset diploid genome assembly

**DOI:** 10.1038/s41586-021-03535-x

**Published:** 2021-04-28

**Authors:** Chentao Yang, Yang Zhou, Stephanie Marcus, Giulio Formenti, Lucie A. Bergeron, Zhenzhen Song, Xupeng Bi, Juraj Bergman, Marjolaine Marie C. Rousselle, Chengran Zhou, Long Zhou, Yuan Deng, Miaoquan Fang, Duo Xie, Yuanzhen Zhu, Shangjin Tan, Jacquelyn Mountcastle, Bettina Haase, Jennifer Balacco, Jonathan Wood, William Chow, Arang Rhie, Martin Pippel, Margaret M. Fabiszak, Sergey Koren, Olivier Fedrigo, Winrich A. Freiwald, Kerstin Howe, Huanming Yang, Adam M. Phillippy, Mikkel Heide Schierup, Erich D. Jarvis, Guojie Zhang

**Affiliations:** 1 BGI-Shenzhen, Shenzhen, China; 2 Villum Centre for Biodiversity Genomics, Section for Ecology and Evolution, Department of Biology, University of Copenhagen, Copenhagen, Denmark; 3 Laboratory of Neurogenetics of Language, The Rockefeller University, New York, NY USA; 4 Vertebrate Genome Laboratory, The Rockefeller University, New York, NY USA; 5 University of the Chinese Academy of Sciences, Beijing, China; 6 Bioinformatics Research Centre, Aarhus University, Aarhus, Denmark; 7 Wellcome Sanger Institute, Hinxton, UK; 8 Genome Informatics Section, Computational and Statistical Genomics Branch, National Human Genome Research Institute, National Institutes of Health, Bethesda, MD USA; 9 Max Planck Institute of Molecular Cell Biology and Genetics, Dresden, Germany; 10 Center for Systems Biology, Dresden, Germany; 11 Laboratory of Neural Systems, The Rockefeller University, New York, NY USA; 12 Center for Brains, Minds and Machines (CBMM), The Rockefeller University, New York, NY USA; 13 James D. Watson Institute of Genome Sciences, Hangzhou, China; 14 Guangdong Provincial Academician Workstation of BGI Synthetic Genomics, BGI-Shenzhen, Shenzhen, China; 15 Howard Hughes Medical Institute, Chevy Chase, MD USA; 16 State Key Laboratory of Genetic Resources and Evolution, Kunming Institute of Zoology, Chinese Academy of Sciences, Kunming, China; 17 China National GeneBank, BGI-Shenzhen, Shenzhen, China; 18 Center for Excellence in Animal Evolution and Genetics, Chinese Academy of Sciences, Kunming, China

**Keywords:** Genome informatics, Evolutionary genetics, Genomics, Genetics of the nervous system, Experimental models of disease

## Abstract

The accurate and complete assembly of both haplotype sequences of a diploid organism is essential to understanding the role of variation in genome functions, phenotypes and diseases^1^. Here, using a trio-binning approach, we present a high-quality, diploid reference genome, with both haplotypes assembled independently at the chromosome level, for the common marmoset (*Callithrix jacchus*), an primate model system that is widely used in biomedical research^2,3^. The full spectrum of heterozygosity between the two haplotypes involves 1.36% of the genome—much higher than the 0.13% indicated by the standard estimation based on single-nucleotide heterozygosity alone. The de novo mutation rate is 0.43 × 10^−8^ per site per generation, and the paternal inherited genome acquired twice as many mutations as the maternal. Our diploid assembly enabled us to discover a recent expansion of the sex-differentiation region and unique evolutionary changes in the marmoset Y chromosome. In addition, we identified many genes with signatures of positive selection that might have contributed to the evolution of *Callithrix* biological features. Brain-related genes were highly conserved between marmosets and humans, although several genes experienced lineage-specific copy number variations or diversifying selection, with implications for the use of marmosets as a model system.

## Abstract (web summary)

A trio-binning approach is used to produce a fully haplotype-resolved diploid genome assembly for the common marmoset, providing insight into the heterozygosity spectrum and the evolution of the sex-differentiation region.

## Main

A diploid organism carries two haploid genomes with a range of variants, which make substantial contributions to phenotypic variation^4^. Phased haplotype assemblies can help to reveal the *cis*- and *trans*-acting variants on the two homologous genomes. However, most contemporary de novo genome-sequencing efforts produce a single mosaic reference genome derived from parts of both maternal and paternal alleles, with variations between homologous chromosomes normally being disregarded. As a consequence, these methods usually fail to assemble genomic regions with high heterogeneity, resulting in fragmented sequences. A few methods have been developed to produce partial haplotype-phased genome assemblies and showed power in using long sequencing reads to produce long haplotigs (haplotype-specific contigs)^5,6^. However, producing an assembly that is completely phased at the chromosome level for both haplotypes of a diploid genome remains a challenge. Here, as part of the Vertebrate Genomes Project, we used a trio-binning approach^7,8^ to produce a chromosome-level, fully haplotype-resolved diploid genome assembly for the common marmoset, *C. jacchus*. This New World primate has been established as an animal model for a broad range of biomedical research such as neuroscience, stem cell biology and regenerative medicine^2,3^. With our high-quality diploid assembly, we discovered new properties of heterozygosity on both autosomes and sex chromosomes of this primate species.

## Diploid genome assembly

**Extended Data Fig. 1:**
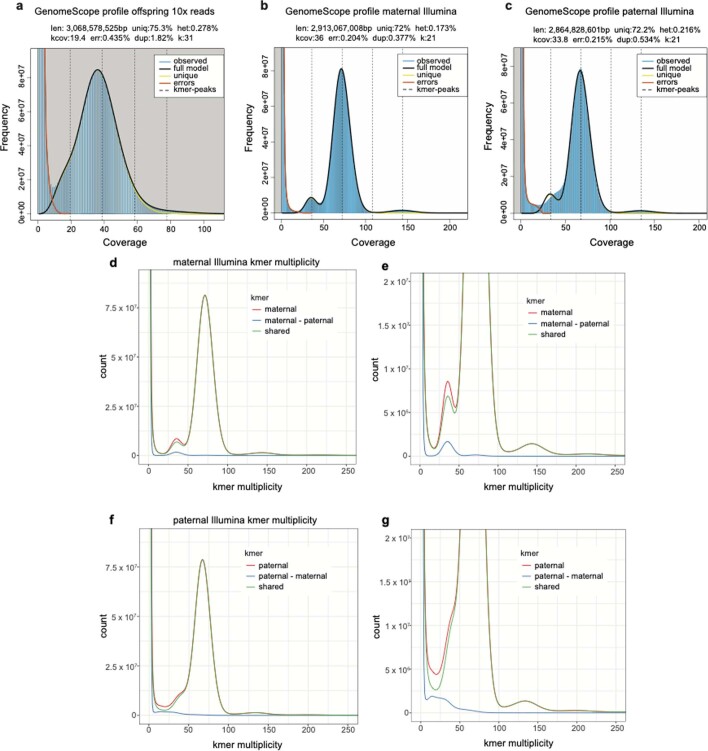
GenomeScope analyses. **a**, GenomeScope (v.1.0) profile for 31-mers collected from the F_1_ 10X linked-reads using Meryl (https://github.com/marbl/meryl) (following GEM (gel-bead in emulsion) barcode trimming). Heterozygosity estimated at a maximum of 0.287%. Read error rate estimated at a maximum of 0.435%. Genome haploid length estimated at a maximum of 3,068,578,525 bp, repeat length estimated at a maximum of 757,852,942 bp and unique length estimated at a maximum of 2,310,725,582 bp. **b**, **c**, Genomescope profiles of the maternal (**b**) and paternal (**c**) 21-mers collected from the raw Illumina data. The observed paternal data do not fit GenomeScope’s robust model (black line) for a diploid organism and exhibit higher overall heterozygosity than the maternal data (0.216% compared to 0.173%). This supports a premise that the father’s sequencing reads contain a level of chimerism, whereas the mother’s reads contain negligible representation of alternative alleles, at most. Further analysis of the parental Illumina data shows that the *k*-mer multiplicity distribution varies greatly between the maternal and paternal sets. **d**–**g**, The maternal *k*-mers (**d**, **e** (**e** shows a magnified version of **d**)) show clear distributions with a distinct haploid peak at half coverage (around 35×), whereas the paternal *k*-mers (**f**, **g** (**g** shows a magnified version of **f**)) show an irregular distribution with no clearly defined haploid peak. This provides further evidence that the paternal data exhibit a level of chimerism.

**Extended Data Fig. 2:**
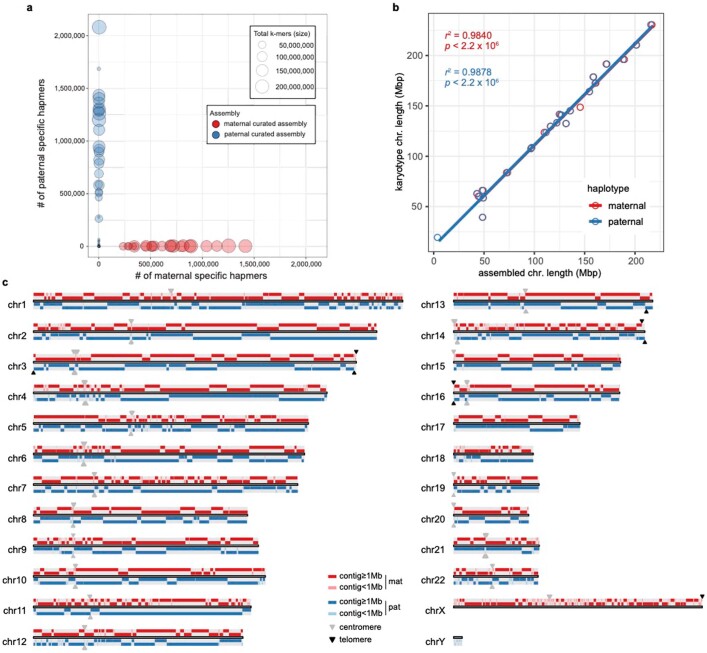
Trio-based diploid genome assembly. **a**, Hapmer (haplotype-specific *k*-mer) blob plot of the curated marmoset assemblies. Red, maternal haplotype; blue, paternal haplotype. The size of each blob indicates the total number of *k*-mers counted in an individual scaffold and the position of each blob is plotted according to the number of contained maternal and paternal hapmers. We see that maternal and paternal hapmers are highly phased, with some slight representation of paternal hapmers in several maternal scaffolds (those that do not lie directly on the *x* axis). We can also see a higher representation of paternal hapmers identified within scaffolds of the paternal assembly than maternal hapmers identified in scaffolds of the maternal assembly. **b**, Correlation between the assembled chromosome sizes and the chromosome lengths estimated by karyotype image data. A total of 23 chromosomes are plotted and the coefficient of determination is calculated for each assembly. **c**, Schematic plot mapping the assembled maternal and paternal assigned contigs onto marmoset assembled chromosomes. Top, maternal alleles; bottom, paternal alleles. Contig sizes, centromeres and telomeres are indicated.

We generated 63×-coverage PacBio continuous long reads, 55× 10X Genomics Chromium linked-reads, 154× Bionano optical molecules, 105× chromosome conformation capture (Hi-C) reads from a captive male F_1_ marmoset and 70× short-read sequences from the DNA of both parents (Supplementary Table 1, Supplementary Fig. 1). We used an updated version of TrioCanu^7,8^ to bin the PacBio long reads of the F_1_ marmoset via *k*-mers of the parental short reads, and assembled each set into haploid-specific contigs, which were independently scaffolded with the 10X, Bionano and Hi-C data^8^ (Extended Data Fig. 1, Supplementary Fig. 2, Supplementary Tables 2, 3). The final contig and scaffold NG50 values after manually curation were 7.7. Mb and 146 Mb for the maternal assembly and 12.1 Mb and 136 Mb for the paternal assembly, respectively. *k*-mer assessment indicated that the assemblies were fully phased (Extended Data Fig. 2a, Supplementary Figs. 3, 4). Each haploid genome includes 22 autosomes and each of the two sex chromosomes (X and Y), with 99.45% and 98.94% of the maternal and paternal alleles assigned to chromosomes, respectively. The assembled chromosome lengths showed a clear linear correlation with the estimated marmoset karyotype lengths^8,9^ (Extended Data Fig. 2b, Supplementary Note, Supplementary Tables 4, 5, Supplementary Fig. 5). Although marmosets show prevalent genetic chimerism between twins and triplets in utero^10^, the chimeric level of the F_1_ male muscle sample used in this study was very low, as expected^11^ (Extended Data Fig. 1d–g, Supplementary Fig. 6, Supplementary Tables 6, 7, Supplementary Note).

We estimated the single-base-pair accuracy rate to be 99.996% for the maternal assembly and 99.998% for the paternal assembly (Supplementary Note, Supplementary Fig. 7, Supplementary Tables 8, 9). About 93% and 88% of the gaps in the previously published marmoset reference genome cj3.2^12^ were closed in our maternal and paternal assemblies, respectively, and both showed an increase of over 290-fold in contig N50, with 95.75% and 93.62% of the contigs being over 1 Mb, respectively (Extended Data Fig. 2c). Iso-Seq full-length transcriptome data also suggest a high completeness of our assembly (Supplementary Note, Supplementary Tables 10, 11). Comparison with two other recently released chromosome-level assemblies (cj1700 and cj2019) showed 16 large intra-chromosome-level structural variants (SVs) (larger than 1 Mb) and 3 inter-chromosomal SVs (Supplementary Tables 12, 13). PacBio long reads and 10X linked-reads confirmed that our assemblies were correct (Supplementary Figs. 8, 9, Supplementary Tables 12–14). However, these differences may also be due to the large structural polymorphisms.

## Heterozygosity between parental genomes

**Fig. 1:**
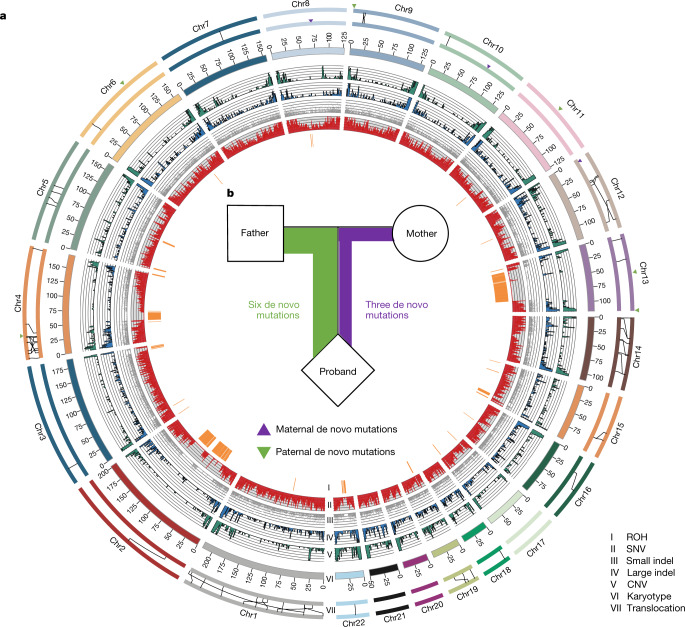
Distribution of SNVs, small indels and SVs in a diploid marmoset genome. **a**, Heterozygosity landscape patterns between the two haploid marmoset genomes. Tracks from inside out (I–VI): distribution of runs of homozygosity (ROH) (>1 Mb), SNV density (window size, 500 kb; range, 0–0.85%), small indel (<50 bp) distribution (*y* axis, indel length), large indel density (≥50 bp; window size, 1 Mb; count, 0–9), CNV density (window size, 1 Mb; count, 0–9) and karyotype. The links in the outermost circles denote differences in translocation events between maternal (inner) and paternal (outer) assemblies (VII). Triangles indicate locations of the de novo mutations in parental alleles. **b**, Schematic showing the proportion of parental sources of the de novo mutations.

**Fig. 2:**
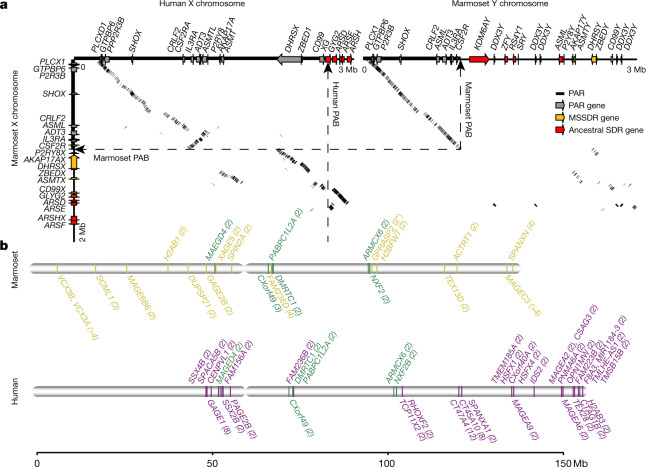
Structures of sex chromosomes in marmosets and humans. **a**, Alignment between the marmoset X and Y chromosome reveals a PAR of around 1 Mb in each chromosome. Dashed lines show the boundaries between the PAR and SDR. Alignment between the human and marmoset X chromosome also reveals different PABs between the two species, and an inversion near the marmoset PAB. Grey, PAR genes; orange, MSSDR genes; red, ancestral SDR genes. *ARSE* is also known as *ARSL*. **b**, Distribution of ampliconic genes in the marmoset (yellow) and human (purple) X chromosome. Green, genes that are ampliconic in both species. The copy number for each ampliconic gene is shown in parentheses. Asterisks indicate partial genes. Ampliconic genes with testis-specific expression are shown as the bottom half of the panel for each species. *IDS2* is also known as *IDSP1*.

In traditional genome-sequencing efforts, heterozygosity is normally estimated by mapping sequencing reads onto a mosaic reference genome, resulting in limited phase information of the heterozygous variants. Our assemblies enable us to directly compare the two parentally inherited genomes and identify the full spectrum of genetic variants between the parental alleles, including single nucleotide variations (SNVs), insertion and deletions (indels) and large SVs (Supplementary Fig. 10). We identified 3.47 million SNVs and around 232,000 short (maximum of 50 base pairs (bp)) indels across the whole genome (Fig. 1a), with 96.5% SNVs confirmed by short-read mapping. PCR experiments validated 99.6% and 95.2% randomly selected SNVs and short indels (Supplementary Note, Supplementary Tables 15–17), indicating that our diploid assembly enabled us to detect allelic variants with considerably high accuracy. We found a correlation between SNV rate and indel rate (Supplementary Fig. 11a), in which both displayed a unimodal distribution across the genomes (Supplementary Figs. 11b, 12). Consistent with laboratory inbreeding, we observed 28 genomic regions with long runs of homozygosity (Fig. 2a), with the longest one spanning more than 10 Mb (Supplementary Fig. 13a). This pattern can also be detected in other marmoset samples with short-read resequencing data^13^ (Supplementary Fig. 13b, Supplementary Table 18), suggesting that captive marmosets are suffering a notable reduction of genetic diversity.

Heterozygous variation in regulatory or coding regions could result in allele-specific expression profiles or different products of the same genes from the two alleles^14^. We found that approximately 1.1% of SNVs and 0.58% of indels were located in protein-coding genes or regulatory regions. In particular, 8,144 SNVs caused non-synonymous substitutions and 274 indels caused frame-shifting mutations, which can produce allele-specific transcripts and proteins. This observation was validated by the Iso-Seq data, in which we detected that 2,537 genes produced transcripts with variation in open-reading frames from the parental alleles (Supplementary Fig. 14).

SVs contribute substantial genetic diversity with important evolutionary and medical implications. By comparing the two haploid genomes, we identified 11,663 SVs (larger than 50 bp), including 6,064 large indels, 27 inversions, 34 translocations, 5,514 copy number variations (CNVs) and 24 inverted translocations (Fig. 2a, Supplementary Table 19). We validated 95.7% of the large indels and 74.2% of the SVs with PacBio long reads, as well as 14 of 17 randomly selected large indels by PCR (Supplementary Fig. 15, Supplementary Table 20). By counting all types of variation between the two haploid genomes, we estimate the overall rate of heterozygosity on the autosomes of the sequenced individual to be around 1.36%.

Large heterogeneous SVs could cause a high incidence of chromosomally unbalanced gametes and thus are normally rare^15^. We found that 72% of SVs were shorter than 1.5 kb, with an average length of about 3.5 kb. The longest SV was a 304-kb inversion (Supplementary Fig. 16). We observed a higher density of LINE (L1) elements around the inversions (*P* = 0.03752, one-sided *t*-test). The indel peak at a length of 300 bp were enriched with Alu repeats (Supplementary Fig. 17a; *P* = 2.2 × 10^−16^, Chi-squared test, Supplementary Note). About 33% of the inversion variations between haplotypes were located between two inverted repeat sequences (Supplementary Fig. 17b), indicating that they were introduced by a repeat mechanism^16^. We detected and validated 58 genomic translocation events that differed between the two haplotypes, including 50 genes (Fig. 2a, Supplementary Table 21). About half of the affected genes were completely translocated from one allele to a different genomic location in the other allele. The mechanism driving such translocations remains to be elucidated.

## De novo germline mutations

Germline mutations are the source of genetic diversity and the driving force of both evolution and genetic diseases^17^. However, finding de novo germline mutations is a challenging task, as in traditional assemblies less than half of the mutations can be phased to parental origin^18^. A fully diploid assembly enables us to use each parental haplotype independently as a reference to detect de novo mutations, and validate the loci detected independently from the two references as controls for false-positive calls (Methods, Supplementary Note). We detected nine validated de novo mutations in this trio from the approximately 41% of callable sites in both maternal and paternal genomes (Fig. 1a, Supplementary Table 22). The paternal-to-maternal ratio contribution of de novo mutations to the child was 2:1 (Fig. 1b), which is lower than that in humans (4:1)^18^ but similar to the closely related owl monkey (2.1:1)^19^. Our results suggest a mutation rate of 0.43 × 10^−8^ de novo mutations per site per generation for the marmoset. Using this estimated rate and the evolutionary branch length of marmoset substitutions inferred from whole-genome alignments^20^, we estimated a divergence time between New World monkeys and humans at around 48.7 million years ago (Ma), which is close to what was estimated from data for the owl monkey^19^.

## New sex-differentiation region in the marmoset

On the basis of the sequencing depth of parental short reads on the F_1_ male assembly (Methods), we identified X-linked sequences of around 147 Mb, with over 99% in a single X chromosome scaffold (Supplementary Table 23). As the Y chromosome is enriched with repeat elements and segmental duplications, we de-collapsed unplaced and potential Y-linked scaffolds^21^ (Supplementary Fig. 18a) then combined read-depth information and Hi-C interactions to identify final Y-linked sequences of 13.85 Mb (Supplementary Fig. 18b, Supplementary Table 24, Methods). This is smaller yet closer to the 20-Mb karyotype estimate^9^ and longer than that in other assemblies (Supplementary Table 25).

**Extended Data Fig. 3:**
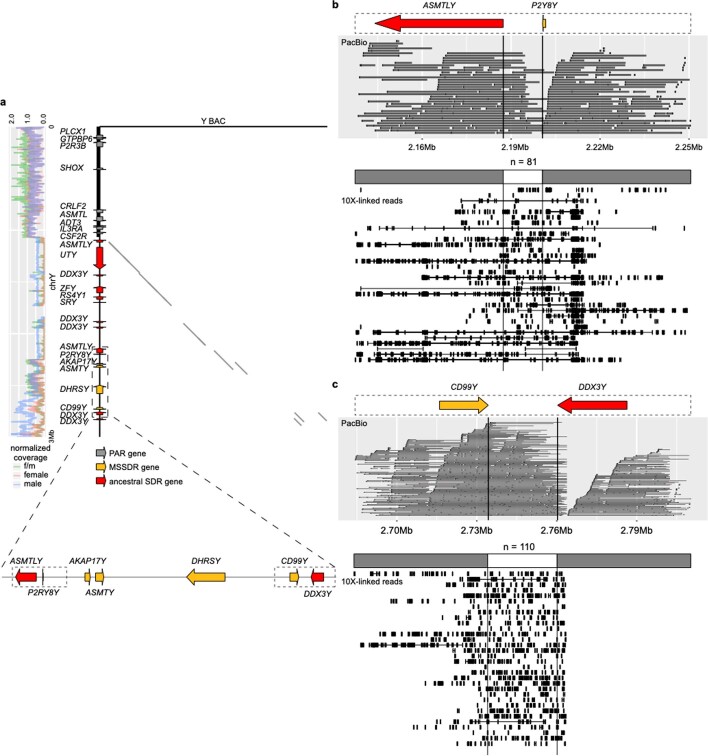
Confirmation of the MSSDR translocation in the marmoset Y chromosome. **a**, Marmoset Y-chromosome-specific BAC reads were obtained from the NCBI trace archive and constructed into a pseudo-Y chromosome according to their position from a previous study^20^. The linear alignment between mCalJac1’s Y chromosome and marmoset bacterial artificial chromosome mapped to the Y chromosome confirms the MSSDR translocation. The MSSDR translocation on the Y chromosome is highlighted in yellow and the two regions that span the break points and its flanking 50 kb are highlighted in dashed boxes. **b**, The region spanning *ASMTLY* and *P2RY8Y* is supported by PacBio reads and 10X linked-reads (only a proportion of them were shown). In the 10X linked-reads panel, each rectangle represents a read and each line represents a 10X DNA molecule. A total of 81 10X linked-read DNA molecules support the linkage of *ASMTLY* and *P2RY8Y*. **c**, The region spanning *CD99Y* and *DDX3Y* is supported by PacBio reads and 10X linked-reads (only a proportion of them shown). A total of 110 10X linked-read DNA molecules support the linkage of *CD99Y* and *DDX3Y*.

Our diploid assembly resolved pseudoautosomal regions (PARs) of both the X and the Y chromosome, whereas most other male genomes result in collapsing PARs into one copy with mixed origin. This permits the precise identification of the pseudoautosomal boundary (PAB) in marmosets (Fig. 2a). Marmoset PARs contain nine protein-coding genes, all of which are also found in the human PAR. However, an inversion was found between human and marmoset PARs, and it is likely to occur specifically in the marmoset lineage near its PAB (Fig. 2a, Supplementary Fig. 19). In addition, downstream of this inversion in the X chromosome, we observed a genomic sequence spanning six human PAR orthologues that had become a new sex-differentiation region (SDR) in the marmoset (Fig. 2a). Three genes in the region, *P2RY8Y*, *AKAP17AY* and *ZBEDY*, have been reported to be SDR-linked^22^. We found that they were not collinear with the X chromosome, but were translocated to the middle of the Y chromosome (Fig. 2a, Extended Data Fig. 3, Supplementary Table 26). All of the Y copies accumulated more mutations than their corresponding X copies (Supplementary Fig. 20). Their X–Y genetic divergence was significantly higher than that of the PAR (one-sided *t*-test, *t* = 5.7694, *P* = 1.468 × 10^−6^) (Supplementary Table 27), but significantly lower than that of the ancestral SDR (one-sided *t*-test, *t* = −8.9434, *P* = 3.319 × 10^−13^) (Supplementary Fig. 21), suggesting that its recombination suppression began recently. These new SDR genes also showed a bias in expression in females; however, they were not significantly different from PAR or ancestral SDR genes (Supplementary Fig. 22).

We next applied two divergence-based methods to date the formation of the marmoset-specific SDR (MSSDR) (Supplementary Note, Supplementary Tables 28, 29). On the basis of the marmoset mutation rate estimated above, we inferred that the MSSDR formed at 5.23–9.41 Ma (Supplementary Tables 30, 31). Applying lower mutation rates of the closely related African green monkey (1.11 × 10^−9^ mutations per position per year (PPPY))^23^ and the owl monkey (1.20 × 10^−9^ PPPY)^24^, the formation of the MSSDR was dated at 6.67–12.97 Ma. All of these results indicate that the expansion of the SDR in the marmoset is an evolutionarily young event.

The translocation of the MSSDR on the Y chromosome makes the PAR of the marmoset the shortest among primates recorded so far^25^. As X–Y recombination during male meiosis is limited to the PAR, this region is known to contain the highest per-site recombination rate in the genome^26^ and an increased intensity of GC-biased gene conversion^27^. Consistently, we observed a higher GC content in the marmoset PAR relative to the human PAR (one-sided *t*-test, *t* = 3.1327, *P* = 0.0011) (Supplementary Fig. 21). We also observed a 4.3-fold-higher rate of heterozygosity in the marmoset PAR (0.52%) compared to the average rate in autosomes (0.12%) (Supplementary Fig. 23), suggesting that more-intense recombination in the shorter marmoset PAR causes more mutations.

Ampliconic genes—genes with highly similar adjacent copies—are a notable and enigmatic feature of most sex chromosomes^28^. They are often found specifically expressed in the testes and experience a very rapid turnover of copy number^29^, leading to the hypothesis that ampliconic genes are involved in sexual antagonism^29^. We detected 22 ampliconic genes on the marmoset X chromosome (Fig. 2b), of which 12 showed testes-restricted expression, at a proportion close to that in humans (40%). Six of the marmoset X-linked ampliconic genes were also present in the human X chromosome with overall similar duplication patterns, suggesting that they originated from a common ancestor (Fig. 2b, Supplementary Fig. 24). The marmoset Y chromosome also contains five multi-copy genes, of which two (*TSPY* and *RBMY*) are also ampliconic genes in the human Y chromosome^30^. These results suggest that the sex-linked ampliconic genes have evolved under a very dynamic duplication process during primate evolution.

## Rapid evolution of the marmoset Y chromosome

**Fig. 3:**
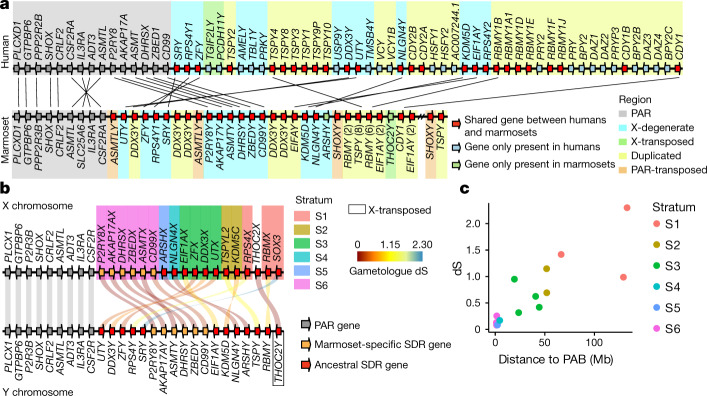
Comparison of sex chromosomes across species. **a**, Y-chromosome gene synteny between humans and marmosets. Lines between human and marmoset indicate one-to-one orthologues. Distance is not drawn to scale. The number of paralogues in unplaced de-collapsed Y-linked scaffolds are marked in parentheses. *ADT3* is also known as *SLC25A6*; *AC007244.1* is ENSG00000286265 under Ensembl release 98. **b**, Six evolutionary strata found in marmoset sex chromosomes. The colour of the links between X and Y gametologues indicates the pairwise dS value. *THOC2X-Y* was not included in any strata because it is a very recently emerged gametologue pair formed via duplication. **c**, Correlation between pairwise dS and X-chromosome position for 14 X–Y SDR gametologues outside the marmoset PAR. Each point represents one gametologue.

In contrast to the X chromosome, which maintained overall conserved synteny during primate evolution (Supplementary Fig. 25), we found that the Y chromosome experienced rapid structural changes. This is probably due to the accumulation of mutations as a consequence of Muller’s ratchet effect^31^. We detected at least three large inversions and one large translocation involving genes between the male-specific region of the Y chromosome (MSY) in humans and marmosets. The human MSY contained 48 protein-coding genes and the marmoset MSY contained 46, but with different gene properties (Fig. 3a): Twenty-two human MSY genes were absent in the marmoset; of these, 15 of evolved during the evolution of the Hominoidea and the rest were ancestral gametologues that have become inactive or been lost in marmosets (Fig. 3a). Several MSY genes crucial for spermatogenic functions (for example, *HSFY1* and *VCY*) (Supplementary Note) have been lost in marmosets, or lost function owing to frame-shift mutations (for example, *USP9Y*) (Supplementary Fig. 26). The loss of these genes might be associated with the monogamous social structure of marmosets^32^, which potentially alleviates sperm competition. These findings indicate that although it has been claimed that the marmoset has similar patterns of spermatogenesis to humans^33^, there are probably some key differences associated with these genes.

By contrast, the marmoset MSY only contains two genes that are absent in humans—*ARSHY* and *THOC2Y*. *THOC2Y* was thought to be lost early in the eutherian common ancestor and exhibits a high rate of synonymous substitutions (dS value) with its gametologue in marsupials^34^. However, we found that the marmoset *THOC2Y* has a very low dS value (dS = 0.0502) with its X-linked gametologue, suggesting that it is not the ancestral gene but a marmoset-specific MSY gene that has recently been duplicated from its X-chromosome counterpart (Supplementary Fig. 27a). In humans, *THOC2* is widely expressed in many tissues and interacts with *XPO4*^35^ which mediates the import of SOX2 and SRY proteins. In the marmoset, both *THOC2X* and *THOC2Y* have become testis-specific genes (tissue specificity index (Tau) > 0.8) (Supplementary Fig. 27b). The remaining MSY genes are present in both species, but some show CNVs (Fig. 3a, Supplementary Fig. 28).

Of the 46 marmoset MSY genes, 18 have their gametologues on the X chromosome (Fig. 3b), and their pairwise dS values between X and Y increased with their distance to the PAB on the X chromosome (Pearson’s *r* = 0.8342, *P* = 0.0002) (Fig. 3c, Supplementary Table 27), as in humans^36^. According to the sequence divergence as well as the phylogeny, we inferred the presence of six evolutionary strata in marmoset sex chromosomes, which we named from the oldest to the youngest, S1 to S6 (Fig. 3b). S1–S4 are shared with humans^22,36^ (Supplementary Fig. 29), suggesting an ancient origin. S5 of the marmoset contained one gametologue pair, *ARSHX-Y*, which has a low pairwise dS value (0.0605) close to that of gametologues in the MSSDR (Supplementary Table 27). In addition, the X copy of the marmoset is clustered with its Y copy instead of the X copies of other primates (Supplementary Fig. 30), suggesting that this stratum formed specifically in New World monkeys. S6 contained six pairs of gametologues, all residing in the MSSDR. The pairwise dS values of S6 gametologues are much lower than those of the ancestral gametologues (Fig. 3b). Notably, three gametologues (*DHRSX-Y*, *ASMTX-Y* and *CD99X-Y*) in S6 display the highest ratio of pairwise non-synonymous to synonymous substitutions rates (dN/dS value) among all gametologues (Supplementary Table 27). Of them, *CD99X* and *CD99Y* show tissue-specific expression in ovary and testis, respectively (Supplementary Table 32). These features imply a strong directional selection link to sex differentiation on these genes once they were translocated from the PAR in the marmoset.

## Genetic basis of marmoset biological traits

As a representative species of Callitrichidae, the marmoset has many notable biological traits, such as small body size^37^, twinning^12,38^, exudate feeding^39^ and maintaining bone density during ageing owing to reduced levels of gonadal oestrogen (thus marmosets do not suffer from age-related osteoporosis^40,41^). To further expand our knowledge on the evolution of these biological features, we scanned for and identified 204 positively selected genes (PSGs) in the marmoset genome and 38 PSGs in the common ancestor of New World monkeys (Supplementary Tables 33–35). We have manually checked these PSGs to avoid potential artefacts due to alignment errors or the differences in sequencing and annotation methods across genomes, although we cannot fully rule out the possibility that the differences in quality between the compared assemblies could have affected some of these results. Among these genes, we found two that may be linked to manifesting diminutive size. Mutations of *ZDHHC13* (PSG in marmosets) in mice causes post-translational lipid modification, resulting in weight loss and reduced bone mineral density^42^. *FGFR1* (PSG in New World monkeys) regulates a feedback signal to control the rate of differentiation of osteoblasts^43^, and mutations cause autosomal dominant skeletal disorder^44^. (Supplementary Fig. 31).

Marmosets exhibit several unique reproductive adaptations^37^, which include sharing a common placental circulation with siblings^45^ and the suppression of reproduction in nondominant females^46^. Previous studies have identified several candidate genes that might be related to these traits^12,38^. We found three marmoset PSGs (*PCSK6*, *NR1D1* and *TGIF1*) that might also contribute to their reproductive adaptation. *PCSK6* is expressed in numerous ovarian cell types and *PCSK6*-mutant mice exhibit progressive loss of ovarian function and formation of ovarian pathology^47^. *NR1D1* is a circadian clock gene and might interact with the gonadotropin-releasing hormone signalling pathway^48^. Knockout of this gene in mice reduces fertility^49^. *TGIF1* is a repressor and reversibly modulates members of the TGF‐β/SMAD signalling pathway, which has an important role in reproductive processes, including follicular activation, ovarian follicle development and oocyte maturation^50^.

We found three marmoset PSGs (*BCL2L14*, *HOMER3* and *CHADL*) involved in osteoclastogenesis and bone metabolism. *BCL2L14* encodes a member of an anti-apoptotic family of proteins, which are known to suppress the functions of osteoclasts^51^. *HOMER3* participates in osteoclastogenesis and bone metabolism. Deletion of this gene markedly decreased tibia bone density, resulting in bone erosion in mice^52^. *CHADL* encodes a collagen-associated small leucine-rich protein and may influence the differentiation of chondrocytes by acting on its cellular microenvironment^53^. Further experiments are needed to investigate the potential roles of the positively selected substitutions in specialized bone metabolism in marmosets.

Captive marmosets in laboratories are intermittently plagued by gastrointestinal disorders^54^, which may result from dietary differences in captivity versus the wild^55^. Wild marmosets feed on gums as one of their primary food sources, to acquire energy and minerals^39^. Compared to captive marmosets, the gut microbiome of wild marmosets is more enriched with *Bifidobacterium*^56^. This probiotic bacterium may function to assist the digestion of gum^57^. We found that *PTGS1*, which mediates the gastrointestinal inflammatory reaction, was under positive selection in the marmoset. Expression of this gene is higher in the intestinal mucosa of obese rats than rats of a normal weight^58,59^, but its expression is reduced to normal levels when rats are fed with *Bifidobacterium*^59^. It seems that *PTGS1* may have a role in the gastrointestinal function of marmosets, which might be regulated by their exudivore diet through the probiotic bacteria.

## Genomic insights for biomedical research

Marmosets are becoming widely used as primate biomedical models in the neurosciences^2^. Here, we compared 2,533 genes related to brain development and neurodegenerative diseases, and found that the majority are highly conserved between marmosets and humans in both sequence and copy numbers (Supplementary Fig. 32). However, we detected 24 genes that show CNVs and 8 genes that are under diversification selection between the two species. These may be associated with differences in the brain between humans and marmosets (Supplementary Fig. 33, Supplementary Tables 36, 37, Supplementary Note).

Pathogenic effects of mutations are highly dependent on their genomic context^60,61^. We therefore scanned the marmoset genome for human pathogenic sites that cause or increase the risk of nervous system diseases. Notably, four genes in marmosets include substitutions that encode amino acids that are pathogenic in humans: *APOE*^*C130R*^, *GBA*^*N227S*^, *SNCA*^*A53T*^ and *PAH*^*R176Q*^ (Supplementary Figs. 34–36, Supplementary Table 38). All of them are fixed in the 12 marmoset individuals with genomic data^13^. Comparison with other primates suggests that the *GBA* and *PAH* genomic contexts are unique to the marmoset (Supplementary Figs. 35, 36). The presence of these two marmoset genes encoding amino acids that are pathogenic in humans suggests that this species might have evolved specific mechanisms to compensate for their pathogenic effects, and highlights the critical need to consider variation in the genomic context when using marmosets as models in human disease research.

## Benefits of a diploid assembly

The ultimate goal of creating a reference genome assembly is to produce a gapless, chromosome-level assembly with all sequences fully phased into haplotypes. Several previous efforts have been made towards this goal using the information of a pedigree and/or long reads^5,6^. Our findings demonstrate the power of using a trio-binning approach, in combination with long-read sequencing^7,8^, to produce a diploid genome with the two parental haplotypes assembled independently. This method captures the full range of heterozygous variations at high rates of accuracy between the two alleles, resulting in a rate of heterozygosity that is 10 times higher than that found in most genomic studies that use only heterozygous SNVs. Our diploid assembly includes sequences that are more complete for both sex chromosomes—a particular challenge in the case of the Y chromosome with its densely repetitive elements. Whenever trio samples are available, this sequencing and assembly strategy offers the means to generate high-quality, phased reference genomes for a range of species, especially those with high rates of heterozygosity.

## Methods

### Sample collection, processing and sequencing

Samples were collected at an AAALAC-accredited facility from an F_1_ male marmoset (3 months old) at The Rockefeller University, under USDA- and IACUC-approved protocols. The quadriceps muscle was dissected, collected and flash-frozen in liquid nitrogen immediately after euthasol administration; we extracted genomic DNA from the muscle sample. This DNA was used for Bionano optical mapping, PacBio library preparation and SMRT sequencing, 10X Genomics linked-read sequencing, Arima Hi-C library preparation and Illumina sequencing. We collected blood samples from both parents of the F_1_ male (mother, 3 years 10 months; father, 3 years 7 months) for Illumina sequencing by shaving the area (thigh for saphenous vein and tail for lateral tail vein), applying 2% lidocaine jelly, prepping the vein with alcohol and collecting less than 2 ml blood per sample (1× sample for male and female) via intravenous blood draw into EDTA tubes.

For annotation purposes, we collected more than 18 tissues from the brother of the F_1_ male. Blood was collected from the saphenous vein pre-mortem using the method described above. All additional tissues were dissected, collected and flash-frozen in liquid nitrogen or powdered dry ice immediately after euthasol administration; the brain and testes were dissected at first and all tissues were dissected and frozen within a 30-min period post-mortem. RNA integrity numbers (RINs) for all tissues used for PacBio SMRT sequencing and Iso-Seq analysis (‘Sample processing and sequencing’ in Supplementary Note) were high, ranging from 8.2 (lung) to 9.9 (cerebellum). We performed Mashmap quality control analyses of sequencing reads to rule out any potential contamination or poor sequencing before assembling (Supplementary Fig. 1).

### Sample size, randomization and blinding

We aim to use parental SNVs to determine and phase the two haplotype genomes of the offspring, thus the sample size for genome sequencing is three. Bioinformatic analyses were performed with all available data. Randomization for genome and transcriptome sequencing is not applied in this study. For SNV and indel PCR validation, variation sites were randomly selected by the Linux command ‘sort –R’. Blinding was not necessary for genome and transcriptome sequencing or PCR validation of genetic variation. The study aims to identify the genetic differences inherited from parental genomes, so only the DNA sample of the F_1_ individual was used for PCR validation.

### Genome assembly

We combined the previously developed trio-binning approach^7^ and further advanced the Vertebrate Genomes Project (VGP) assembly pipeline^8^ for scaffolding, to generate the haplotype-phased marmoset assembly (Supplementary Fig. 2). In the first step, we used TrioCanu (v.1.8+287) to bin PacBio long reads of the F_1_ male into maternal and paternal haplotypes using haplotype-specific 21-mer markers generated from the Illumina short reads of the mother and father. After binning, TrioCanu independently generated contigs for each haplotype (haplotigs). From here on, the maternal and paternal haplotigs underwent the same steps independently. Separately, we assembled the mitochondrial genome with the mitoVGP pipeline (v.2.2)^62^ and added it to the haplotigs to keep any raw mitochondrial reads from being mapped to nuclear sequences, which would result in lower sequence quality after polishing. We used Arrow from SMRT Link (v.6.0.0.47841) to improve base-calling accuracy and purge_dups (v.1.0.0)^63^ in an adapted trio mode to remove overlaps at the ends of contigs. The resulting polished, purged haplotigs were scaffolded in three stages: first, we used the 10X linked-reads in two rounds of Scaff10X (v.4.1.0) (https://github.com/wtsi-hpag/Scaff10X) to generate the primary scaffolds; second, we generated Bionano cmaps and used Bionano Solve (v.3.2.1_04122018)^64^ for hybrid scaffolding and to break mis-assemblies; third, we used Salsa2 (v2.2)^65^ to generate chromosome-level scaffolds using the molecular contact information from Hi-C linked reads. Finally, we performed a second round of Arrow polishing on the maternal and paternal scaffolds with the binned long reads. During this round of polishing, gaps between contigs were closed by the gap-filling function of Arrow. The parental haplotypes were then combined in a single assembly and underwent two rounds of short-read polishing using Long Ranger (v.2.2.2)^66^ for short-read alignment and freebayes (v.1.3.1)^67^ for polishing (Supplementary Note). After splitting the scaffolds by haplotype and removing the mitochondrial genome from each assembly, the two assemblies (named mCalJac1.mat and mCalJac1.pat) underwent manual curation using the gEVAL tool^68^, in particular to correct structural assembly errors. In the abbreviated name, m is mammal; CalJac is the abbreviated Latin species name; 1 is the first VGP assembly of this species; and mat and pat are maternal and paternal haplotypes, respectively.

### Identification of sex-linked sequences and additional Y-chromosome assembly

To identify X-linked and Y-linked sequences in mCalJac1 (GCA_011100555.1), we mapped parental short reads to the assembly with BWA ALN (v.0.7.12)^69^. Coverage was extracted with SAMTools (v.1.2) and normalized by the peak coverage. In the identification of X-linked sequences, the normalized female-versus-male (F/M) coverage ratio was calculated and plotted in a 5-kb window, and scaffolds with a F/M coverage ratio within the range 1.5 to 2.5 were identified as X-linked. In Y-linked sequence identification, the normalized F/M coverage ratio was calculated and plotted in a 2-kb window and scaffolds with a F/M coverage ratio within a 0.0 to 0.3 range were identified as Y-linked. We further manually examined large scaffolds in the maternal and paternal assemblies and included the Y chromosome Super_scaffold_pat_24. This scaffold was missing in the 0.3 cut-off condition because the first 1-Mb sequence shows an equal pattern of female and male coverage as the PAR.

In these previous steps, only Y-linked sequences of around 6 Mb were identified, about 14 Mb smaller than the expected 20-Mb size based on karyotyping. As sex chromosomes are notoriously difficult to assembly, and no primate has had a complete Y chromosome sequenced, to determine whether we missed any Y-chromosomal sequences, we performed additional assembly steps. We used Hi-C interaction information to call back potential Y-linked contigs that were filtered by our strict filtering on the basis of low female read depths. Arima Hi-C reads were mapped to mCalJac1 and the Hi-C interaction matrix was generated by HiCPro (v.2.10.0)^70^. At 10-kb resolution, we extracted the interaction strength of every unplaced scaffold to each autosome, X or Y chromosome. Unplaced scaffolds with more than five interaction strength values to both autosomes/X and Super_scaffold_pat_24 were selected, and the interaction strength with the autosomes/X and the interaction strength with Y was compared for each scaffold by two-sided Wilcoxon rank-sum test. With a false discovery rate (FDR)-corrected *P* value cut-off of 0.01, we further identified 17 scaffolds that show a significantly higher interaction with Super_scaffold_pat_24 than with other chromosomes, and considered them putative Y-linked scaffolds. To validate this result, we collected sequences of bacterial artificial chromosome mapped to the marmoset Y chromosome from NCBI and mapped them to mCalJac1 with minimap2. Almost all BAC sequences mapped to the eight Y-linked scaffolds were identified by the sequencing depth method. One, BAC AC279170.1, was previously missed, but can now be mapped to pat_scaffold_39_arrow_ctg1, which was identified by the Hi-C method. Thus, the dataset identified by the Hi-C method complements the dataset identified by the sequencing depth method. Combining these two datasets, a total of 25 potential Y-linked scaffolds (around 14.13 Mb) were identified from mCalJac1 (Supplementary Table 39).

Next, we mapped the PacBio raw reads to the assembly and found that some of the potentially Y-linked scaffolds had regions of considerably high coverage compared to autosomes and X chromosomes, indicative of collapsed sequences, which would cause the artificially high level of Hi-C interaction and introduce false-positive Y-linked sequences. To de-collapse these regions, we used the Segmental Duplication Assembler (SDA)^21^ and mapped the SDA-assembled contigs to their original scaffolds with minimap2 to remove potential assembly artefacts. To replace the original collapsed sequence in the assembly with the most plausible candidate de-collapsed sequence, we applied ‘the longest rule’: start with the de-collapsed sequence in the SDA output that has the longest stretch mapping back to the original scaffold, then select the second sequence with the longest match that does not overlap the previous one, and so on. Once all the non-overlapping de-collapsed sequences with the longest matches were selected, we filled in the gaps using the original scaffold as a backbone, and left 1,000 ‘N’s (gap indicating unknown nucleotides in the assembly) between each contig.

To further exclude false positives from the de-collapsed Y dataset, we refiltered the sequences with the sex-differential depth ratio and the Hi-C interaction criteria as mentioned above (Supplementary Table 24). However, as only the uniquely mapped reads were used in calculating the Hi-C interaction between unplaced scaffolds and autosomes/X/Y, our results underestimate Y-chromosomal DNA, including many de-collapsed Y scaffolds with multiple copies that might still be missed.

### Detection of SNPs, indels and SVs using whole-haplotype genome alignment

To call heterozygous sites between the two haploid sequences, independent of the GenomeScope calculation, we first performed a Mummer (v.3.23) alignment with the parameters of ‘nucmer -maxmatch -l 100 -c 500’. Because our assemblies span most repetitive sequences, repeat-masking treatment was not necessary before conducting the Mummer alignment. A series of custom scripts (https://github.com/comery/marmoset) identified and sorted our SNPs and indels in the alignments. We used svmu (v.0.4-alpha)^71^, Assemblytics (v.1.2)^72^, and SyRi (v.1.0)^73^, to detect SVs from Mummer alignment. After several test rounds, we found that svmu reported more accurate large indels, and Assemblytics detected CNVs, particularly tandem repeats, whereas SyRi detected other SVs well. We used these three methods and combined the results as confident SVs. We used default parameters for svmu, Assemblytics, and recommended nucmer alignment for SyRi (https://schneebergerlab.github.io/syri/).

To generate a high-quality SV dataset, we manually checked all inversions and translocations with the following steps: (1) clip 300 bp of upstream/downstream flanking sequence of each break point between the two haplotypes, blast against local PacBio reads with threshold identity >96% and aligned length >550 bp, and require the SV region where the maternal and paternal sequences aligned to have high similarity (>90%); (2) if (1) failed, then check the 10X linked-read count between a 5-kb flanking region; (3) if any break point is not supported by 10X linked-reads, check the Hi-C heat map of this region; if it shows an inversion or translocation pattern on heat map or an ambiguous situation, then remove it.

To evaluate the accuracy of SV detection, we searched the binned PacBio reads around the break points of both maternal and paternal assemblies for all indels in chromosome 1. We looked for one of the following three features to determine the indel as accurate: (1) at least one single PacBio long read from each haplotype that spans the entire indel region with the variation found in each haplotype; (2) overlapping PacBio reads that span the two break points; or (3) manually validated PacBio read alignment by the Integrative Genomics Viewer (IGV)^74^. Finally, we found that 95.7% of indels are correct when considering the breakage location; however, 74.2% are accurate when considering both boundary and location.

### Estimation of sequencing error and polishing error

To calculate sequencing errors and polishing errors, we established a confident SNP set as a criterion. We used three individual approaches to detect SNPs between two haplotypes: (1) retrieved heterozygous sites from the Mummer alignment between the maternal and paternal haplotypes excluding the sex chromosomes (setA, containing 3.48 million SNVs); (2) GATK pipeline based on mapping of 10X linked-reads from the F_1_ offspring (setB); and (3) SAMTools (v.1.8) mpileup followed by bcftools also based on 10X linked-reads mapping (setC). Then, a raw SNP dataset was generated by a two-step procedure: first taking the intersection of setB and setC to generate Set1 (3.72 million SNVs), followed by taking the union of setA and Set1 to get Set2 (3.77 million SNVs). We then took these two sets and selected among them to a high-quality 3.58-million SNP Set3 (Supplementary Fig. 10) with the following criteria applied: (1) 10X linked-read depth lower than 10; (2) filter out sites that do not align to the two haplotype assemblies; (3) filter out sites that we could not call a typical haplotype on the basis of much less than 50% nucleotide distribution (*π* > 0.4 and the third highest depth >5, in which *π* is calculated as: $\pi =2\times (AT+AC+AG+TC+TG+CG)/({\rm{Totaldepth}}\times ({\rm{Totaldepth}}-1))$

and *A*, *T*, *C* and *G* represent the sequencing depth of base A, T, C and G for each site. For example, a distribution of ‘A:20; T:20; C:14; G:0’ indicates a complex condition. We also collected the mapping information from raw PacBio reads and corrected PacBio reads. This allowed us to establish an evidence chain of how the bases in each haplotype changed during assembling and polishing, which allowed us to classify different error types. We classified 195,751 sequencing error sites and 180,712 polishing error sites. The sequencing and polishing error rates were estimated to be 3.41 × 10^−5^ and 3.66 × 10^−5^, respectively. We further validated the variants with PCR experiments (Supplementary Note).

### Mutation rate analysis

The 10X linked-reads of the F_1_ offspring and the parents’ short reads were mapped to each genome assembly independently (paternal and maternal assemblies). Duplicate reads and reads that mapped to more than one region were removed. Variants were called using GATK4 HaplotypeCaller in base-pair resolution mode, calling each single site of the genome. Two independent joint genotypes were produced: one for the three individuals (mother, father and F_1_ offspring) mapped to the maternal assembly and one for the three individuals mapped to the paternal assembly. We identified a maternal candidate de novo mutation as a site for which the parents were homozygous for the reference (0/0) and the offspring was heterozygous (0/1) when mapped to the paternal genome. For validation, such a candidate site would be expected to have the parents homozygous for the alternative (1/1), and the offspring heterozygous (0/1) when mapped to the maternal genome. Similarly, a paternal candidate de novo mutation was identified as a site for which the parents were homozygous for the reference (0/0), and the offspring was heterozygous (0/1) when mapped to the maternal genome. Here, again, those candidates were validated if they also appeared in the parents as homozygous for the alternative (1/1), and in the offspring heterozygous (0/1) when mapped to the paternal genome. Additional filters were applied for sites, genotype quality, read depth and number of alternative alleles in the parents and allelic balance in the offspring (Supplementary Note). Finally, we removed any potential sites with sequencing errors, polishing errors or assigning errors, as well as sites that failed the PCR validation. To calculate a rate, we computed the number of callable sites in each genome as the number of sites for which both parents were homozygous for the reference and all individuals passed the depth coverage between half and two times the average depth for each individual, number of alternative alleles allowed, and genotype quality filters. We corrected those callable sites by a negative rate factor, alpha (*α*), which is the percentage of callable sites that would be filtered away by our site filters (following a known distribution) and the allelic balance filter (which corresponds to the number of sites for which one parent was homozygous for the reference allele, the other parent was homozygous for the alternative allele, and the offspring would be heterozygous, but the reads supporting each allele would be outside our allelic balance filter). The mutation rate was calculated as:

$$\,\mu =\frac{{{\rm{Mutations}}}_{{\rm{maternal}}}+{{\rm{Mutations}}}_{{\rm{paternal}}}}{{{\rm{Callability}}}_{{\rm{maternal}}}\times (1-{\alpha }_{{\rm{maternal}}})+{{\rm{Callability}}}_{{\rm{paternal}}}\times (1-{\alpha }_{{\rm{paternal}}})}\,.$$

### Confirmation of the order of Y-linked sequences

Marmoset Y-chromosome-specific BAC end reads^22^ were obtained from the NCBI trace archive and mapped to Y-linked sequences with BWA MEM. Only the primary alignment was kept for each read. BAC location on the Y chromosome from a previous report^22^ was also obtained and visualized in a dot plot to confirm the order of the Y-linked sequences in mCalJac1. To confirm the MSSDR translocation in the Y chromosome, we further checked PacBio and 10X linked-reads support at the flanking break point of the MSSDR of the Y chromosome.

### Detection of PSGs

We used the BLAST reciprocal best hits (RBH) method (Supplementary Note) to identify high-confidence one-to-one orthologous genes among species, including three other New World monkeys (white-faced capuchin (*Cebus capucinus*), Ma’s night monkey (*Aotus nancymaae*) and black-capped squirrel monkey (*Saimiri boliviensis*)); three old world primates (human (*Homo sapiens*), macaque (*Macaca mulatta*) and chimpanzee (*Pan troglodytes*)); and three outgroups (treeshrew (*Tupaia glis*), mouse (*Mus musculus*) and cow (*Bos taurus*)). The marmoset was set as foreground when detecting marmoset-specific PSGs, whereas the New World monkeys were set as foreground when detecting New World monkey-specific PSGs. A total of 13,995 one-to-one orthologous genes were identified. To minimize the effect of gene annotation, we retrieved the corresponding coding sequences that shared the same isoform with human. These genes were used as an input dataset to conduct multiple sequence alignment using PRANK (v.170427)^75^ and guidance (v.2.02)^76^ to improve the alignment. The positive selection sites within a specific lineage were detected by branch-site model in PAML (v.4.9i)^77^. Genes with an FDR-adjusted *P* value of less than 0.05 were treated as candidates for positive selection. To minimize effects of assembly and alignment, we filtered candidate PSGs if (1) the positively selected site has gaps in more than two species; (2) the positively selected sites had more than two non-synonymous substitution forms (ignoring outgroup), and (3) the flanking region (±10 amino acids) showed over-alignment across species. We also performed a manual check for all individual PSGs to avoid any other false-positive caused by annotation or alignment. Finally, we used read mapping to check the PSG sites to avoid sequencing errors. After filtering, the numbers of PSGs with high confidence detected in marmosets and New World monkeys were 204 and 38, respectively.

### Scan for pathogenic or risky mutations in marmosets

Mutation information was obtained from ClinVar (https://ftp.ncbi.nlm.nih.gov/pub/clinvar/tab_delimited/variant_summary.txt.gz, on 30 June 2020) and mutations that were designated to be pathogenic or risky were extracted. Nervous-system-related mutations were extracted with the following keywords: adrenoleukodystrophy, Alzheimer, amyotrophic lateral sclerosis, Angelman, ataxia telangiectasia, Charcot-Marie-Tooth, Cockayne, deafness, Duchenne muscular dystrophy, epilepsy, fragile X syndrome, Friedreich ataxia, Gaucher, Huntington, Lesch-Nyhan syndrome, maple syrup urine disease, Menkes syndrome, myotonic dystrophy, narcolepsy, neurofibromatosis, Niemann-Pick disease, Parkinson disease, phenylketonuria, Refsum disease, Rett syndrome, spinal muscular, spinocerebellar ataxia, Tangier disease, Tay-Sachs disease, tuberous sclerosis, Von Hippel-Lindau syndrome, Wilson disease. Related protein sequences of humans and marmosets were extracted and aligned with PRANK and targeted amino acid sites were scanned to determine whether the human pathogenic or risky mutation is in the marmoset. The genomic coordinates of related codons were extracted to check the alignment of the 12 marmoset individuals with whole-genome-sequencing data. Alignment was visualized and manually examined with Jalview (v.2.11.1.0)^78^.

### Reporting summary

Further information on research design is available in the Nature Research Reporting Summary linked to this paper.

## Online content

Any methods, additional references, Nature Research reporting summaries, source data, extended data, supplementary information, acknowledgements, peer review information; details of author contributions and competing interests; and statements of data and code availability are available at 10.1038/s41586-021-03535-x.

## Supplementary information

Supplementary Information This file contains details on the sample collection and methods used in this study. It also includes Supplementary Notes with the detailed analyses results, Supplementary Figures 1-36 and descriptions for Supplementary Tables 1-39 (Supplementary Tables supplied separately).

Reporting Summary

Supplementary Tables This file contains Supplementary Tables 1-39 – see Supplementary Information document for full descriptions.

## Data Availability

Raw sequencing data for the marmoset trio is available under the GenomeArk github (https://vgp.github.io/genomeark/Callithrix_jacchus/). Curatorial information and data mappings to maternal and paternal assemblies are available on the genome evaluation browser, gEVAL (https://vgp-geval.sanger.ac.uk/all_genomes.html). The maternal, paternal, and combined (paternal autosomes and Y chromosome + maternal X chromosome + mitochondrial) assemblies, as well as PacBio Iso-Seq data for annotation, are available under the NCBI BioProject PRJNA560230. The genome assemblies have also been deposited at the CNGB Sequence Archive (CNSA) of the China National GeneBank Database (CNGBdb) with accession numbers CNP0001310 and CNP0001311.

## Code availability

The assembly pipeline is available at https://github.com/VGP/vgp-assembly; see Supplementary Tables 2, 3 for the full list of tools used, versions and availability. Workflows and applets built for the VGP are available at DNAnexus (https://www.dnanexus.com/). Custom scripts are available at https://github.com/comery/marmoset and https://github.com/gf777/misc/tree/master/marmoset%20Y.
