# Supplementary material for: Evolutionary and biomedical insights from a marmoset diploid genome assembly
Source: Nature. 2021 Apr 28;594(7862):227–33. doi: 10.1038/s41586-021-03535-x (PMC8189906; doi:10.1038/s41586-021-03535-x)
Supplement: Supplementary file 1 — This file contains details on the sample collection and methods used in this study. It also includes Supplementary Notes with the detailed analyses results, Supplementary Figures 1-36 and descriptions for Supplementary Tables 1-39 (Supplementary Tables supplied separately). [file 41586_2021_3535_MOESM1_ESM.docx]

# **Supplementary Note**

##

## Tissue sample collection

We collected samples at The Rockefeller University, USA, under USDA and IACUC approved protocols established at the university. Two adult marmosets were mate-paired (male/father = mCalJac2, female/mother = mCalJac3; mCalJac2 was one of triplets, whereas mCalJac3 was a singlet birth). mCalJac3 became pregnant and gave birth to triplets. Muscle, liver, and spleen were collected from one of their male offspring (mCalJac1) at three months of age (**Supplementary Table 1**). Tissue was subdivided into roughly 50 mg chunks, flash frozen in liquid nitrogen, and stored in cryotubes on dry ice while transferred to permanent storage at -80°C.

We subsequently drew blood samples from mCalJac2 and mCalJac3 via saphenous and tail veins. Blood from mCalJac3 was drawn three weeks following a second pregnancy with triplets. All blood samples were collected in EDTA vials and placed in dry ice before being transferred to permanent storage at -80°C.

For annotation purposes, a second male offspring (mCalJac4, brother of mCalJac1), was euthanized, perfused, and >18 tissues collected: blood, testes, brain, muscle, kidney, lung, liver + gallbladder, eye, heart, pancreas, stomach, spleen, tongue, trachea, esophagus, bone marrow, intestine + duodenum + rectum, and skin. Blood was collected in EDTA tubes and stored on dry ice. We sectioned all other tissues into chunks, flash frozen in either liquid nitrogen or powdered dry ice, and stored in cryotubes on dry ice. Brain tissue was collected then sliced in two-millimeter coronal sections on a 3D-printed marmoset brain mold. We flash frozen sections with powdered dry ice. All tissues were transferred to permanent storage at -80°C.

##

## Sample processing and sequencing

##

## gDNA extraction:

## Muscle tissue was dissected, flash frozen in liquid nitrogen, and stored at -80°C until time of processing (see sample collection). Ultra-high molecular weight (uHMW) DNA was extracted using the Bionano Prep Animal Tissue DNA Isolation Fibrous Tissue Protocol (Document Number 30071). A total of approximately 300mg of tissue was used for multiple iterations of DNA isolation. For each extraction, 40-60mg of frozen tissue was chopped into 3mm pieces and fixed with 2% formaldehyde and Bionano Prep Animal Tissue Homogenization Buffer. Briefly, tissue was blended into a homogenate with a Qiagen Rotor-Stator homogenizer and embedded in 2% agarose plugs. Plugs were treated with Proteinase K and RNase A, and washed with 1x Bionano Prep Wash Buffer and 1X TE Buffer (pH 8.0). DNA was recovered with 2µl of 0.5 U/µl Agarase enzyme per plug for 45 minutes at 43°C and further purified by drop dialysis with 1X TE Buffer.

## Bionano optical mapping:

750µg of the extracted DNA was labeled with a direct labeling enzyme (DLE-1) following the Bionano Prep Direct Label and Stain (DLS) Protocol (Document Number 30206). Labelled samples were imaged on the Bionano Saphyr instrument.

### **PacBio library preparation:**

16µg of uHMW DNA was sheared using a 26G blunt end needle (PacBio protocol PN 101-181-000 Version 05). Two large-insert PacBio library were prepared using the Pacific Biosciences Express Template Prep Kit v2.0 (#100-938-900) following the manufacturer protocol. The library was then size selected (>20kb) using the Sage Science BluePippin Size-Selection System.

### **PacBio SMRT sequencing:**

PacBio Library was sequenced on 33 PacBio 1M v2 (#101-008-000) smartcells on the Sequel instrument with the sequencing kit 2.1 (#101-310-500) and 10 hours movie time to a total of 218.92 Gb. A necessary limitation of the binning approach is that in generating two separate, haplotype-specific assemblies, we essentially halve the PacBio read coverage. It was therefore imperative that we begin the assembly with a high read coverage in order to compensate for binning, allowing us to assemble repetitive regions, sex chromosomes, etc.

### **10X Genomics sequencing:**

Unfragmented uHMW DNA was used to generate a linked-reads library on the 10X Genomics Chromium (Genome Library Kit & Gel Bead Kit v2 PN-120258, Genome Chip Kit v2 PN-120257, i7 Multiplex Kit PN-120262). We sequenced this 10X library on an Illumina Novaseq S4 150bp PE lane (~60X coverage).

### **PCR-free whole genome sequencing / Illumina:**

PCR-free libraries were generated from 1 µg genomic DNA using a Covaris LE220-plus to shear the DNA and the TruSeq® DNA PCR-Free HT Sample Preparation Kit (Illumina) for library generation. The median insert sizes were approximately 400 bp. Libraries were tagged with unique dual index DNA barcodes to allow pooling of libraries and minimize the impact of barcode hopping. Libraries were pooled for sequencing on the NovaSeq 6000 (Illumina) to obtain at least 750 million 151-base read pairs per individual library.

### **PacBio SMRT sequencing for IsoSeq analysis:**

We selected testes, lung, and six brain regions – primary motor cortex, hippocampus, caudate nucleus + putamen, thalamus, brainstem, and cerebellum – of mCalJac4 for sequencing. We then microdissected brain regions from 2mm coronal sections on a chilled steel block. Total RNA extraction and purification was conducted with QIAGEN RNAeasy Protect kit (Cat. No 74124). 25-30mg of frozen tissue was kept on dry ice and cut into 2mm pieces before being disrupted and homogenized with the Qiagen TissueRuptor II (Cat No./ID: 9002755). The quality of all RNAs were measured using a Fragment Analyzer (Agilent Technologies, Santa Clara, CA) and quantified with a Qubit 3 Fluorometer (Qubit™ RNA BR Assay Kit - Catalog number: Q33216); RINs were within 8.2-9.9.

PacBio Iso-seq libraries were prepared according to the ‘Procedure & Checklist – Iso-Seq™ Express Template Preparation for Sequel® and Sequel II Systems’ (PN 101-763-800 Version 01). Briefly, cDNA was reverse transcribed using the NEBNext® Single Cell/Low Input cDNA Synthesis & Amplification Module (New England BioLabs, cat. no. E6421S) and Iso-Seq Express Oligo Kit (PacBio PN 10 1-737-500) from 320-420µg total RNA. Forward and reverse barcoded primers were used during cDNA amplification. Amplified cDNA was cleaned with 86μL ProNex® Beads (Promega - Catalog numbers: NG2001). PacBio Iso-seq libraries were sequenced on 3 PacBio 8M (PN: 101-389-001) SMRT Cells on the Sequel II instrument with Sequencing Kit 1.0 (PN: 101-746-800) using the Sequel® II Binding Kit 1.0 (PN: 101-726-700) and 30 hours movie with 2 hours pre-extension. Samples were multiplexed as follows: cell 1 = testes + lung, cell 2 = brainstem + cerebellum + motor cortex, cell 3 = striatum + hippocampus + thalamus.

### **Quality control of sequencing data**

In order to assure no contamination of the raw sequencing data occurred prior to assembly, we screened maternal and paternal Illumina, F1 PacBio, and F1 10x-linked sequencing reads for outliers using Mashmap (v2.0)^1^. Mash takes a k-mer approach to computing alignment boundaries and identity estimates; it quickly estimates sequence similarity rather than computing exact alignment. For each animal:technology combination, we ran the output libraries of individual sequencing cells against themselves and every other cell; we used 21-mers and a sketch size of 10,000:

$tools/mash/mash sketch -k 21 -s 10000 -r -m 1 -o $output_file $input_file

We found no contamination of sequencing reads **(Supplementary Fig. 1).**

##

## Genome size estimation

We used a k-mer approach to estimate the haploid genome length, heterozygosity, and repeat length of the genome with the F1 10x linked sequencing libraries. After trimming GEM barcodes from the 10x reads using proc10xG (v0.0.2), we collected 31-mers across all 10x reads from the F1 with meryl (v1.0). From the histogram of the resulting 31-mers, we then ran GenomeScope (v1.0)^2^ for assembly estimations (**Extended Data Fig. 1**). The haploid genome length estimated here was used in all subsequent assembly steps within the pipeline that require genome size as a standard parameter.

## Haplotype phased assembly

Haplotype phased assembly of the common marmoset genome was based upon combining the trio binning approach developed by Koren et al.^3^ and the Vertebrate Genomes Project (VGP) assembly pipeline^4^ **(Supplementary Fig. 2).**

### **Trio binning and contigging (c1):**

We generated haplotype-specific k-mers from the parental Illumina short reads in order to classify the offspring’s PacBio long reads into maternal and paternal bins for individual assembly. Triocanu generated the haplotype specific contigs (haplotigs) from the binned F1 PacBio long reads. Triocanu also identified long reads ambiguous to either haplotype. However, these reads fell below the threshold for use; unbinned reads were not used in the generation of the maternal or paternal assemblies.

Haplotype binning and the paternal haplotigs were generated with the following command:

canu -p asm -d canu1.8+276 genomeSize=3.43g -haplotypePaternal paternal/*.fastq.gz -haplotypeMaternal maternal/*.fastq.gz -pacbio-raw pacbio/*subreads.fasta.gz useGrid=true gridOptions="--time=3-0" stageDirectory="/lscratch/\$SLURM_JOBID" gridEngineStageOption="--gres=lscratch:100" hapMemory=48 batMemory=128g corMinCoverage=0 correctedErrorRate=0.105 hapUnknownFraction=0.20

We dropped the maternal haplotig assembly from the above output due to high sensitivity and shattered contig continuity, caused by a coverage drop. Instead, we used the following command for generating the maternal haplotype assembly with normal sensitivity:

canu -d canu_mat_sk -p 'asm' 'genomeSize=3.43g' 'gridOptions=--time=3-0' 'stageDirectory=/lscratch/$SLURM_JOBID' 'gridEngineStageOption=--gres=lscratch:100' 'batMemory=128g' 'corMinCoverage=0' 'correctedErrorRate=0.105' 'corMhapSensitivity=normal' -pacbio-raw '$/intermediates/triocanu/canu1.8+276/haplotype/haplotype-Maternal.fasta.gz'

### **Mitochondrial assembly (MT Genome):**

In order to keep raw mitochondrial (MT) reads from being attracted to nuclear sequences during polishing, which could result in improper base calling, we assembled the MT genome separately and concatenated the linearized version to the maternal and paternal haplotig fasta files prior to the first polishing step. MT assembly was completed using the mitoVGP pipeline developed by Formenti et al.^5^.

### **Polishing (c2):**

In order to improve base-pair accuracy, we ran haplotigs through a polishing step. Raw PacBio long-reads of the F1 (mCalJac1) were aligned to the maternal and paternal haplotigs (*.c1.fasta) with Minimap 2 (pbmm2); base-pair errors with haplotigs were polished via consensus base calling with PacBio’s Arrow algorithm.

### **Purging (p):**

We used the purge_dups tool^6^ to remove false duplications within the contig overlap regions of our haplotigs. For both haplotypes, purge_dups was run with a max genome size (Gb) of 4, in the haploid ploidy mode, and to remove overlaps only. Following purging, we saw increases in QV (maternal: 37.29 -> 37.38 and paternal: 37.66 -> 37.73).

### **Scaffolding**

#### **10x (s1):**

Using the p1 haplotigs, we then generated scaffolds via 10x linked reads through two rounds of scaffolding with Scaff10x. 10x reads were mapped to the purged haplotigs using BWA. Barcode tags extracted from the 10x reads (R1 and R2 .fastq inputs) allow for building a relationship matrix among shared barcodes that indicate contigs/haplotigs may be near neighbors or linked. Output of the first round of Scaff10x was used as input for the second round (rather than the raw 10x reads). Break10x was not used. Parameters were set as follows:

/bin/scaff10x -nodes 32 -longread 1 -gap 100 -matrix 2000 -read-s1 12 -read-s2 8 -link 10 -score 20 -edge 50000 -block 50000 -align bwa

####

#### **Bionano (s2):**

We continued to resolve scaffolds through Bionano optical mapping data, which can help to break previous misjoins and independently detect structural variants. Using cmaps generated from the raw bionano data (DLE-1 one enzyme approach) and the s1 scaffolds as inputs, we ran the Bionano Solve 3.2.1 pipeline with the following parameters:

Conflict Resolution Filter Level for Bionano Contigs: 2

Conflict Resolution Filter Level for NGS Contigs: 2

Generate Molecules: FALSE

Generate Chimeric Quality Score: FALSE

perl /Solve3.2.1_04122018/HybridScaffold/04122018/hybridScaffold.pl -n $ref -b $cmap -o $out -c /Solve3.2.1_04122018/HybridScaffold/04122018/hybridScaffold_config.xml -r /Solve3.2.1_04122018/RefAligner/7437.7523rel/RefAligner -B 2 -N 2

Solve estimated gap sizes from the alignment of sequences to the hybrid scaffolds. In the resulting s2 scaffolds, we saw an expected decrease in contig N50 due to breaking of previous misjoins (maternal: 8.718 Mb -> 8.002 Mb, paternal: 14.033 Mb -> 12.030 Mb).

#### **Hi-C (s3):**

Finally, we used contact maps generated from Arima Hi-C reads to complete the third round of scaffolding. We completed mapping using the Arima Hi-C Mapping Pipeline; forward and reverse reads of Hi-C read pairs were independently mapped to each of the two haplotypes’ s2 scaffolds with BWA MEM. Resultant files were converted to BAM format and filtered for chimeric reads (single-end reads that come from >1 contiguous DNA sequences) before merging. Bamsormadup was then used for sorting, de-duplication, and index file generation. BWA MEM was run with the parameter -B 8:

#For reads

bwa mem -t 32 -B 8 $ref $in_dir/$sra\_1.fastq.gz | perl ./filter_five_end.pl | $SAMTOOLS view -@$cpu -Sb - > $raw_dir/$sra\_1.bam

#Rev reads

bwa mem -t 32 -B 8 $ref $in_dir/$sra\_2.fastq.gz | perl ./filter_five_end.pl | $SAMTOOLS view -@$cpu -Sb - > $raw_dir/$sra\_2.bam

The output contact maps were then used for scaffolding via Salsa2^7^, which orders and orients contigs by analyzing the frequency of interactions between all contig pairs. We specified the restriction enzyme bases used to generate the library with the parameter -e GATC, GANTC; parameter -m yes was used to find/break mis-assemblies in the s2 input scaffolds and -i 5 -p yes were used to perform five iterations of scaffolding using each output as the subsequent iteration input:

python /opt/SALSA/run_pipeline.py -a $fasta -b $bed -l $fasta.fai -o $out -e GATC,GANTC -m yes -i 5 -p yes

The final scaffold assembly generated from the Arima Hi-C data was named s3; pretext visualizations were generated to evaluate contact maps of both the s3 output and the final curated output (**Supplementary Fig. 3**).

### **Gap-filling and polishing** **(t1-t3):**

We ran the final s3 scaffolds of each haplotype through another round of gap filling and base-error polishing with Arrow using the PacBio long-reads. Minimap2 was used for read alignment; biobambam2 was used for sorting, indexing, and marking duplicates and pbbamify was used to enable compatibility with Arrow downstream. For Arrow, we used a minimum coverage of 5, minimum confidence of 40, and masking (to omit regions during polishing that have low template concordance).

The subsequent t1 outputs from both haplotypes were merged, then run through two rounds of polishing (t2,t3) with FreeBayes using the 10x-linked short reads. We used 10x Longranger for read alignment; creating a reference with longranger mkref then aligning the 10x *.fastq files to the reference with longranger align. We used default parameters for FreeBayes to call variants, then bcftools to generate the consensus:

bcftools consensus Oz -i'QUAL>1 && (GT="AA" || GT="Aa")' -Hla -f $fasta $sample.bcf > $sample.fasta

We separated scaffolds by haplotype into individual assemblies and removed the mitochondrial genome representations prior to manual curation.

### **Curation**

Both the maternal and paternal assemblies were manually curated for assembly errors by collating, aligning, and analyzing all available data – generated for these assemblies and publicly available – within the genome evaluation browser, gEVAL (<https://vgp-geval.sanger.ac.uk/index.html>)^8^. A fully detailed description of curation methods, including for chromosome assignment, is described in Rhie et al.^4^. Specifics of curation are as follows:

Paternal: A contamination check identified vector sequence (two scaffolds) and mitochondrial sequence (one scaffold) plus trailing Ns (nine scaffolds) which were removed. Subsequent manual assembly curation corrected 163 missing/misjoins and removed 553 haplotypic duplications, reducing the scaffold number by 58% and increasing the scaffold N50 by 5.4% The final assembly has a total length of 2.69 Gb in 463 sequence scaffolds with a scaffold N50 of 137 Mb. The majority, 98.9%, of the assembly sequence was assigned to 23 chromosomal-level scaffolds representing 22 autosomes (named by synteny to GCA_000004665.1), and the Y chromosome.

Maternal: A contamination check identified vector sequence (two scaffolds) and mitochondrial sequence (two scaffolds) plus trailing Ns (one scaffold) which were removed. Subsequent manual assembly curation corrected 127 missing/misjoins and removed 507 haplotypic duplications, reducing the scaffold number by 72% and increasing the scaffold N50 by 15.3% The final assembly has a total length of 2.81 Gb in 216 sequence scaffolds with a scaffold N50 of 146.9 Mb. The majority, 99.45%, of the assembly sequence was assigned to 23 chromosomal-level scaffolds representing 22 autosomes (named by synteny to GCA_000004665.1), and the X chromosome.

## Assembly Statistics and Evaluation

Following each stage of the assembly, we calculated, where applicable, N50, NG50, number of contigs, number of scaffolds, number of gaps, and quality value (QV) scores for each base call (**Supplementary Table 8**).

Five different published marmoset karyotypes were estimated for length^9-12^ based on the GenomeScope-estimated genome length of 3,068,578,524 bp (**Extended Data Fig. 1**). Due to image quality, karyotype images were processed in the following manner: 1) lines, labels, and descriptions removed, 2) color inverted, and 3) background removed. Background removal was necessary, especially for karyotypes 1./2./3., where the pipeline’s threshold filter could not differentiate between chromosome and background. Python code for generation of the chromosome size estimates from karyotype images can be found in the supplementary methods of Rhie et al.^4^. Three different estimations were made on: 1) the gray image (image generated from the 3 processing steps), 2) a threshold filtered image, and 3) a threshold filtered and filled image (object detection followed by black fill) (**Supplementary Fig. 5**). The fill image slightly outperformed the other two. Individual chromosome size estimations can be found in **Supplementary Table 4**, and final karyotype-estimated length to assembly length correlations found in **Supplementary Table 5**. Assemblies correlated most closely to karyotype 3^11^, visualized as the regression plot (exact assembled chromosome lengths vs estimated average fill length) for **Extended Data Fig. 2b**.

We used Merqury (v1.0) for overall assembly evaluations (including k-mer completeness and spectra copy number analysis) as well as phasing assessment with hap-mers (**Supplementary Fig. 7**). We first generated 21-mer databases (dbs) from the raw F1 10x data and the parental Illumina data using meryl. We then built inherited hap-mer dbs by taking the difference between the maternal and paternal k-mer dbs, filtering according to the filter level used by TrioCanu for binning, intersecting both with the F1 db, and filtering again, as below (steps 1-4).

1. Generating the maternal and paternal specific dbs:

meryl difference pat_illumina_db_k21.meryl mat_illumina_db_k21.meryl output pat_minus_mat.meryl

meryl difference mat_illumina_db_k21.meryl pat_illumina_db_k21.meryl output mat_minus_pat.meryl

2. Filtering according to the level used by TrioCanu when binning with the parental specific k-mers:

meryl greater-than 20 pat_minus_mat.meryl output pat_minus_mat_gt20.meryl

meryl greater-than 18 mat_minus_pat.meryl output mat_minus_pat_gt18.meryl

3. Intersecting the offspring’s db with the parental specific dbs to generate the parental specific inherited dbs:

meryl intersect mCalJac1.k21.meryl pat_minus_mat.gt20.meryl output pat_gt20_inherited.meryl

meryl intersect mCalJac1.k21.meryl mat_minus_pat_gt18.meryl output mat_gt18_inherited.meryl

4. Final filtering:

meryl greater-than 6 pat_inherited.meryl output pat_gt20_inherited_gt6.meryl

meryl greater-than 6 mat_inherited.meryl output mat_gt18_inherited_gt6.meryl

Using all three meryl dbs generated (F1 and the two inherited hamper dbs), we ran Merqury as a diploid assembly:

./merqury.sh $f1.meryl $mat.meryl $pat.meryl $asm_mat.fasta $asm_pat.fasta out $name

Switch error rate is an artifact of haplotype-phased genome assemblies, rather than a biological phenomenon. To our knowledge, there are currently no other phased non-human primate assemblies with raw datasets that would allow for direct comparison. However, Merqury analysis of the most recently published, phased human assembly (for individual ‘HG00733’) reports a switch error rate of 0.0782165% for the paternal assembly and 0.0792735% for the paternal alternate assembly^13^. With a switch error rate of 0.1383% for paternal and 0.2417% for maternal assemblies, our results in marmoset are on par with methods developed in human genetics, for which there is historically more information as well as algorithms tailored specifically for human data. Additionally, our approach does not require the generation of cell lines. Merqury analysis also shows that there are more unique k-mers in the maternal assembly than the paternal assembly, which we expect given the significantly larger maternal X chromosome compared to the paternal Y (**Supplementary Fig. 7**).

For evaluation of genome completeness / gene content, we ran BUSCO on our assemblies to determine the representation of near-universal single-copy orthologs in the vertebrate lineage (**Supplementary Table 9**): python run_BUSCO.py -i input.fasta -o output.name -l vertebrata_odb9/ -m genome -c 16 --limit 5 -sp human. The result shows a high completeness of gene content in both assemblies (**Supplementary Table 9**); 94.8% (maternal) and 93.7% (paternal) of the total 2,586 orthologous genes searched.

## Estimation of chimeric level and its effect on mCalJac1

To estimate the chimeric level and its effect on mCalJac1, we utilized SNV phasing information to identify all possible alleles for each individual (offspring, mother, father). Phasing was performed in a non-overlapping sliding-window way for each genomic region spanning two SNVs. The analysis in offspring was based on 10X-linked reads, while mother and father samples were based on their Illumina resequencing reads (PE151). The N50s of the genomic region spanning two SNVs are 2.8 kb, 276 bp, and 275 bp in offspring, mother, and father, respectively. In principle, for each window containing two SNVs, we should be able to detect at most four alleles, and a window is assigned as chimeric if it contains more than two types of alleles. We calculate the number of reads’ support for each allele. And based on the supporting rank for each allele, the top two alleles are considered as major alleles and the other two are third/fourth alleles. Two diploid penguin species (*Eudyptula novaehollandiae* and *Eudyptula minor albosignata*) are also used as a control here as they are egg-laying and should contain no chimerism. Reads were obtained from NCBI (SRX6614012, SRX6614006, SRX6614005 for *Eudyptula novaehollandiae* and SRX6614041, SRX6613980, SRX6613971 for *Eudyptula minor albosignata*) and mapped to their genomes (GCA_010078495.1, GCA_010080465.1), respectively. SNV calling and allele calculation of each window were performed with the same strategy as in marmoset.

The majority of regions were found to contain no more than two types of alleles (**Supplementary Fig. 6c**). We evaluated the proportion of regions containing more than two types of alleles, which might be attributed by either chimerism or sequencing errors. This resulted in a genomic proportion of 3.69% in offspring, 1.76% in mother, and 19.81% in father (**Supplementary Table 6**). This ratio might be underestimated because many genomic regions are homozygous in all siblings thus could not be used to distinguish different types of alleles.

We further used the regions with four alleles to estimate the chimeric level based on the coverage of these alleles, calculated as the mean ratio of the alleles at the third and the fourth ranks (**Supplementary Fig. 6b, c**). By doing so, we estimated that the chimeric level of the offspring is at around 7%, which means that 7% of the cells we sequenced in mCalJac1 might come from its sibling.

During the trio-binning process, some Pacbio reads derived from the third/fourth alleles might be selected as parental specific alleles and thus were used in the assembly process. In principle, these reads will have lower chance of being assembled because of the low-depth sequencing. However, because the sequencing might not be evenly distributed among alleles, it is possible that some of the third/fourth alleles can be sequenced in relative high depth and thus were used in assembly. We therefore estimated proportional error in the paternal and maternal assemblies by identifying the regions incorrectly presenting as the third/fourth alleles (**Supplementary Fig. 6d**). Based on the method above, we estimated that ca. 0.068% of maternal and 0.076% of paternal assemblies were presented in third/fourth alleles (**Supplementary Table 7**). The proportions are similar among chromosomes.

However, we found that the average depth of the chimeric regions tend to have higher depth than the non-chimeric regions (**Supplementary Fig. 6e, f)**. Thus, the chimeric level might be overestimated.

## Genome assembly comparisons

To assess the quality of our assembled haploids, we first compared genome-wide alignments of mCalJac1.mat and mCalJac1.pat formed by Mummer (v3.23)^14^ with default parameters. As they showed high structural similarity, we selected the maternal assembly, which had fewer gaps, as the representative of mCalJac1 to compare with cj1700 and cj2019. Any two of the three assemblies were also aligned by Mummer. Interchromosomal or intrachromosomal structural variations larger than 1 Mb were then detected by the Mummer dotplot and the breakpoint regions in mCalJac1.mat were manually examined and extracted from the alignment. PacBio subreads, 10X linked-reads, Bionano molecules, and Hi-C reads were aligned to mCalJac1.mat by minimap2 (v2.13)^15^, BWA MEM (v0.7.17-r1188)^16^, refaligner (7437.7523rel), and HiCPro (v2.10.0)^17^, respectively, with default parameters. The read mapping was used to confirm or reject breakpoint regions and other structural variations.

*Experimental validation of variants:* To avoid the repetitive sequences that influence the PCR efficiency, we excluded the SNPs located in repeat elements. For the remaining SNPs, we retrieved ~300 bp of its flanking sequence allowing two extra SNPs in PCR regions, and designed primer pairs for target SNP using the web-based tool BatchPrimer3 (v1.0)^18^, with custom settings of “product size, min: 400 bp; opt: 500 bp; max: 600 bp; primer size, min: 18 bp; opt: 24 bp; max: 27 bp; primer Tm, min: 57 °C; opt: 60 °C; max: 66 °C, max Tm difference: 4 °C; GC content, min: 40%; max: 60%, max-ploy-x: 4 bp”. The high specificity primers were selected using BLASTN (v2.9.0+)^19^ with the setting of “-task blastn, identity < 80%”. Then, a total of 233 SNVs were randomly selected for genome coordination to conduct experimental validation using Linux command “sort -R” from the list. These included six SNVs (including two de novo mutations) and fourteen INDELs located in ROH regions. In addition, to validate the 9 de novo mutations as heterozygous in the F1 offspring, and homozygous in the father and mother, we performed another round of primer design extending the flanking sequence to ~400 bp to prevent failure due to improper primer binding or low specificity. Additionally, we randomly selected 17 large indels from chr1 to conduct PCR and visualized by agarose gel electrophoresis.

The PCR amplifications (96-well format) were performed using a Veriti® 96-Well Thermal Cycler in a 25 µL volume containing 3-5 ng of template DNA, 10 mM of each primer, 2.5 µL 10× PCR buffer (50 mM KCl, 10 mM Tris-HCl, pH 8.3), 2 µLl dNTPs (10mM), and 0.2 µL ExTaq DNA polymerase. PCR conditions included an initial denaturation step at 94 °C for 2 min followed by 30 cycles of denaturation at 94 °C for 15 s, annealing at 52 °C for 30 s, and extension at 72 °C for 30 s, with a final extension step at 72 °C for 5 min. We fractionated 5 µL of each reaction in a 2% agarose gel containing 0.1 µg/mL SYBR® Safe DNA Gel Stain at 180 V for 30 min and visualized the amplicons with UV fluorescence.

## Genome evaluation by IsoSeq

We used IsoSeq reads to evaluate the assembly integrity of the expressed genes among the three assemblies produced in this study (paternal, maternal, and their combined genomes) as well as five published marmoset assemblies (GCA_000004665.1, GCA_001269965.1, GCA_002754865.1, GCA_009663435.1, GCA_009811775.1). In order to identify missing or partially assembled genes, IsoSeq reads were firstly clustered to remove redundant transcripts by cd-hit (v4.8.1)^20^ with parameter “-c 0.99 -T 6 -G 0 -aL 0.90 -AL 100 -aS 0.99 -AS 30 -M 0”. The remaining representative transcripts were mapped on each of the eight genome assemblies using minimap2 with parameter “-ax splice -uf --secondary=no -C5 -O6,24 -B4 --cs=long”. When either the identity or alignment rate of a transcript was less than 90%, the gene for that transcript was considered as an incomplete/missing candidate for the marmoset assembly. We then aligned these IsoSeq reads to the human genome (GCRhg38) with BLASTN (v2.7.1)^21^ and identified the genes corresponding to these reads by their overlap with the human genomic region. These human genes were used to annotate in each of the eight marmoset assemblies by GeneWise (v2.4.1)^22^ and exonerate (v2.2.0)^23^. Annotated marmoset proteins were aligned to the homologous human proteins to confirm the correctness. Genes that were not annotated by either method were considered missing in the marmoset assembly. If the alignment rate to human protein was less 90%, its gene assembly was also regarded as potentially fragmented.

##

## Evaluation of detected variation

## To evaluate the accuracy of different variations, we used both the 10X linked-reads and PacBio to reconfirm SNVs, indels, and breakpoints of SVs (Supplementary Fig. 10). About 95.2% SNPs were consistent with the “gold standard” - Set3 described above. However, these consistent sites accounted for 89.2% of Set3. Of the remaining 10.9% SNVs, about 96.7% of them were polishing and sequencing errors. Interestingly, these error sites were more likely to be located in low complexity repeats, which revealed repeats are still the most challenging problem for assembly and variation detection. We also evaluated the concordance for indels. We divided indels into three types: small indels, partial large indels in coordinate alignment, and other large indels in non-coordinate regions. Given the small indels occupied were the largest fraction of the indel dataset, and Illumina reads are limited to a size of ~50 bp and cannot detect longer indels accurately, we investigated indels with size 1~50 bp using 10X linked-reads and large indels using PacBio reads. If an indel was supported by more than three 10X linked-reads or PacBio long reads, we regarded it as a confident indel. Using this strategy, about 22.1% of small indels and 5.7% of large indels were verified.

## SNP, indels, and SV detection using read mapping

In addition to using the global alignment of two haplotypes to detect genetic variants, we mapped the 10X linked-reads and PacBio reads to our two assemblies. We used BWA MEM to align all 10X linked-reads to the maternal assembly, GATK MarkDuplicates (v4.1.4.1)^24^ to identify and remove duplicates, and SAMTools (v1.8)^25^ to sort the alignment. After merging data from the two alignments (10x and PacBio), GATK was used to detect variants. In addition, we performed PacBio read mapping using NGMLR (v0.2.7)^26^ with default parameters, and four variant categories: 1) raw PacBio reads to maternal assembly (RP2M); 2) raw PacBio reads to paternal assembly (RP2P); 3) corrected and phased paternal PacBio reads to maternal assembly (CPP2P); 4) corrected and phased maternal PacBio reads to maternal assembly (CMP2M).

## Runs of homozygosity (ROH)

To measure ROH, we performed a sliding window approach (100 kb window and 20 kb step) on the Mummer alignment between the two haplotypes. We considered windows as ROH, if they had less than a 0.02% SNP rate. To further evaluate the ROH, we used 9 published re-sequencing datasets^27^, and combined with our trio samples to calculate ROH ratios. We applied the BWA MEM and HaplotypeCaller (GATK v4.1.4.1) for genotype calling of each individual and then combined all 11 genome vcf files (GVCF) using Sentieon (v201911)^28^ with the following parameters: “--call_conf 30 --emit_conf 30”. SNPs and indels were selected using SelectVariants and then indels were filtered with parameters "QD < 2.0 || FS > 200.0 || SOR > 10.0 || MQRankSum < -12.5 || ReadPosRankSum < -8.0". After that, we filtered out low-confidential variants using GATK with the following criteria: 1) filter parameters “QD < 2.0 || DP < 50 || DP > 1500 || FS > 60 || SOR > 4.0 || MQRankSum < -12.5 || ReadPosRankSum < -8.0 ||AN<22“; 2) minimum base quality of Q30; 3) minimum distance between contiguous variants of 10 bp; 4) three bases around the indels were also masked; and 5) maximum and minimum allele number for each loci of 2. After filtering, the VCF file contained 11,953,358 variants with a Ts/Tv value of 2.01. Then, BCFtools (v1.8)^29^ was used to merge it and the SNP data set from our offspring individual to a new combined file. Finally, the final VCF file for all 12 samples was obtained after filtering low quality variations with the following criteria: 1) no missing data in the pooled population; 2) minor allele frequency (MAF) of 0.2 in the pooled population; 3) sites with a p-value of deviation from Hardy-Weinberg Equilibrium test below 0.001 excluded; and 4) all variants on the sex chromosomes removed. For each individual, the SNP set was also generated from the GATK result with the following filtering parameters “QD < 2.0 || MQ < 40.0 || FS > 60.0 || SOR > 3.0 || MQRankSum < -12.5 || ReadPosRankSum < -8.0” and used to count the variant number. The distribution of SNP numbers for all samples were plotted using ggplot2 (v3.3.2)^30^ in R.

We then used BCFtools to detect the ROHs with parameters: 1) fix allele frequency to 0.4; 2) minimum SNP number of 10 for each ROH segment; and 3) default HMM Options (-a 6.7e-8 -H 5e-9 -V 1e-10). ROH segments with long lengths (> 1 Mb) were shown in a circos (v0.69-8)^31^ plot. The inbreeding coefficient for each sample was estimated based on the ratio of the total length of ROHs to the total length of the autosomal genome (FROH). The proportion of ROH segments with different lengths (>0.5 Mb, >1 Mb, >2Mb) were also calculated. We estimated the offspring had the highest FROH (paternal = 6.96%, mother = 19.02%, F1 = 25.78%).

## Evaluation of assembly quality in ROH regions

We investigated whether there is any bias of assembly quality between ROH and outside of ROH. Here we used the error in k-mer analysis generated by Merqury^32^ (<https://github.com/marbl/merqury>) to evaluate the assembly quality. Upstream and downstream of each ROH of the same length were extracted to compare, and the number of k-mer errors within each dataset were counted. The analysis shows that there was no significant difference with the number of k-mer errors between the ROH and their surrounding regions (two-sided t-test). We also used the number of gaps to assess the assembly quality. The analysis also showed that there is no difference with the number of gaps in the ROH and the surrounding regions. Collectively, the evidence indicates that the assembly quality between ROH and adjacent regions are equally good.

##

## Statistics of enrichment analysis of LINE/L1 and Alu elements with structural variations

## We found that 300 bp long indels enriched with Alu repeats by Chi-squared test. We retrieved 1,632 indels with length 300 bp (length interval: 250~350 bp), 834 of which were annotated as Alu repeat (over 80% of indel length). In the whole genome, we can generate 8,829,881 300 bp long windows (step=300 bp), with about 405,615 windows annotated as Alu repeat. Then Chi-squared test was conducted by following command in R (version 3.5.2):

chisq.test(matrix(c(1632, 834, 8829881, 405615), nrow=2, ncol=2)

X-squared = 5068.9, df = 1, p-value < 2.2e-16

##

We performed statistical tests to estimate the correlation between repetitive elements and translocations or inversions. We calculated the repetitive element rate of the translocation/inversion plus its flanking N bp (where N is the length of the translocation), and compared them with the whole genome background level (mu) using one-sided t-test:

t.test(translocation$rate, alternative = "greater", mu= 0.44427)

t = -1.0498, df = 57, p-value = 0.5812

t.test(inversion$rate, alternative = "greater", mu= 0.44427)

t = 0.89902, df = 26, p-value = 0.1461

To further investigate whether some specific repetitive elements have higher density around translocation, we also did statistics for LINE, LINE/L1, and SINE/Alu in translocation associated regions. These repeat elements were selected as they are some of the highest percentages among all types of repeats. Similarly, one-sided t-tests were performed on these three datasets with the corresponding whole genome backgrounds:

LINE:

t.test(translocation$rate, alternative = "greater", mu= 0.197994)

t = -2.0047, df = 57, p-value = 0.9751

t.test(inversion$rate, alternative = "greater", mu= 0.197994)

t = 1.859, df = 26, p-value = 0.03719

LINE/L1:

t.test(translocation$rate, alternative = "greater", mu= 0.180544)

t = -1.861, df = 57, p-value = 0.9661

t.test(inversion$rate, alternative = "greater", mu= 0.180544)

t = 1.8545, df = 26, p-value = 0.03752

SINE/Alu:

t.test(translocation$rate, alternative = "greater", mu= 0.118335)

t = 1.3454, df = 57, p-value = 0.09191

t.test(inversion$rate, alternative = "greater", mu= 0.118335)

t = 0.23978, df = 26, p-value = 0.4062

In conclusion, we found there is a higher density of LINE and LINE/L1 around the inversions, but no repeat element was enriched with translocations.

##

## Additional filters to reduce false-positive in *de novo* mutation detection

Various filters were applied at the potential Mendelian violation to reduce false-positive calls, especially at chimerism sites. The first filter was on the site and applied as follows: QD < 2.0, FS > 20.0, MQ < 40.0, MQRankSum < -2.0, MQRankSum > 4.0, ReadPosRankSum < -3.0, ReadPosRankSum > 3.0, SOR > 3.0. The second set of filters were applied to each individual:

- a depth filter DP < 0.5 × individual average depth and DP > 2 × individual average (average depth offspring: 40.5X, father: 72.6X, and mother: 76.9X). This filter would remove any high coverage caused by mapping problems and low coverage sites that are more sensitive to false-positive calls.
- a genotype quality filter GQ < 99 for at least one individual. This filter was set particularly high (generally GQ < 40 to 60 in other *de novo* studies) to avoid a maximum of chimerism sites in the father, as those sites tend to have a lower genotype quality due to the presence of multiple alleles.
- an alternative allele filter AD > 0 allowed in the homozygous parents. Again, this filter was set stringent with no alternative allele allowed in any parents as most of the chimerism sites would present at least a few alternative alleles in the variant calling files.
- an allelic balance filter AB < 0.3 and AB > 0.7 on the reads supporting the alternative allele in the heterozygous offspring. This filter would remove any potential sequencing errors in the offspring or chimerism cells as those should present a lower allelic balance (~10-20 %) than the real de novo mutations (~50 %).

On the maternal side, this allowed us to detect:

- 181 candidates for maternal *de novo* mutations as sites where the parents are homozygous for the reference (0/0) and the offspring is heterozygous (0/1) when mapped to the paternal genome.
- 21 candidates for maternal *de novo* mutations as sites where the parents are homozygous for the alternative (1/1) and the offspring is heterozygous (0/1) when mapped to the maternal genome.

This resulted in **3 maternal *de novo* candidate mutations** when comparing the overlapping candidate of the 2 datasets. They were all validated by PCR amplification.

And on the paternal side:

- 192 candidates for paternal *de novo* mutations as sites where the parents are homozygous for the reference (0/0) and the offspring is heterozygous (0/1) when mapped to the maternal genome.
- 27 candidates for paternal *de novo* mutations as sites where the parents are homozygous for the alternative (1/1) and the offspring is heterozygous (0/1) when mapped to the paternal genome.

This resulted in 15 *de novo* candidate mutations when comparing the 2 datasets. Among which, 3 were removed due to polishing or sequencing errors, 5 were removed due to unvalidated PCR validation and 1 failed to be amplified. Thus, we retained 6 paternal *de novo* candidate mutations.

## PAR identification

All identified X-linked sequences were aligned to all Y-linked sequences with MUMMER and the synteny alignment was merged and called by SyRi (v1.0)^33^. The PAR was identified according to the longest continuous synteny alignment between X-linked and Y-linked sequences.

## X-Y divergence estimation

X-Y divergence statistics and the total numbers of aligned sites in CDS regions of genes on marmoset sex chromosomes are reported in **Supplementary Table 27**. The number of sites in CDS alignments of genes in the marmoset-speciﬁc sex-determining region (MSSDR) is much lower when considering only CDS alignments compared to the whole gene Cactus alignment (**Supplementary Table 27 & 28**). The whole gene alignments of X-Y homologues were extracted from the alignment of the ﬁrst 5 Mb between the X and Y chromosomes using the Cactus aligner with default settings^34^. The resulting alignment blocks were retained for further analysis if they uniquely aligned between the chromosomes. We concatenated blocks that were synteneous and separated with less than a 100 base pairs. Lastly, we ﬁltered out the concatenated blocks that were < 100 bp in length.

As divergence estimates vary widely for the CDS alignments (**Supplementary Table 27**), they remain relatively stable for the Cactus alignment due to a substantially higher number of aligned sites (**Supplementary Table 27 & 28**). It is therefore preferable to use the Cactus alignment for estimating the age of the MSSDR. We employ two methods in estimating the time of MSSDR formation, $t$. The ﬁrst method is based on accumulated divergence between X and Y regions since MSSDR formation, which can be expressed as

$d_{XY}=(\mu_{X}+\mu_{Y})t$,

where $\mu_{X}$is the X-speciﬁc per position per year (PPPY) mutation rate and $\mu_{Y}$is Y-speciﬁc per base per year mutation rate of the new stratum. Under the assumption that males contribute $\alpha$ more mutations than females per year, denoting $\mu_{m}$and $\mu_{f}$as male and female-speciﬁc mutation rates, respectively, and given that X and Y chromosomes spend different amounts of time in males and females, we can write down the following expressions

$\mu_{Y}=\mu_{m}=\alpha\mu_{f}$,

$$\mu_{X}=\frac{2}{3}\mu_{f}+\frac{1}{3}\mu_{m}=\frac{2}{3}\mu_{f}+\frac{1}{3}\alpha\mu_{f}=\frac{2+\alpha}{3}\mu_{f}$$

$$d_{XY}=\frac{2(1+2\alpha)}{3}\mu_{f}t$$

Most estimates of mutation rates are usually based on autosomal data. To express $\mu_{f}$ as a function of the autosomal rate $\mu_{A}$we note that

$\mu_{A}=\frac{1}{2}\mu_{f}+\frac{1}{2}\mu_{m}=\frac{(1+\alpha)}{2}\mu_{f}$, and

$$\mu_{f}=\frac{2}{(1+\alpha)}\mu_{A}t$$

Finally, $d_{XY}$ can be expressed as

$d_{XY}=\frac{4(1+2\alpha)}{3(1+\alpha)}\mu_{A}t$,

And the age of the MSSDR as

$$t=\frac{3(1+\alpha)}{4(1+2\alpha)}\frac{d_{XY}}{\mu_{A}} (1)$$

The second method of estimating $t$ is based on calculating the difference between MSSDR divergence of X ($d_{XX_{H}}$) and Y ($d_{YX_{H}}$) sequences with respect to their human homologues. Namely,

$d_{XX_{H}}=\mu_{P,H}s+\mu_{P,M}(s-t)+\mu_{X}t$, and

$d_{YX_{H}}=\mu_{P,H}s+\mu_{P,H}(s-t)+\mu_{Y}t$,

where $s$ is the divergence time between human and marmoset, and $\mu_{P,H}$ and $\mu_{P,M}$ are PAR-speciﬁc divergence rates for the human and marmoset PAR, respectively. When taking the difference $d_{XX_{H}}-d_{YX_{H}}$ , PAR-speciﬁc divergence rates cancel out and we are left with

$$d_{XX_{H}}-d_{YX_{H}}=(\mu_{X}-\mu_{Y})t$$

Expressing the mutation rates as a function of $\mu_{A}$ and solving for $t$ yields

$$t=\frac{3(1+\alpha)}{4(1-\alpha)}\frac{(d_{XX_{H}}-d_{YX_{H}})}{\mu_{A}} (2)$$

The parameters $\mu_{A}$ and $\alpha$ are necessary to estimate the time of MSSDR formation using eqs. (1) and (2). The per site per generation mutation rate for the marmoset, estimated within this study, is 0.43 × 10^−8^ with a male mutation bias $\alpha=2$. This estimate can be transformed into a yearly estimate by dividing with the average age of parents at conception. Given the date of birth for parents and offspring (**Supplementary Table 29**), and the fact that the gestation period of the common marmoset is approximately 150 days, the average age of parents at conception is approximately 2.8 years, which yields $\mu_{A}$= 1.53 × 10^−9^ PPPY (**Supplementary Table 30**). Generally, the estimates of $\mu_{A}$vary across primates, with a slowdown of mutation rates in humans and great apes, compared to other primates. The marmoset $\mu_{A}$estimate is most similar to other non-hominid primates, such as the African green monkey and the owl monkey, which are the only other new world monkey species for which a $\mu_{A}$ estimate is available (**Supplementary Table 30**). Therefore, to obtain a range of age estimates for the MSSDR, we assume a possible range of marmoset $\mu_{A}$ and $\alpha$ values based on the similar estimates for the African green monkey and the owl monkey. Using values from **Supplementary Table 28**, we calculate $d_{XY}$ as the diﬀerence 0.0367−0.0095 = 0.0272 to account for the accumulated divergence prior to MSSDR formation, and $d_{XX_{H}}-d_{YX_{H}}$ as 0.2815 − 0.2879 = −0.0064. The ranges of t estimates obtained using eqs. (1) and (2) and diﬀerent $\mu_{A}$and $\alpha$ values largely overlap and span the timeframe of 5.23-12.97 Myr ago as the putative time of MSSDR formation (**Supplementary Table 31**).

## Visualization of alignment between X-linked and Y-linked sequences

Marmoset X-linked and Y-linked sequences were softmasked and aligned with LASTZ (v1.04.00)^35^ with parameter set “--step=19 --hspthresh=2200 --inner=2000 --ydrop=3400 --gappedthresh=10000 --format=axt” and a score matrix for distantly-related species comparisons. Only alignments larger than 500bp were kept and identity was calculated. Alignments were further classified into three groups (PAR, MSSDR, and ancestral SDR) according to the position on the X for sequence identity comparison. Because *P2RY8X*, *AKAP17AX,* and *ZBEDY* were missing in mCalJac1, we manually added them back in the plot as well as the following strata analysis.

## Heterozygosity rate calculation

NGS reads of chimpanzee (ERP002376) and marmoset (from mCalJac2) were aligned to the respective genomes with BWA MEM; SNP calling was performed using bcftools (v1.9-102-g958180e) mpileup and call. The human SNV data of HG00096 was obtained from <https://www.internationalgenome.org/> to compare the SNP heterozygosity of these three species. Average heterozygosity was calculated within the PAR and autosomes. We also calculated heterozygosity rate in a non-overlapping 2 kb window in the PAR for visualization.

## Gametologue identification and pairwise dS calculation

All annotated Y-linked protein coding genes were translated into proteins, aligned to X-linked genes with BLASTP (v2.2.26), and the best hit was kept. We further included *AKAP17AX*, *ZBEDY*, and *ARSHX* from GCA_009663435.2 and *P2RY8X* from GCF_000004665.1 as they were missing or were chimeric in the mCalJac1 assembly. Candidate gametologue pairs were confirmed if both the maternal and paternal sequences were mapped to the same gene in the NCBI NR database. Translated genes were aligned by PRANK (v150803)^36^, filtered by Gblocks (v0.91b)^37^, and converted back into CDS alignment. dS was calculated by codeml in PAML (v4.8)^38^ with “runmode=-2”.

## Expression analysis

We generated a new assembly by merging the maternal assembly with all the Y-linked sequences from the paternal assembly, and further masked PAR on Y-linked sequences into Ns. In addition, *AKAP17AX* and *ZBEDY* from GCA_009663435.2, *P2RY8X* from GCF_000004665.1, and their flanking 2 kb sequences were also extracted and merged into the new assembly. RNA-seq reads of marmoset (SRP029760) were mapped to the assembly by HISTA2 (v2.0.5)^39^. Only uniquely mapped reads were used in the RPKM calculation and RPKMs were normalized with DESeq (v1.9.12)^40^ to generate an expression matrix. Median PRKM values were calculated for each tissue of each sex. The tissue specificity index TAU was calculated according to the definition from Yanai et al. 2005^41^. For tissues that were available in both sexes, we computed each X-linked gene’s median RPKM, and computed its F/M RPKM ratio (requiring RPKM in both sex ≥ 1) to determine dosage compensation status. Two-sided Wilcoxon rank-sum test was used to test if there is a significantly different F/M RPKM ratio among genes in PAR, MSSDR, and ancestral SDR.

## Gene phylogenetic analysis

To construct the phylogenetic tree, CDS sequences were first translated into protein, aligned by PRANK, and converted back into CDS alignment. The phylogenetic tree was constructed with RaxML (v8.2.9)^42^ with parameter “-f a -m GTRGAMMA -p 12345 -x 12345 -# 100”. Moreover, in the phylogeny construction of *THOC2X-Y* and *ARSHX-Y*, the chicken or platypus orthologous sequence was specified as the outgroup of the tree in RAxML run.

## Ampliconic gene identification

To detect ampliconic regions, we divided the chromosome assemblies in non-overlapping windows of 500 kb, then we performed a reciprocal blast within each window. We removed hits below 98% identity and below 600bp as well as hits that were overlapping on more than 80% of their length. We then grouped hits corresponding to the same region when they harbored more than two copies. We identiﬁed ampliconic gene content based on both a blast search against human X-linked genes and on the annotation. We conﬁrmed the copy number of such genes by performing a blast search of those identiﬁed genes against both the whole chromosome assembly and the previously identiﬁed ampliconic units. We reproduced this protocol on the human X assembly (hg38) to compare the ampliconic gene content as well as the duplication pattern for common ampliconic genes. Those results were broadly consistent with previous descriptions of human X-linked ampliconic genes^43^. We compared the duplication pattern for common X-linked ampliconic genes by performing a blast search of the marmoset versus human sex regions. We then used the R package genoPlotR (v0.8.9)^44^ to plot the hits (**Supplementary Fig. 24**). Hits are colored in red (if same sense) or in blue (if anti-sense), the intensity of the color depends on the percentage of identity of the blast hits. For four of the six genes, the pattern of duplication is highly similar, strongly suggesting that the duplications originate from single events in the ancestor of human and marmoset. For the regions containing *NXF2* and *CXorf49*, however, the synteny between the two species is less conserved, and the *NXF2* genes copies are not duplicated in the same sense. This makes the independent or common origin of the duplications harder to assess.

We analyzed marmoset ISOseq reads that were mapped at the coordinates of ampliconic units to identify ampliconic genes that are speciﬁcally expressed in testes. Additionally, we performed a BLAST search of all ISOseq reads that mapped on the sex chromosomes against the ampliconic units. We also utilized RNA-seq data to confirm the testes-specific expression pattern. For human, we recovered expression information (that is, if ampliconic genes were speciﬁcally or over-expressed in testes) based on the GeneCard database (https://www.genecards.org/, consulted on 27/04/2020).

## X chromosome evolution reconstruction

X-linked sequences of treeshrew (TS_2.0), marmoset (mCalJac1), green monkey (Chlorocebus_sabeus 1.1), rhesus (rheMacS), orangutan (Susie_PABv2), gorilla (Susie3), and chimpanzee (Clint_PTRv2) were obtained from NCBI, softmasked, and aligned to human (hg38) X chromosome by LASTZ with parameter set “--step=19 --hspthresh=2200 --inner=2000 --ydrop=3400 --gappedthresh=10000 --format=axt” and a score matrix was generated for distantly-related species comparison. Conserved blocks, which were available in all eight species, and larger than 300 kb, were extracted from net and chain files and were then used as the input of MGRA2 (v2.2)^45^ for reconstruction.

## Bioinformatic confirmation about the loss of *HSFY* and *VCY*

Genewise was performed on the paternal PacBio corrected reads produced by TrioCanu to annotated *HSFY* and *VCY* with queries collected from Uniprot (HSFY: Q96LI6, VCY: O14598, O14599). We did not annotate either *HSFY* or *VCY* in the paternal PacBio corrected reads, suggesting that these two genes are absent in the sequenced DNA.

## Identification of orthologous genes for positive selection

First, we aligned protein sequences of marmoset and another species (cow， mice, macaque, and human were downloaded from Ensembl release 98, chimpanzee (Clint_PTRv2), treeshrew (TS_2.0), white-faced capuchin (Cebus_imitator-1.0), and black-capped squirrel monkey (SaiBol1.0)) to each other by BLASTP with a E-value cut-off of 1e-5, and combined local alignments with the SOLAR program (download from http://treesoft.svn.sourceforge.net/viewrc/treesoft/). We filtered out those candidate orthologs with homologous block lengths of < 30% of length of the longest protein. In addition, we filtered out candidate orthologs with identity < 50%. Finally, we only kept the orthologous groups in which no species were absent. After filtering, we identified reciprocal best hit (RBH) orthologs in all aligned gene pairs by sorting pairs of score, alignment rate, and identity in turn.

**Analysis of genes related to human brain development in marmosets**

We first performed BLASTP searches of proteins between marmoset and human (Evalue 1e-5); only the best hit of each marmoset protein was kept. Genes related to brain development or neurological diseases were obtained according to the human GO and HPO database (<https://hpo.jax.org/>). We also performed gene family clustering with protein sequences from six primate species (including human (hg38), marmoset (mCalJac1), chimpanzee (Clint_PTRv2), rhesus macaque (rheMacS), Ma’s night monkey (Anan_2.0), and Chinese tree shrew (TS_2.0)) by orthoMCL (v1.4)^46^. Combining the result from pairwise BLASTP, we manually corrected some gene family clustering results, constructed phylogenetic trees with TreeBest (v1.9.2, nj method)^47^ to effectively handle large gene families, and manually examined all the topologies to exclude false positives by filtering out genes which cluster with the other human gene paralogue that is not the search target in the gene family. For the one-to-one orthologues, translated genes were aligned by PRANK, filtered by Gblocks, and converted back into a CDS alignment. Pairwise dN/dS was calculated by codeml in PAML with “runmode=-2”.

We found *ASPA* was duplicated twice in the marmoset (**Supplementary Fig. 33**). A mutation of this gene in humans causes Canavan disease^48^, a neurodegenerative disorder characterized by the spongy degeneration of the white matter of the brain. *ASPA* knockout mice showed severe vacuolation in the white matter and gray matter of the spinal cord^49^. Additionally, the *ARF1* gene experienced a species-specific duplication in marmosets. Human patients with mutations in this gene developed periventricular heterotopia syndrome, where cortical brain cells do not migrate properly during development^50^. We propose the expansion of these genes in the marmoset are candidates for contributing to the higher percentage of grey matter in the primary visual cortex of marmoset than in human^51^.

Additionally, we found that eight human-marmoset orthologues show high pairwise dN/dS (dN/dS > 1, **Supplementary Table 37**), an indication of diversification selection between the two species. The *TIMMDC1* gene has the highest human-marmoset pairwise dN/dS among all investigated genes (**Supplementary Table 37**) and is one of the PSGs detected in the NWMs (**Supplementary Table 35**). It encodes a protein involved in mitochondrial complex I assembly^52^, and patients with a mutated *TIMMDC1* developed numerous diseases including neurological deterioration^53^. Other genes with high dN/dS values between the two species are also necessary for brain functions, including selenoprotein P (*SELENOP*), which is responsible for the transport of selenium and is crucial for brain development^54^.

# **Supplementary Figures**


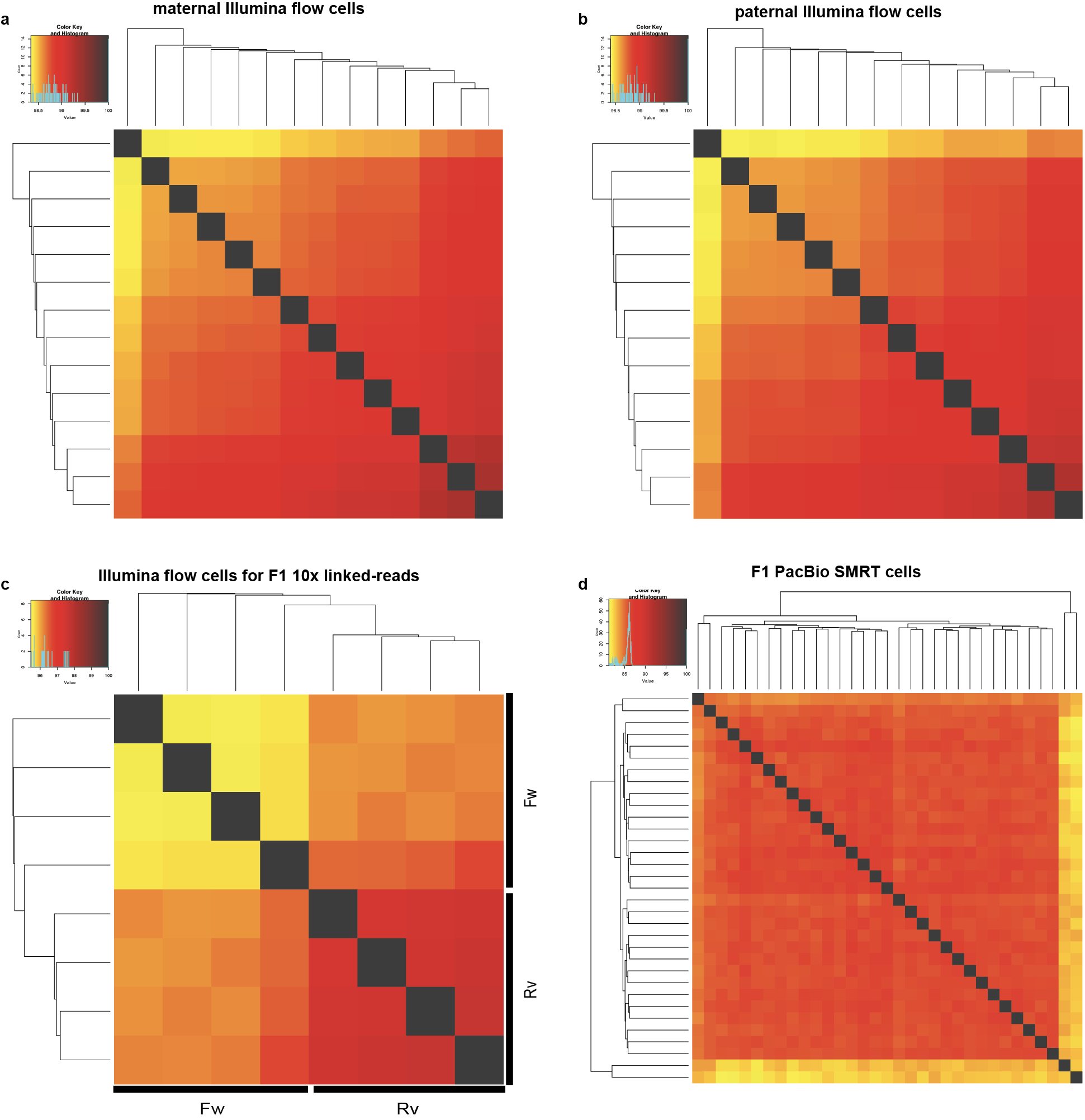


## Supplementary Figure 1. Mash quality control assessment of raw sequencing reads to assure no contamination. Output alignments of each flow or SMRT cell compared against itself and each other cell for a, maternal Illumina, b, paternal Illumina, c, F1 10x-linked reads (forward and reverse) Illumina, and d, F1 PacBio. Trees indicate similarity of alignment between individual cells; shading also indicates similarity - the darker the more similar. No contamination was detected; 3 of the SMRT cells used for F1 PacBio sequencing produced fewer reads compared to the other 30 cells. Dissimilarity between 10x-linked forward and reverse reads standard and expected due to the unique barcodes of Fw reads prior to trimming.

# **
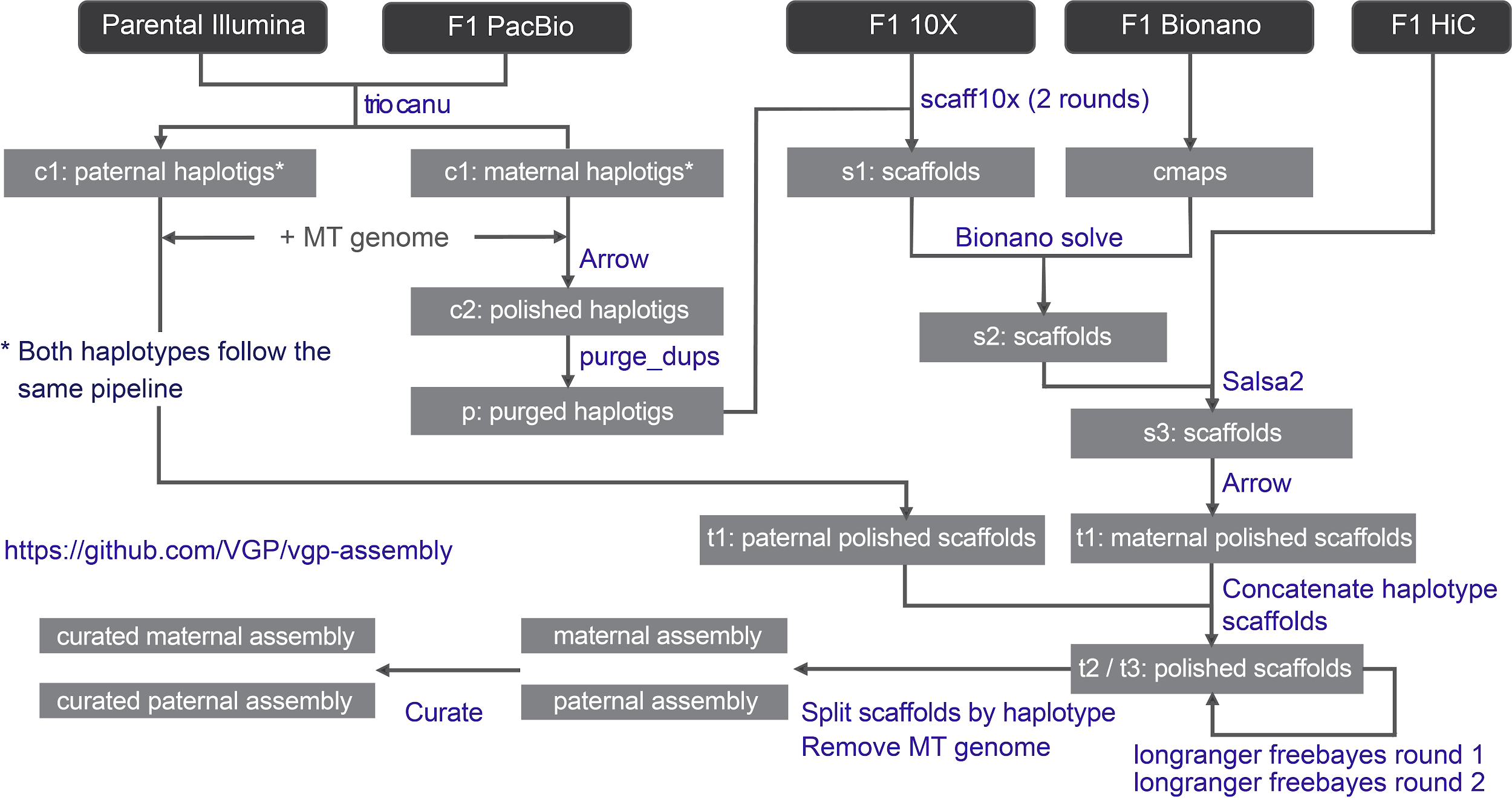
**

## Supplementary Figure 2. Vertebrate Genomes Project-based pipeline for haplotype phased genome assembly of the common marmoset.

##
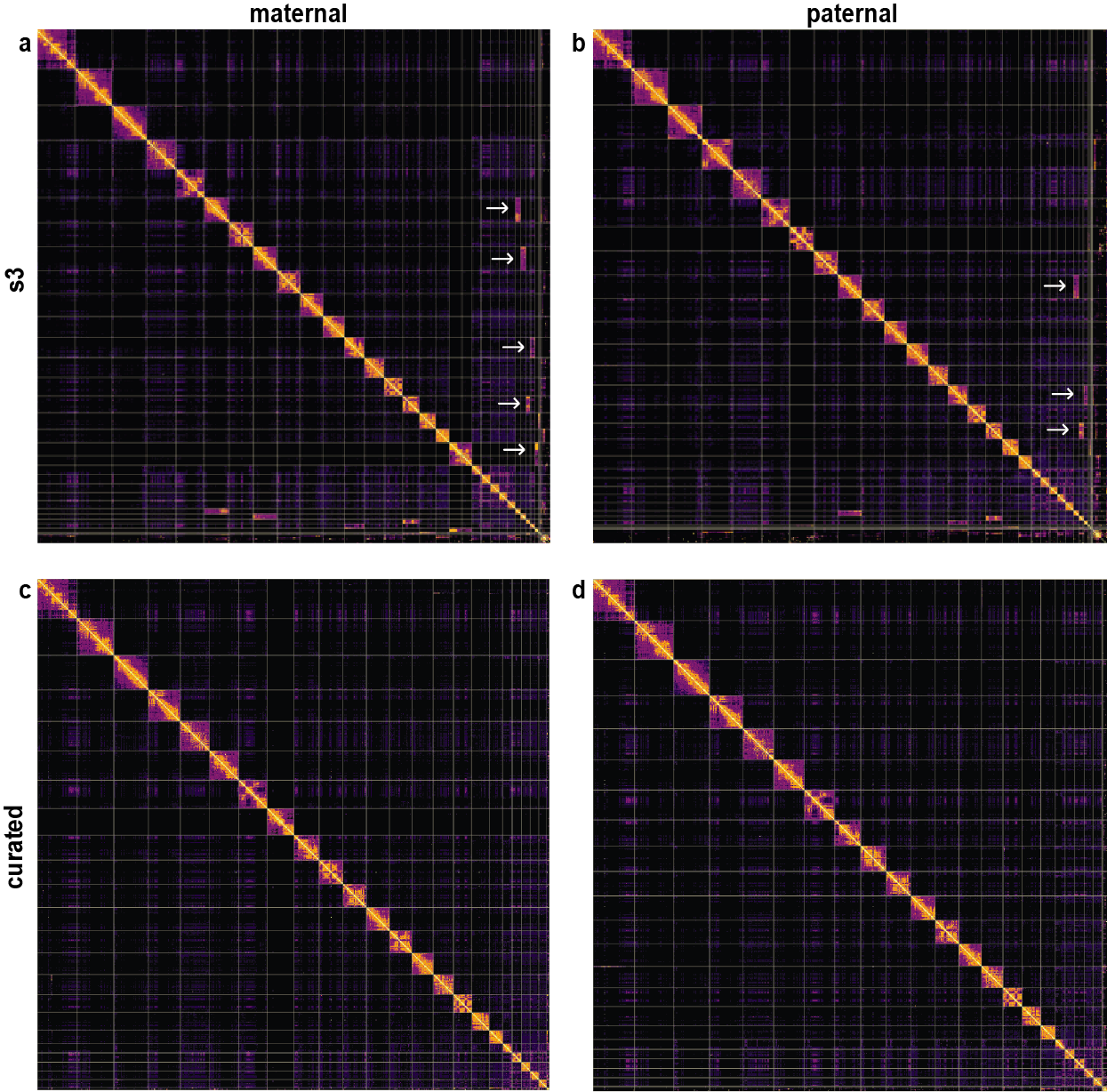


## Supplementary Figure 3. Pretext visualizations of s3 and final curated scaffold contact maps (Arima Hi-C). a, maternal s3 scaffolds, b, paternal s3 scaffolds, c, maternal curated assembly scaffolds, and d, paternal curated assembly scaffolds. Contacts highlighted between each scaffold compared to itself and every other scaffold; intra-molecular (i.e. intra-scaffold) contacts are expected, while extra-scaffold contacts most likely indicate assembly errors. Extra-scaffold contacts (white arrows) resolved from s3 to curated assembly scaffolds.

##
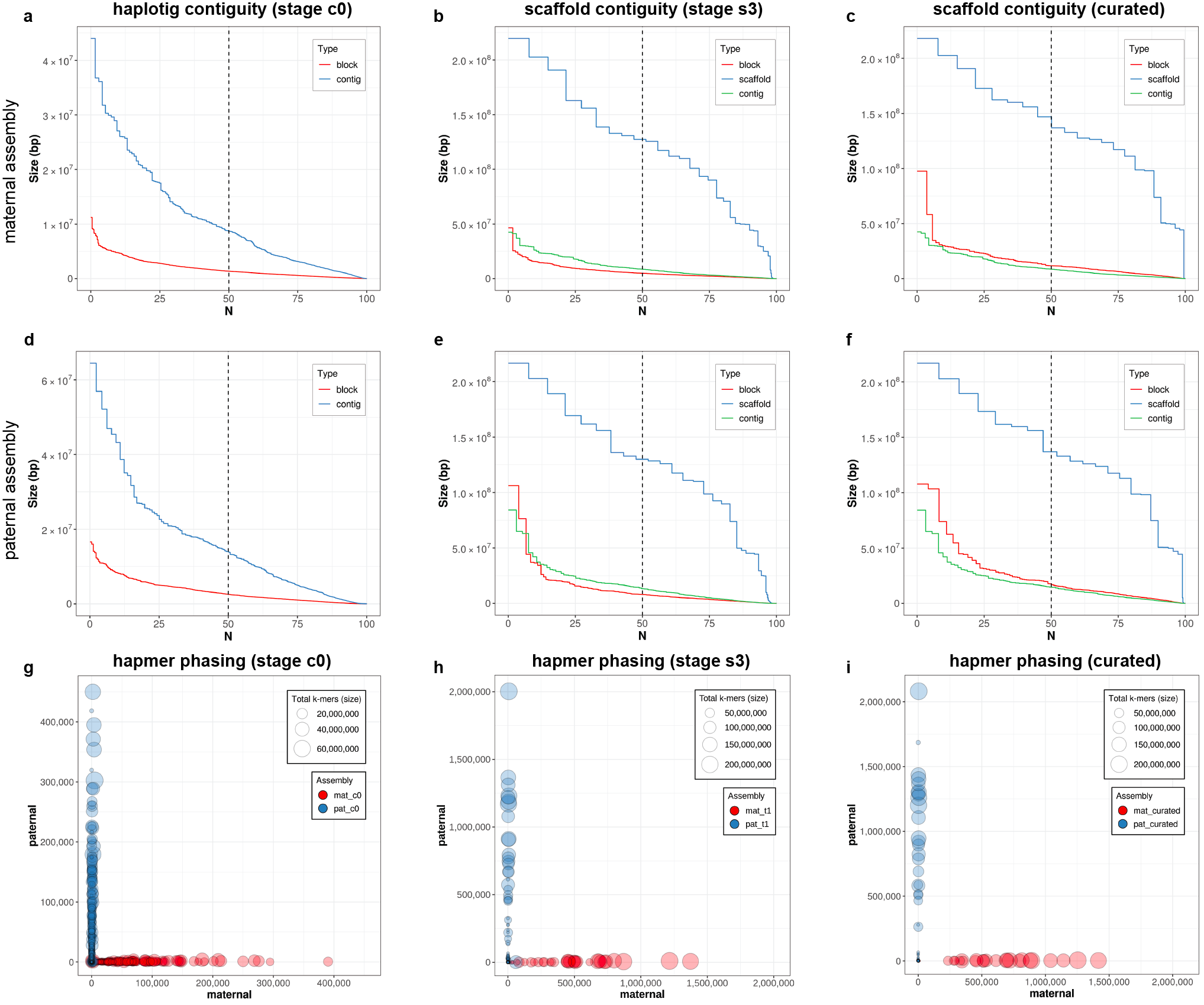
Supplementary Figure 4. Contiguity and phasing through assembly. a-c, Contiguity graphs of the maternal haplotigs, scaffolds, and curated scaffolds, respectively. Dotted line indicates the N50 for contig, block, and/or scaffold length. d-f, Contiguity graphs of the paternal haplotigs, scaffolds, and curated scaffolds, respectively. We see slightly higher contiguity of the paternal assembly at the contig and block level. Scaffolds are similarly contiguous between assemblies. g-i, Blob plots show hapmer phasing of maternal and paternal hapmers among total k-mers counted per individual contigs, scaffolds, and curated scaffolds, respectively. Each blob represents an individual contig or scaffold; size of blob represents the total number of k-mers counted per contig/scaffold; location along axes indicates number of maternal or paternal hapmers counted. Blobs are plotted according to the number of maternal and paternal hapmers counted among the total; we see high phasing of hapmers, with several maternal contigs and scaffolds showing some paternal hapmer representation (not lying directly on the x axis). i, appears in the main text as Extended Data Fig. 2a.

## Supplementary Figure 5. Karyotype image processing for chromosome length estimations. a, original karyotype^11^, b, grey, c, thresholded, and d, thresholded + filled images.
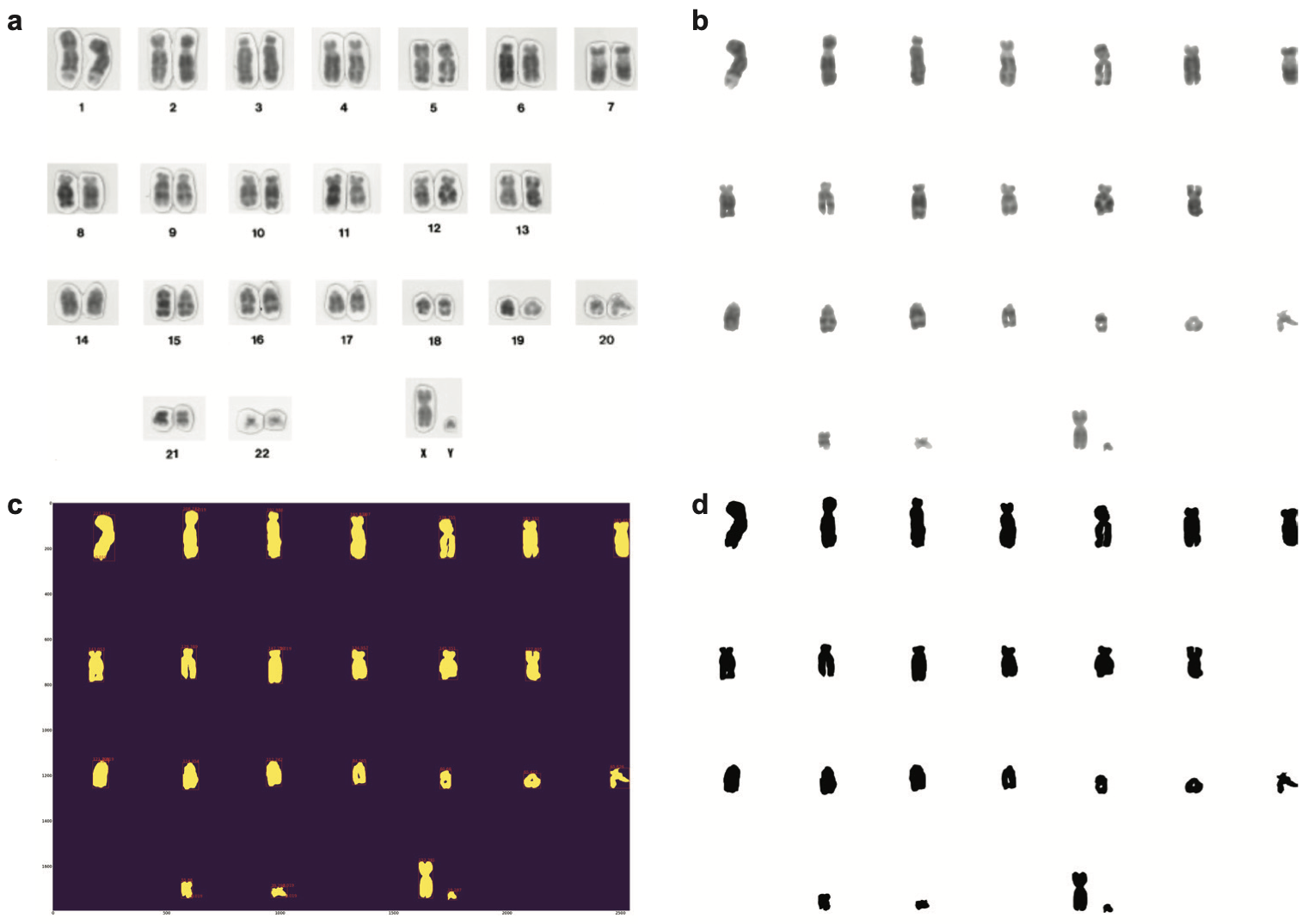


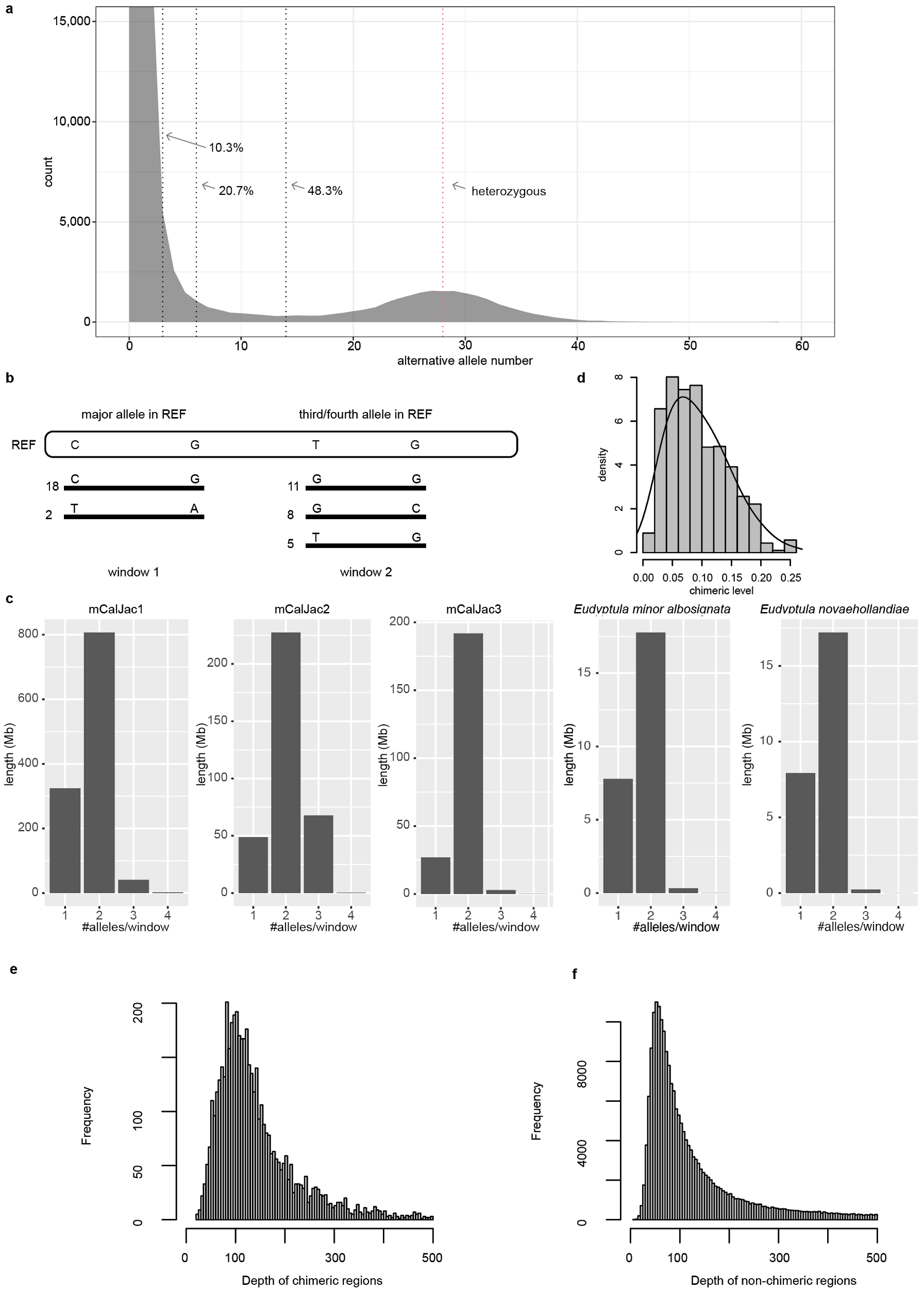


## Supplementary Figure 6. Chimerism estimation in the marmoset assembly. a, Count distribution of alternative alleles in the paternal assembly at bases where alignment of the 10x linked-read set results in exact diploid coverage (58x). As expected, we see a peak at half coverage (29x), indicating heterozygosity from the alternate haplotype (red line). In the presence of chimerism, an additional peak would be expected at a frequency dependent on the extent of the chimeric event. Various chimerism hypotheses are reported by the black dashed lines. These were calculated by dividing the number of alternative alleles observed by the total coverage and multiplying by 2x to account for the fact that the chimeric allele would likely be heterozygous. We do not see a second distinct peak indicating chimerism. At the 6x to 3x coverage lines, we see an increase in counts that fall within the distribution of sequencing errors, therefore significant chimerism was not supported by this analysis. b, Diagram of the allele detection process. Each window spans two SNVs. The number on the left of black bar represents the number of supporting reads for each allele. In this case, window 1 contains two types of alleles and window 2 contains three types of alleles. We can also infer whether our assembled allele is a major or third/fourth allele by ranking the supporting read number of each allele. In this case, the window 2 contains an assembly error from a third/fourth allele. c, Length distribution of different allele categories. The x-axis represents the number of allele types in each window, the y-axis represents the total length of spanned regions. The same analysis was conducted in two non-chimeric diploid penguins (*Eudyptula minor albosignata* and *Eudyptula novaehollandiae*) based on 10X reads. Their barplot patterns are the same as the mother sample, which indicated that the mother has no chimerism. d, Density distribution of chimeric level inferred from windows containing four alleles. e & f. The chimeric regions (N = 4908) (e) tend to have higher depth than the non-chimeric regions (N = 220000) (f), which could be raised by collapsed paralogs.

##
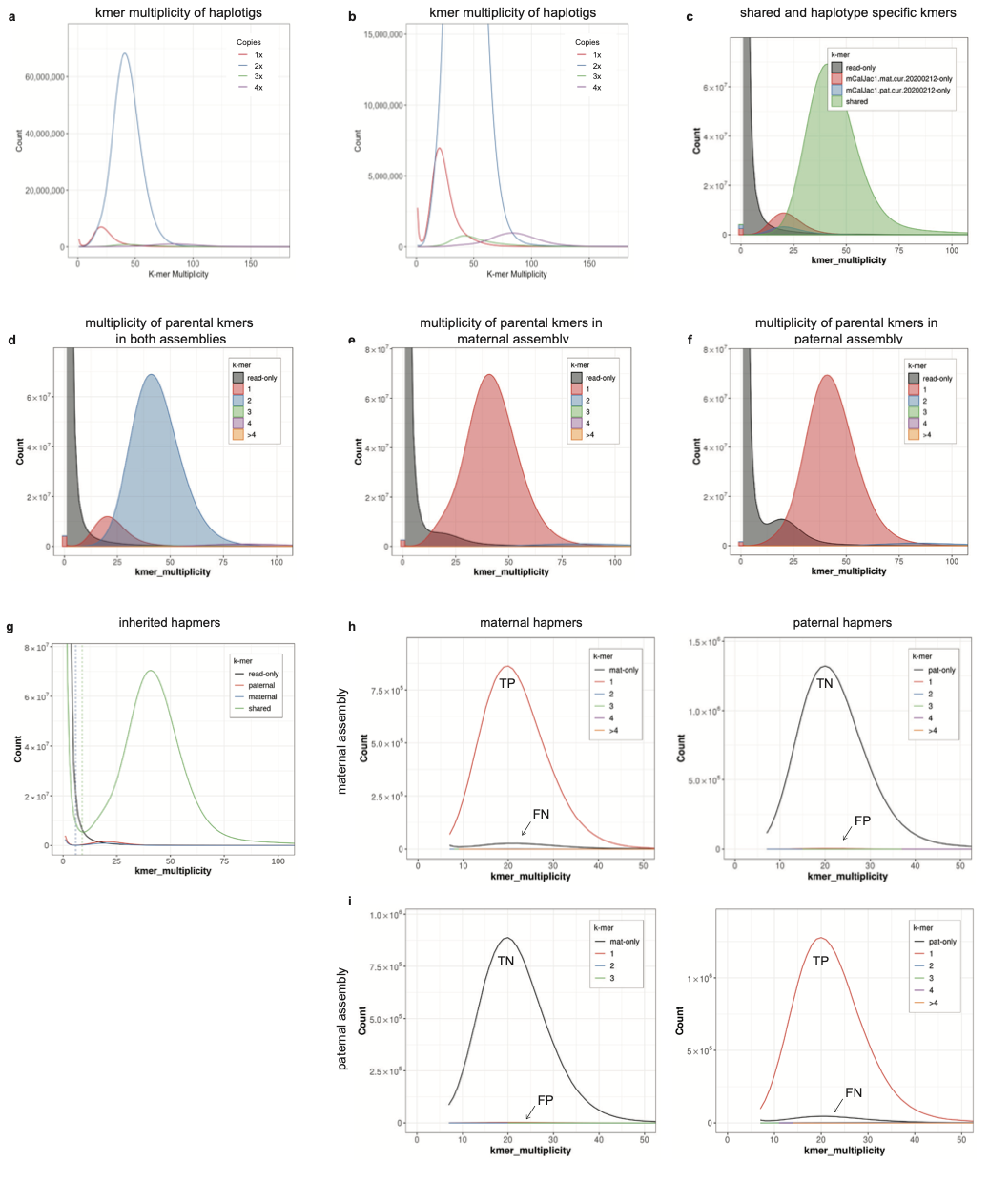
Supplementary Figure 7. Meryl and Merqury k-mer-based analyses. a & b, (zoomed in) Representation of k-mer multiplicity of 21-mers, collected from the F1 10x reads via meryl, colored by the number of times each k-mer is found in all haplotigs (both maternal and paternal) after the first contigging stage (c0). Much of the genome is homozygous; we see the majority of k-mers represented twice at about the average coverage of our 10x data (indicating one representation per haplotype). 1x copies at half that coverage indicates heterozygosity of alleles. 3x at the same coverage as 2x likely indicates artificial duplication, while at 1.5 times higher coverage indicates true duplications. 4x at 2 times higher coverage than average indicates real duplications (either 4x in one or 2x per haplotype). We see low numbers of potential artificial duplications - an estimated 3.338% of all 2,224,721,916 21-mers counted may represent artificial duplications. c, The spectra assembly plot shows the number of shared and unique k-mers per assembly; the majority of 21-mers are shared, while the maternal assembly shows more unique 21-mers than the paternal assembly. We expect this result due to the significantly larger maternal X chromosome compared to the paternal Y. d, The spectra copy number plot shows the final k-mer multiplicity, or overall copy numbers, of the 21-mers found in the parental Illumina reads colored by number of times found in both assemblies. e & f, Further broken down by haplotype, we see the results of the spectra assembly plot mirrored in that the maternal exhibits more 1-copy 21-mers than the paternal assembly. g, Looking at the number of inherited hapmers we see there are more paternal specific k-mers represented. This is expected as the father has its own Y chromosome, whereas k-mers representing X chromosomes in both the mother and father would largely overlap and therefore be removed during building of the haplotype specific k-mer databases. Looking at hapmer representation in the maternal assembly (h) and paternal assembly (i) we see a very high proportion of hapmers were correctly captured in our assemblies (true positive, TP, hapmers identified in the raw data found in the final assembly) with a low number of false negatives (FN, hapmers identified in the raw data not found in the final assembly). We also see a negligible number of hapmers of one haplotype represented in the opposite assembly; practically no false positives (FP) are identified and the k-mers are primarily identified as true negatives (TN).

##

##
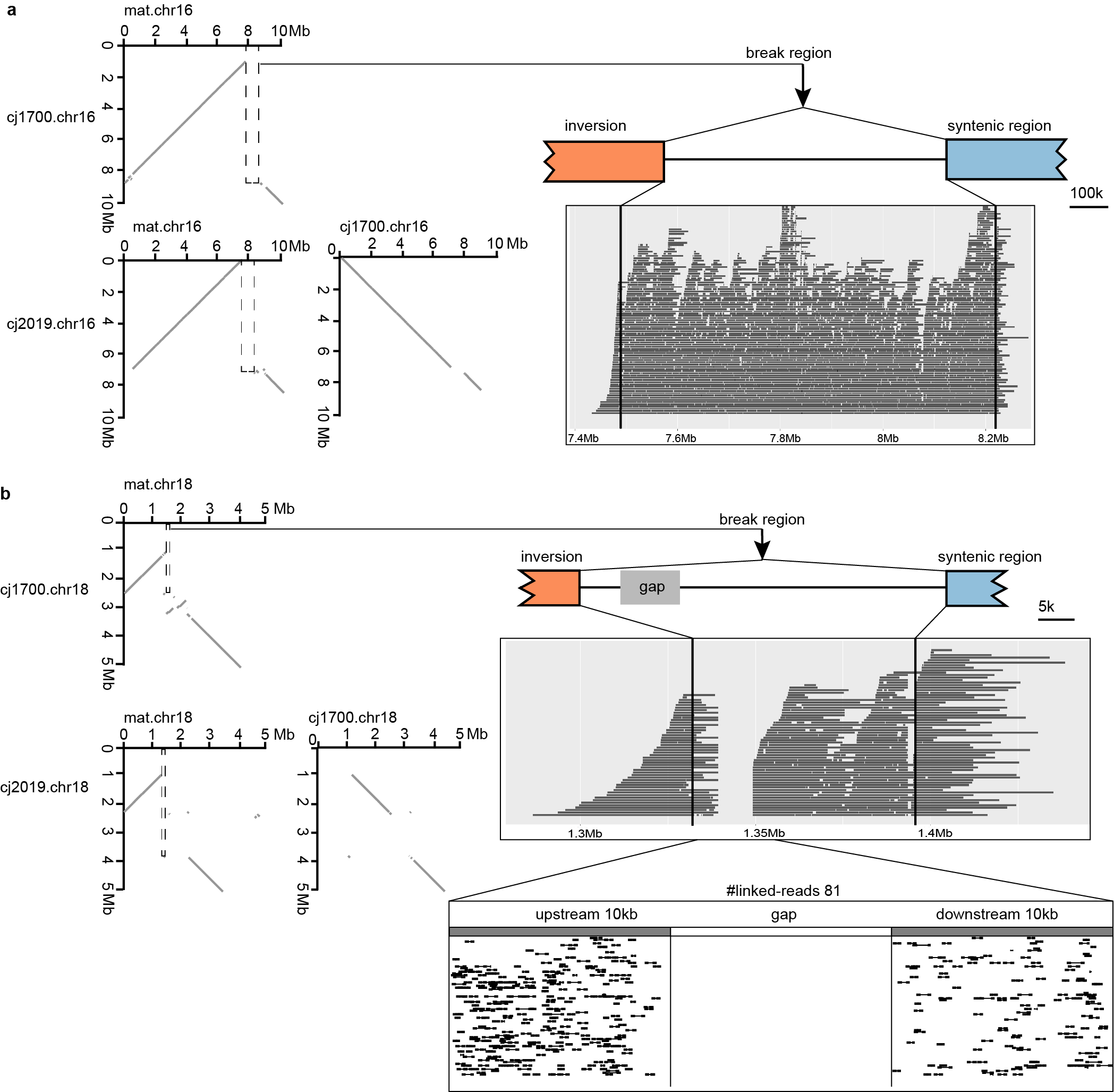


## Supplementary Figure 8. Visualization of two intrachromosomal structural variations in both mCalJac1.mat-cj1700 and mCalJac1.mat-cj2019. a, An intrachromosomal structure variation is found on chr16 in both mCalJac1.mat-cj1700 and mCalJac1.mat-cj2019. PacBio subreads can span the break region, which suggests that mCalJac1.mat assembly is correct. b, An intrachromosomal structure variation is found on chr18 in both mCalJac1.mat-cj1700 and mCalJac1.mat-cj2019. PacBio can span the break region except at the gap, while 81 10X linked-reads can span the gap. These two pieces of evidence suggest that mCalJac1.mat assembly is correct. Each linked read is shown in each line in the 10X panel.

##
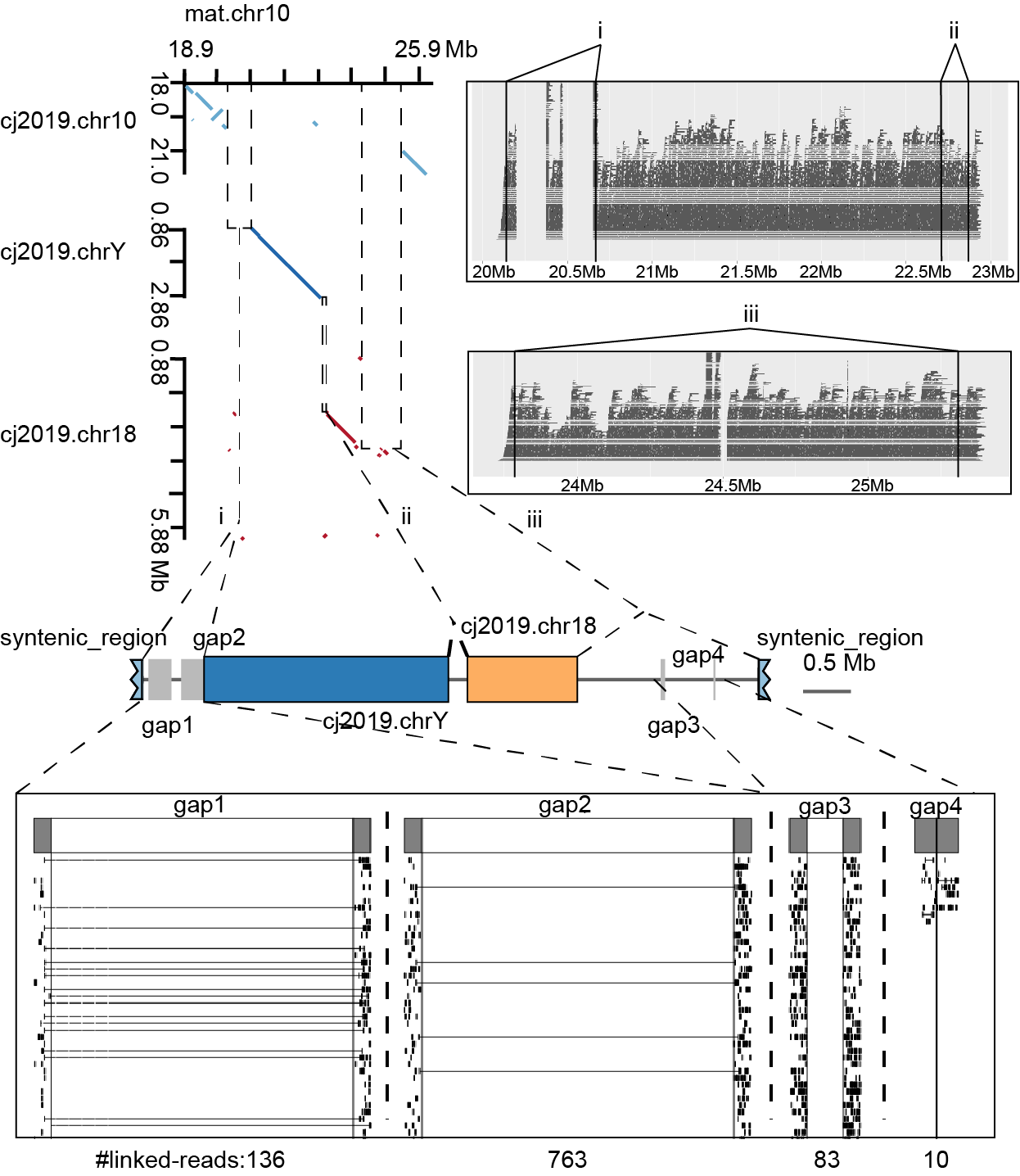


## Supplementary Figure 9. Visualization of an interchromosomal structural variation between mCalJac1.mat and cj2019. There are two other chromosome (chr18 & chrY) segments of cj2019 aligned to mCalJac1.mat chr10. PacBio subreads and 10X linked-reads were used to check these break regions and the structure of mCalJac1.mat chr10 was confirmed to be correct.

##
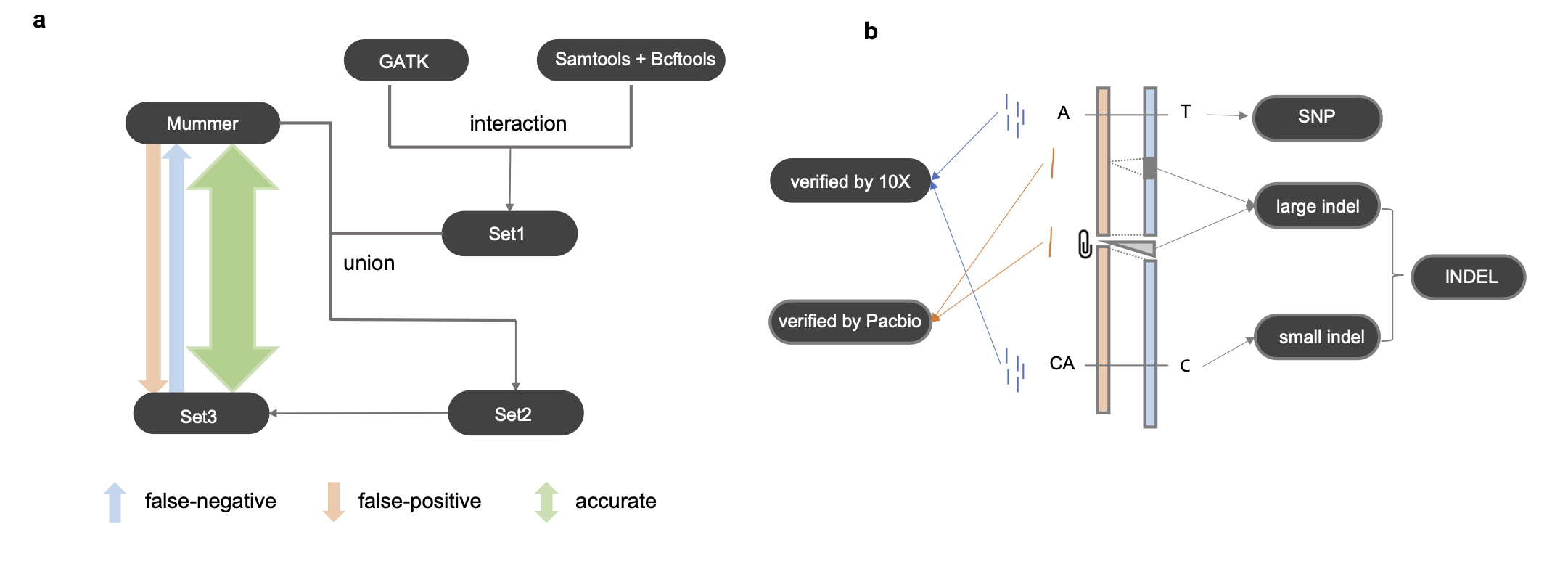


## Supplementary Figure 10. Illustration of the pipeline for sequencing error and polishing error detection, and SNP, INDEL validation. a, Three independent approaches were used to detect SNPs, to generate a benchmark dataset Set3. The accuracy can be calculated by comparing mummer SNP results to Set3. b, The yellow and blue blocks represent the genome alignment between maternal and paternal assemblies. The SNP and small indels in alignment can be verified by 10X reads and PacBio long reads, while large indels can be verified by PacBio reads.


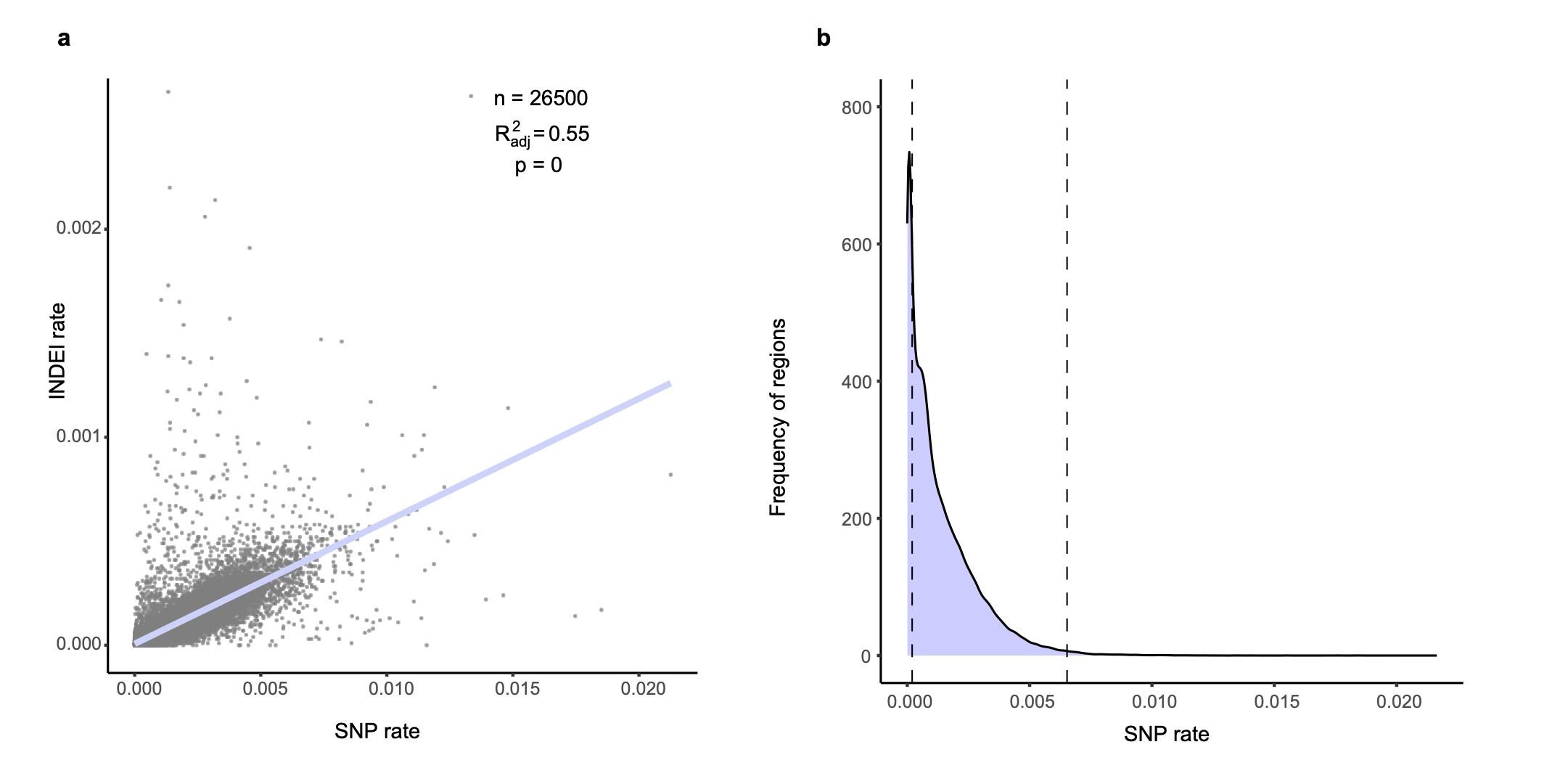


## Supplementary Figure 11. Heterozygosity between the two haplotypes of marmoset. a, The correlation between SNP density and small indel density. A total of 26,500 windows are used in the plot. b, The curve summarizes the distribution of heterozygosity rates for the two haplotypes of marmoset using a window sliding approach (window size=100 kb, step size=20 kb). Two black lines indicate the 1% and 95% cutoff for distribution.


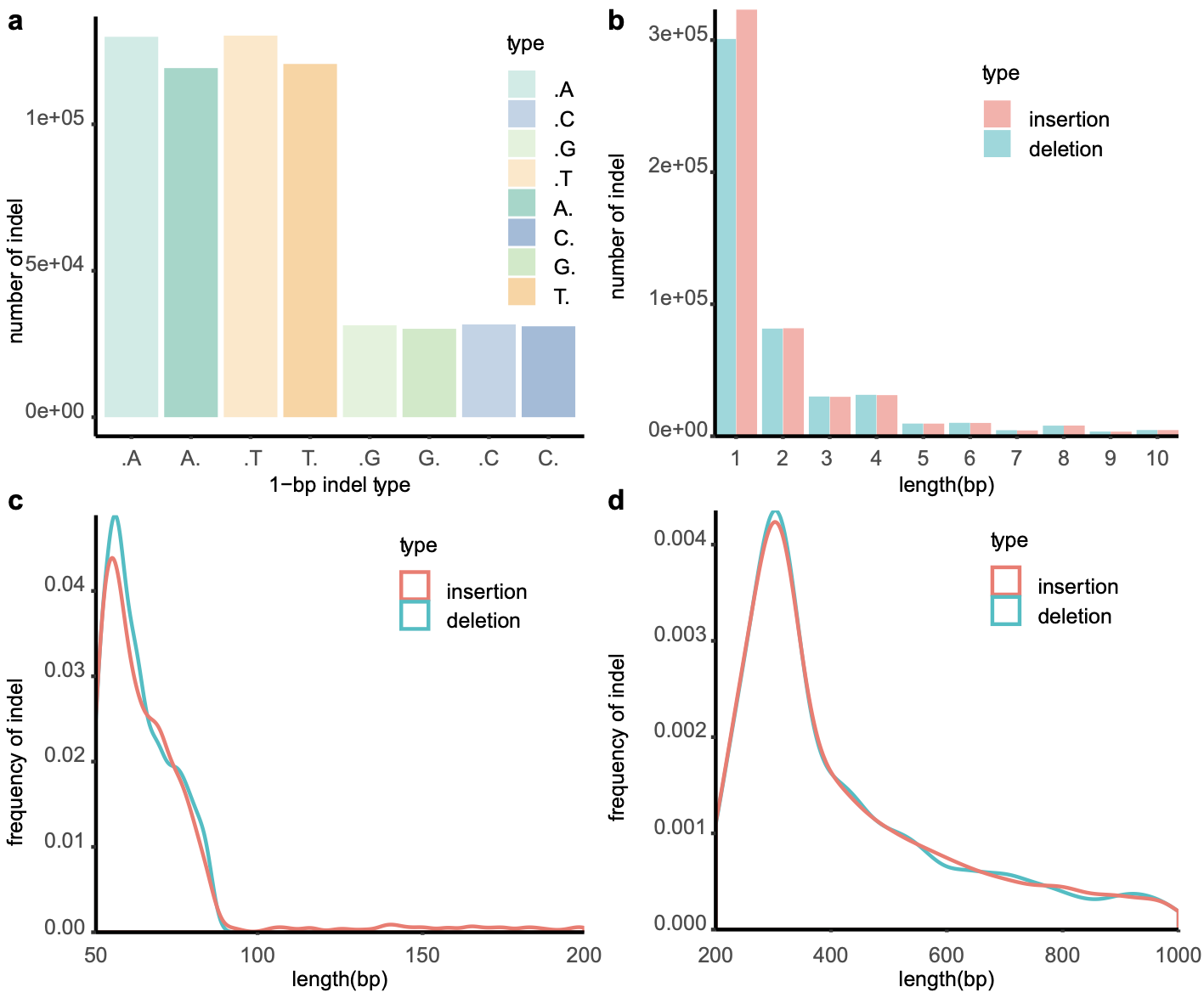


## Supplementary Figure 12. Length distribution of indels. a, The 1-bp indel subtype distribution shows that deletion in paternal assembly is slightly more than that in maternal. For the type meaning, “.A” means none in maternal, and “A.” in paternal (a deletion of maternal or insertion of paternal), etc. b, Length distribution of short indels (<10 bp). c, Length distribution of intermediate sized indels (50 bp~200 bp). Note that there is no bias between insertion and deletion, which is progress compared to previous studies. d, Distribution of long indels (200 bp~1 kb). The peak at ~300 bp results from the enrichment for *Alu* element insertions and deletions.


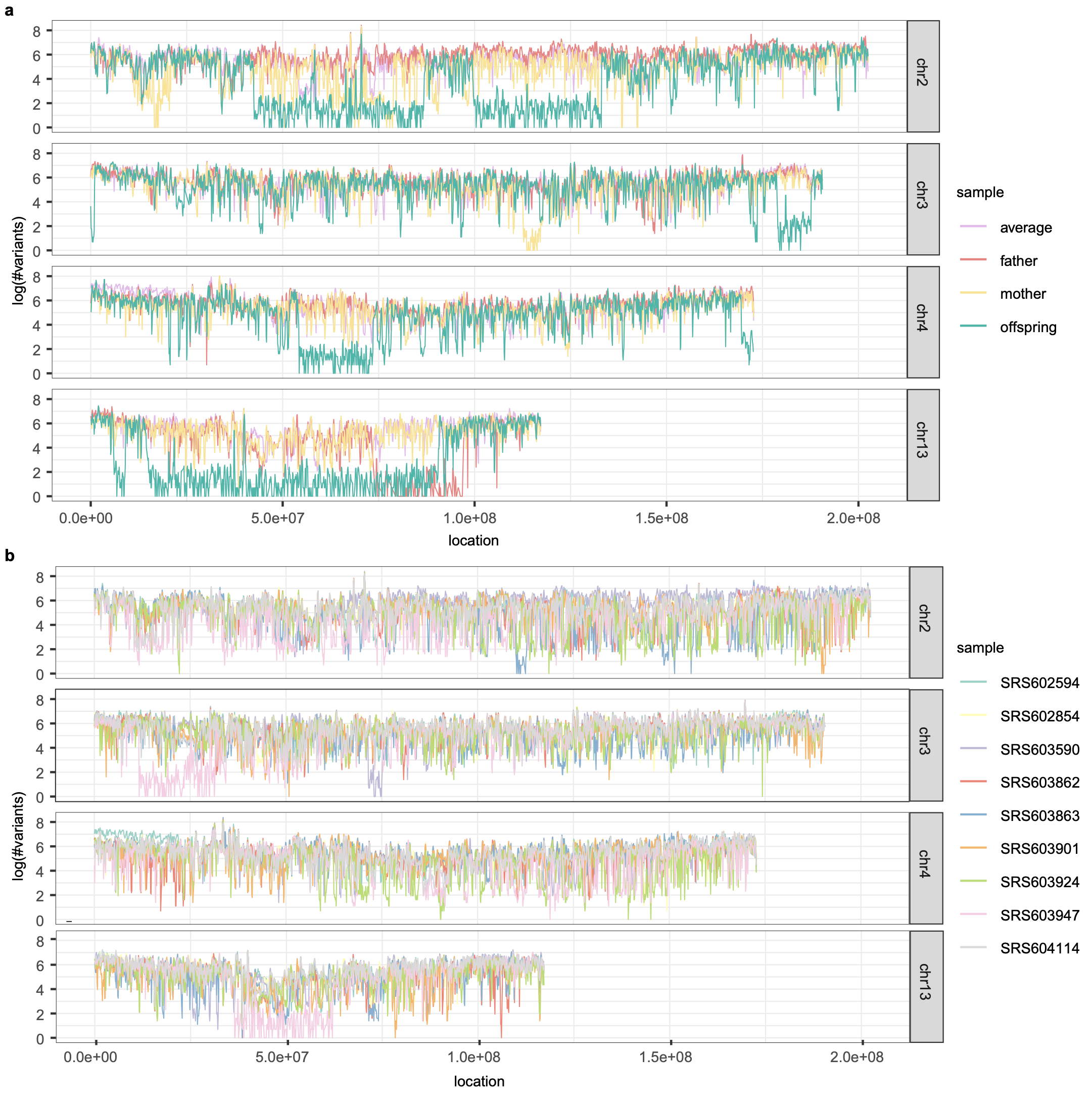


## Supplementary Figure 13. SNP density distribution of 9 published samples and our trio samples on chr2, chr3, chr4, and chr13. a, The F1 animal has very low heterozygosity on chromosome 2, 4, and 13 compared to a background of chromosome 3. Compared to the other nine samples, our trio was significantly higher FROH (17.24% on average). b, The SNP density distribution of 9 samples indicates that these samples have a wide range of heterozygosity. The sample SRS603947 partially shared a large ROH region with the F1 animal on chr13.


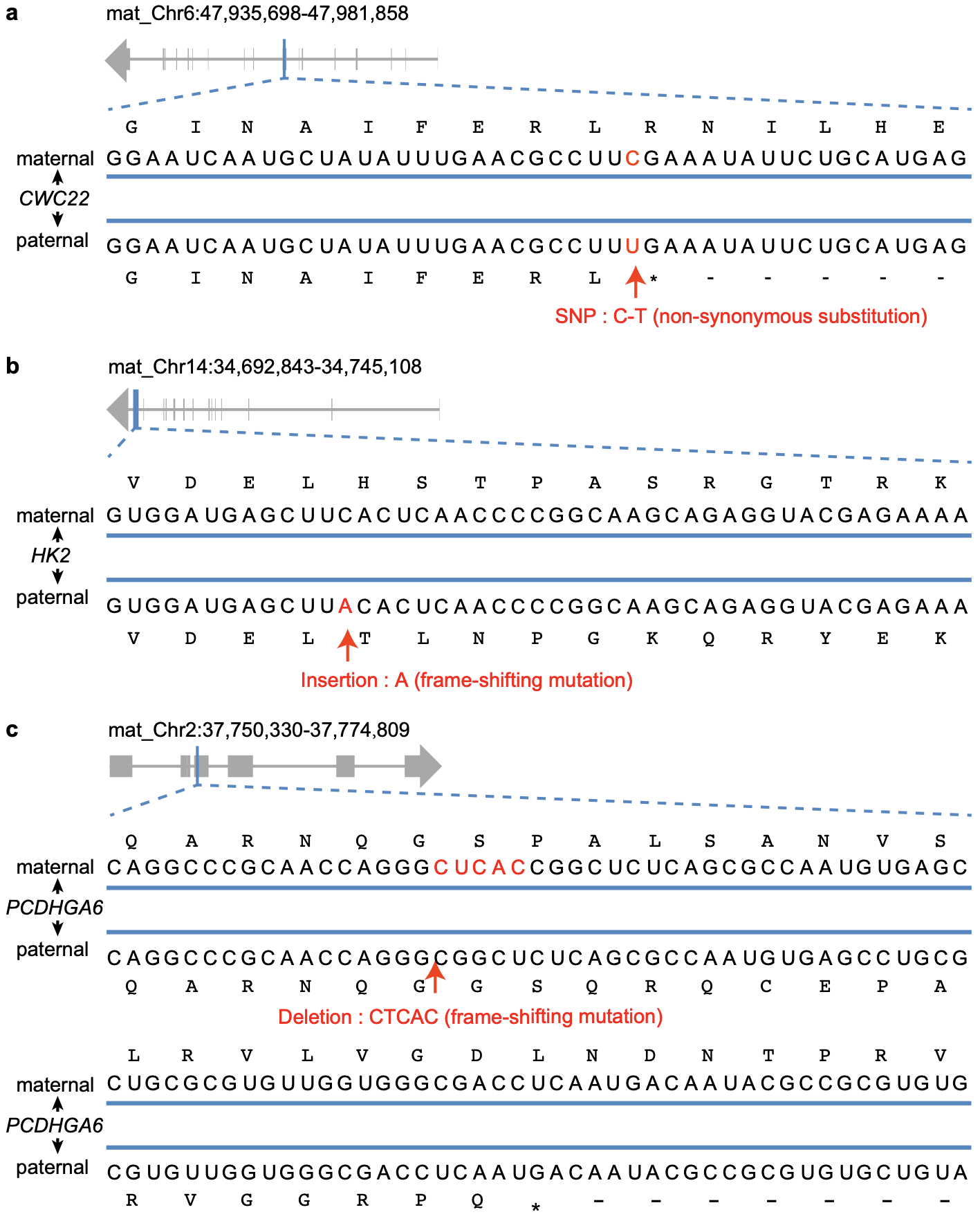


## Supplementary Figure 14. Different open-reading frames caused by non-synonymous substitution or frame-shifting mutations. a, A substitution (C<->T) occurred in the coding region of the *CWC22* gene between maternal and paternal marmoset genomes, resulting in a premature stop codon in the paternal transcribed mRNA. b, Frame-shifting mutations caused by an insertion in the paternal *HK2* and c, a deletion in the paternal *PCDHGA6* leads to a premature stop codon.


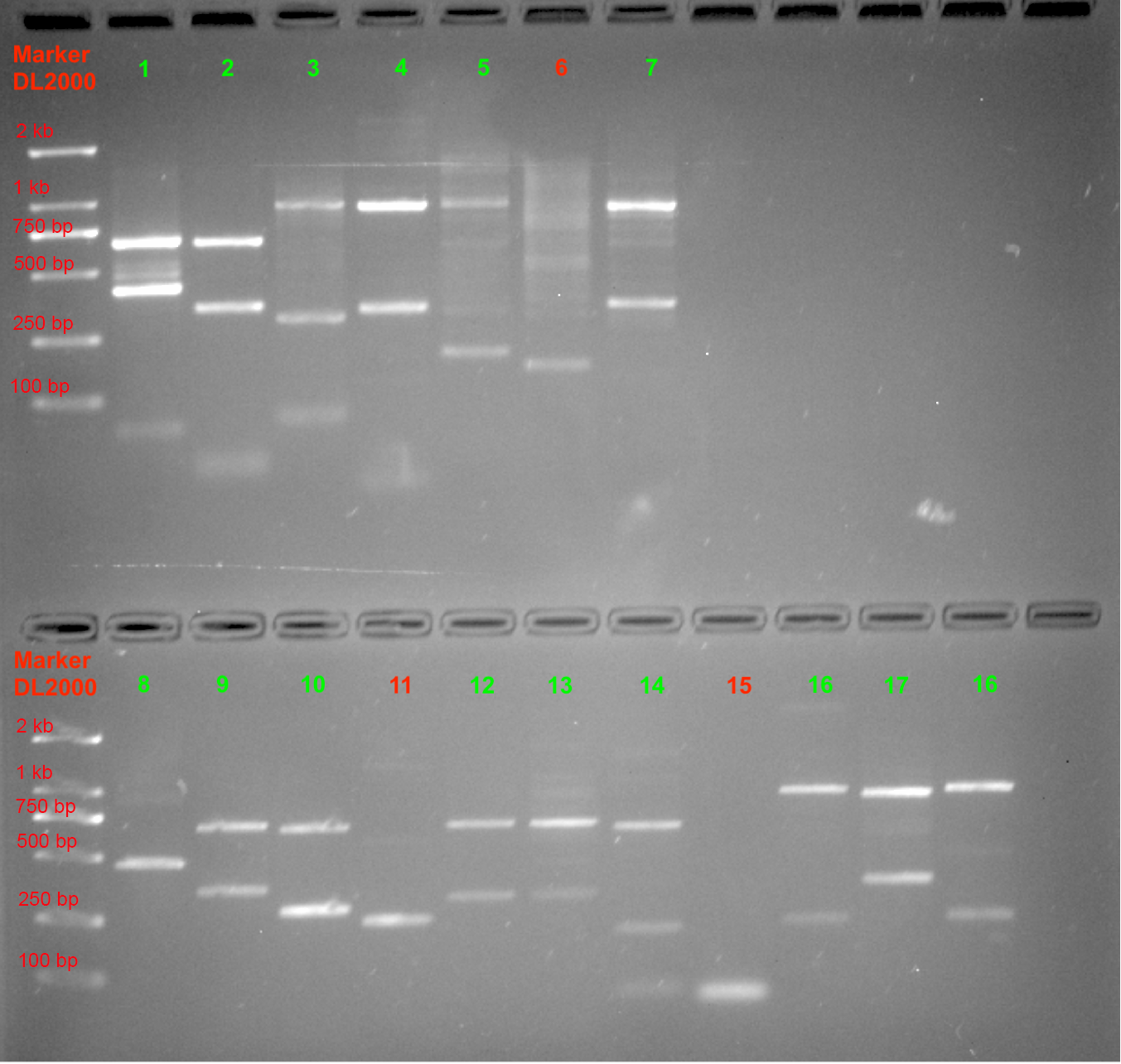


## Supplementary Figure 15. PCR validation of 17 randomly selected large indels in chromosome 1. The six gel bands on the left are length of 2 kb, 1 kb, 750 bp, 500 bp, 250 bp, and 100 bp, respectively. The green color represents the validation success whereas red represents failure. The numbers labeled are associated with indels in Supplementary Table 20. Of note, the last one (No.16) was ruled out because it was a repetition. PCR validation experiment aim to validate the variation between the two alleles of the F1 male, thus the experiments were performed based on the offspring DNA sample and replication is not applied in this study.

##
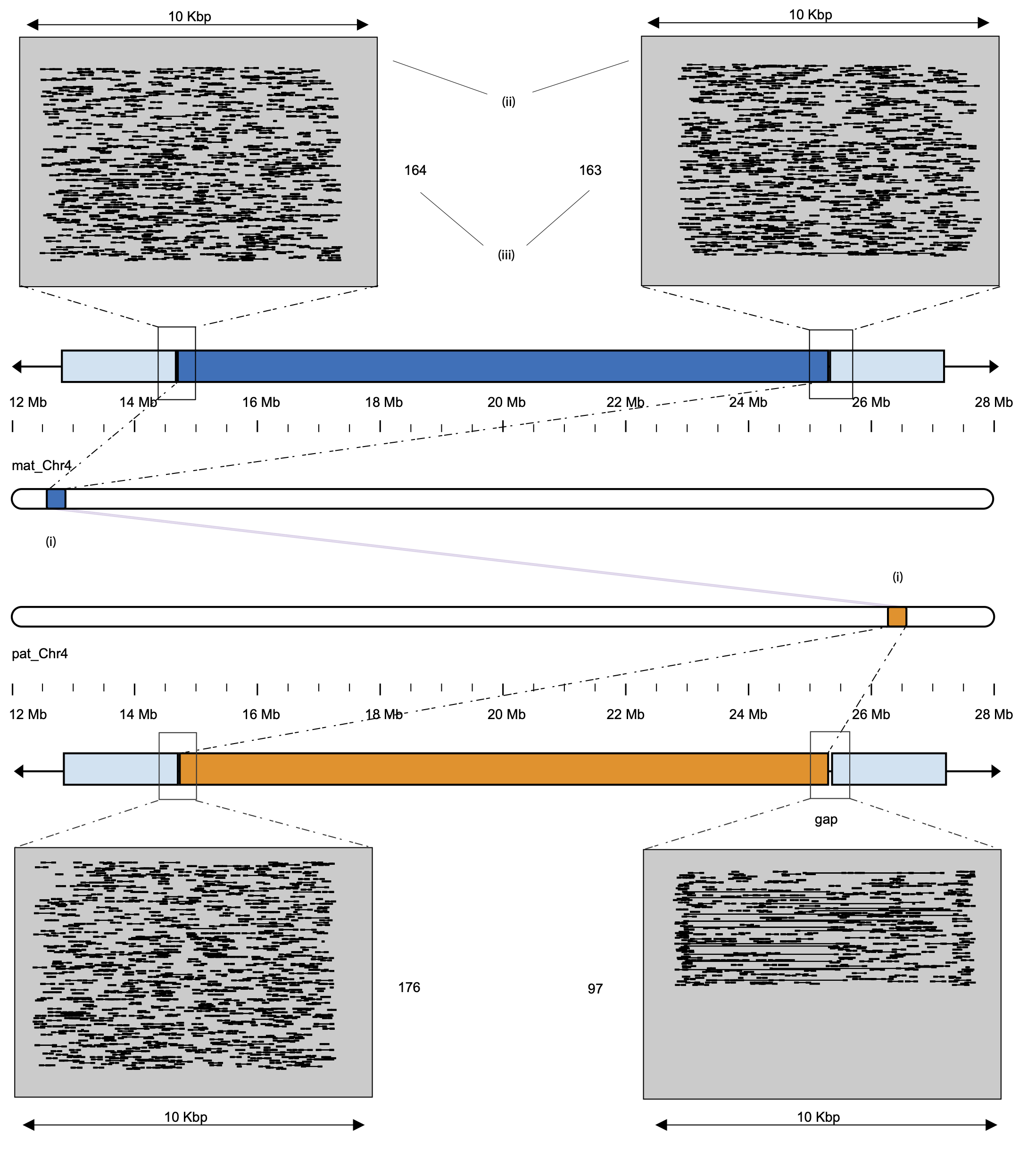
Supplementary Figure 16. Visualization of the longest SV between maternal and paternal assemblies. (i) A 304 kb length translocation on chromosome 4. (ii), The 10X linked read mapping to the flanking 5 kb region of each breakpoint. (iii) The 10X linked read number to support each breakpoint. Of note, there is a 10 bp gap beside the right breakpoint of paternal.

##
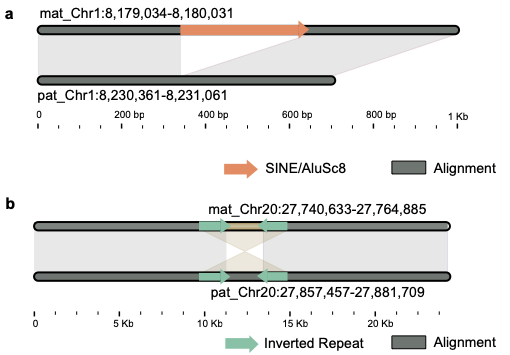


## Supplementary Figure 17. Visualization of indel and inversion mediated by repeat elements. a, An example of indels mediated by Alu sequence (red arrow). b, An example of inversion mediated by the inverted repeat elements (green arrows).
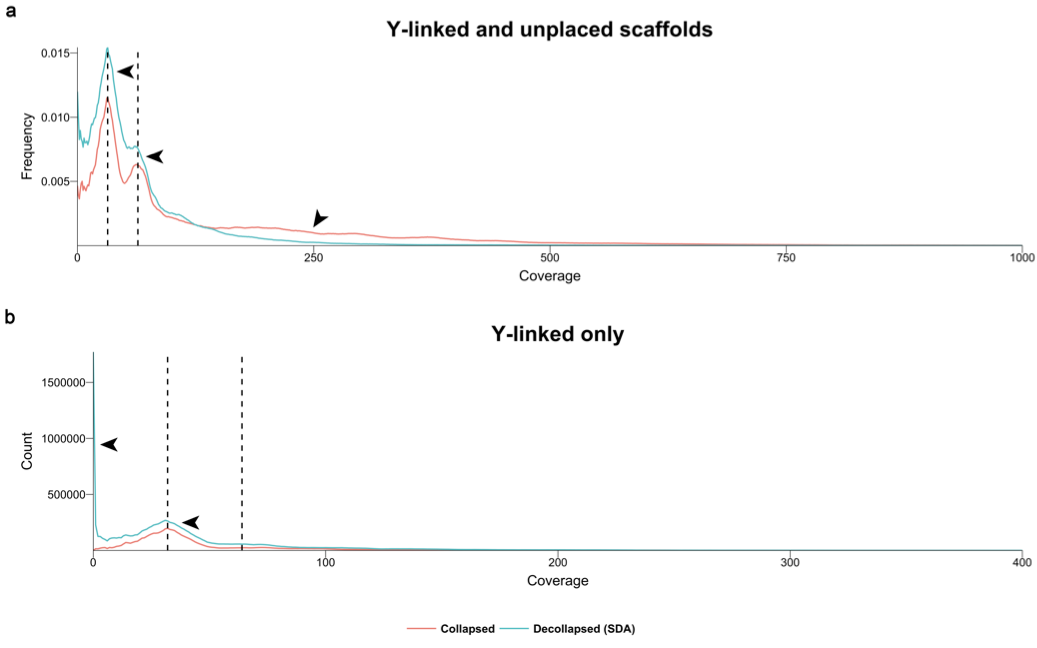


## Supplementary Figure 18. Decollapse of segmental duplications with SDA. a, SDA applied to all Y-linked and unplaced scaffolds. The frequency of bases at normal coverage is increased (first and second arrow), while the frequency of bases at high coverage is decreased (third arrow). b, The coverage considering only Y-linked scaffolds. The total number of bases at haploid coverage is increased by approximately 36% (first arrow). It should be noted that bases at 0 coverage have also markedly increased (second arrow), consistent with the difficulty of uniquely mapping reads belonging to decollapsed segmental duplications. Dashed lines represent the expected haploid and diploid peaks.

##

##

##


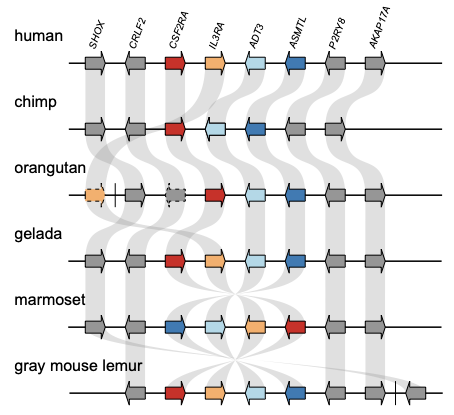


## Supplementary Figure 19. Synteny of *CSF2RA*, *IL3RA*, *ADT3*, *ASMTL,* and their flanking genes among primates confirms the specific inversion in marmoset. Regions are not drawn to scale. Fragmented genes are shown in shade. Vertical lines separate genes on different scaffolds.


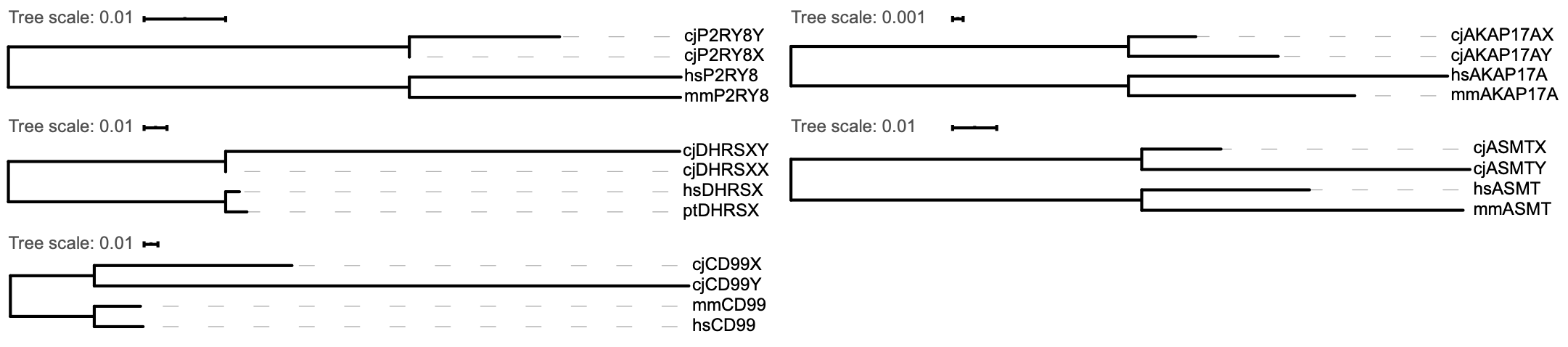


## Supplementary Figure 20. Phylogenetic tree of MSSDR genes. In all cases, the Y-copies of the MSSDR genes show a longer branch length than their X-copies, suggesting that Y-copy has accumulated more mutations than X-copy since their divergence. cj: marmoset, hs: human, mm: macaca, pt: chimp.

##
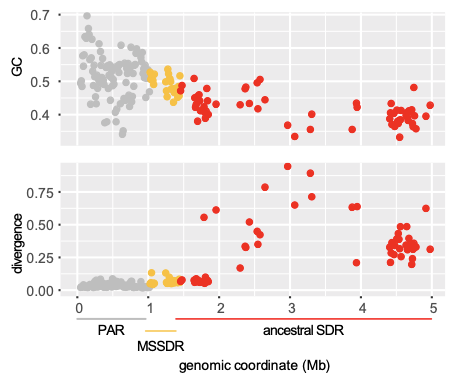


## Supplementary Figure 21. GC content and X-Y sequence divergence (estimated using the HKY85 model, as implemented in baseml of PAML) across the first 5 Mb of the marmoset sex chromosomes. Each point is an estimate for a 10 kb region and the position on the x-axis corresponds to marmoset X coordinates. Only regions with at least 1,000 alignable base pairs are shown. N = 180, 48, and 124 windows in PAR, MSSDR, and ancestral SDR, respectively.

##

##
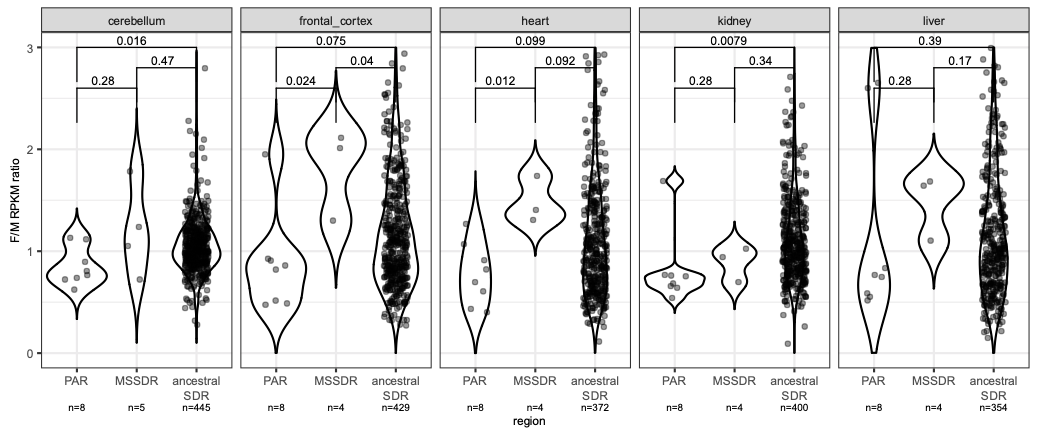


**Supplementary Figure 22. X-linked MSSDR genes show subtle female biased expression.** Boxplots show median, quantiles (boxes), and range (whiskers). Two-sided Wilcoxon rank-sum tests are performed.

##


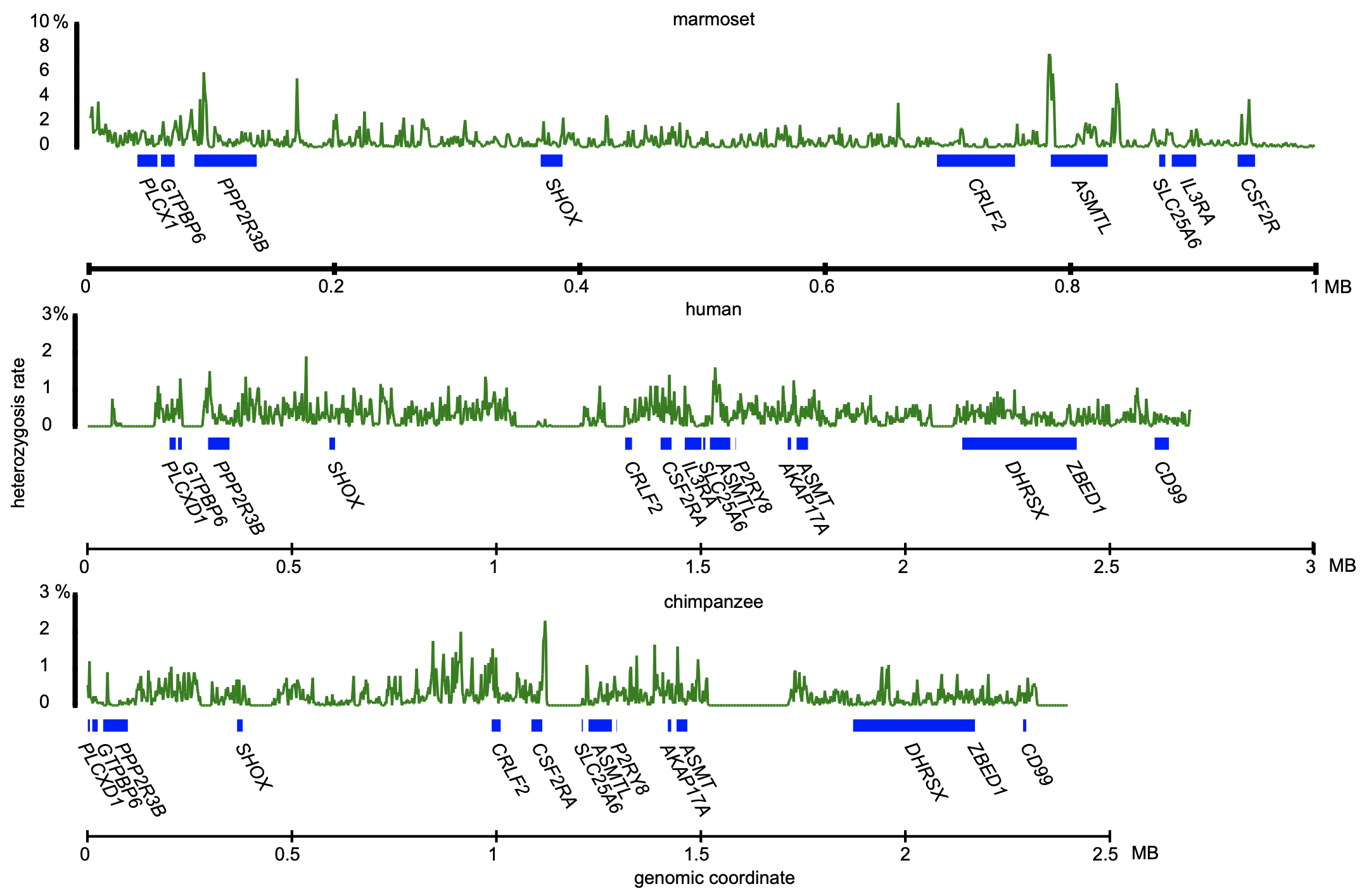


## Supplementary Figure 23. High heterozygosity rate in PARs of marmoset, human, and chimpanzee. Heterozygosity rate is 0.52%, 0.27%, and 0.24% in PARs of marmoset, human, and chimpanzee, all higher than that of the autosomes (marmoset 0.15%, human 0.14%, and chimp 0.12%).


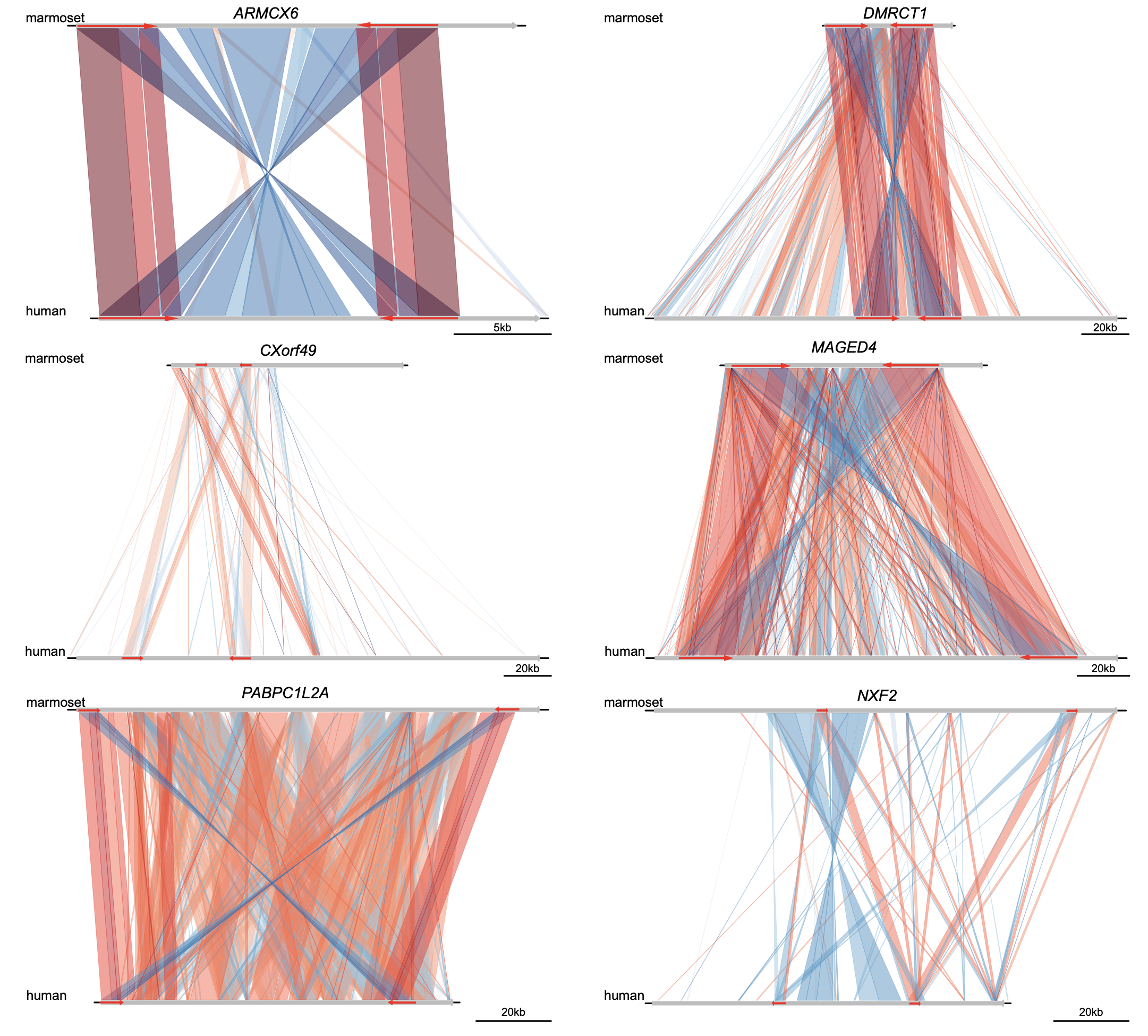


## Supplementary Figure 24. Pattern of duplication of the six X-linked genes that are ampliconic in both human and marmoset. Links between marmoset and human regions represent BLAST hits. They are colored in red (same sense) or in blue (anti-sense), the intensity of the color depending on the percentage of identity of the hit.

##

##
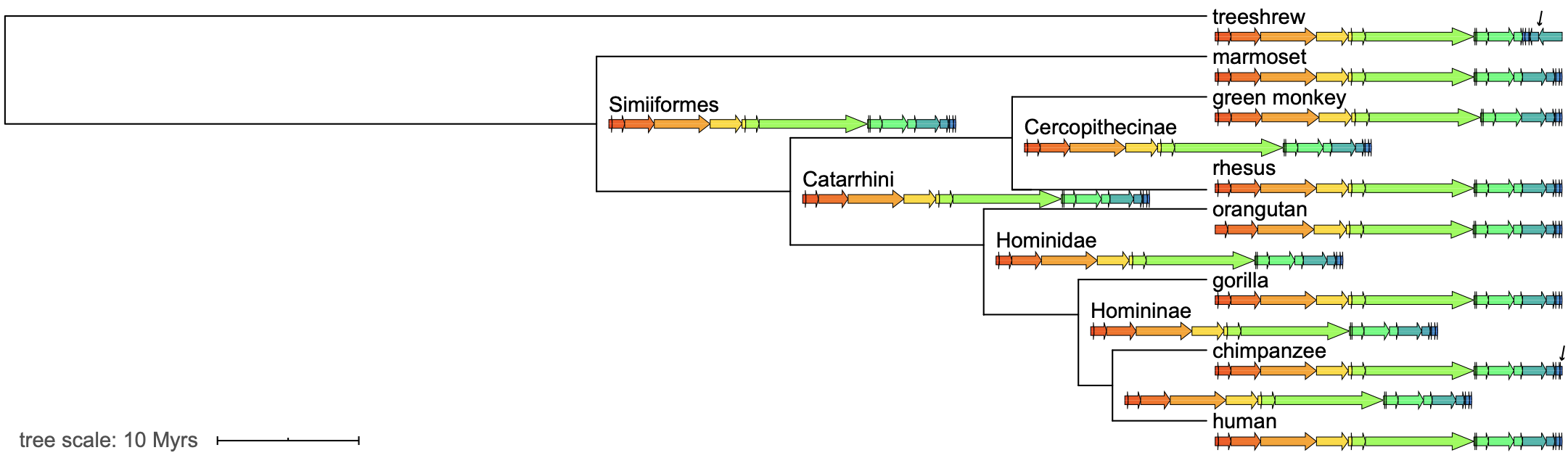


## Supplementary Figure 25. X chromosome structure evolution in primates. Each block represents a conserved block among the species during evolution. Inversions found during evolution are marked by arrows.


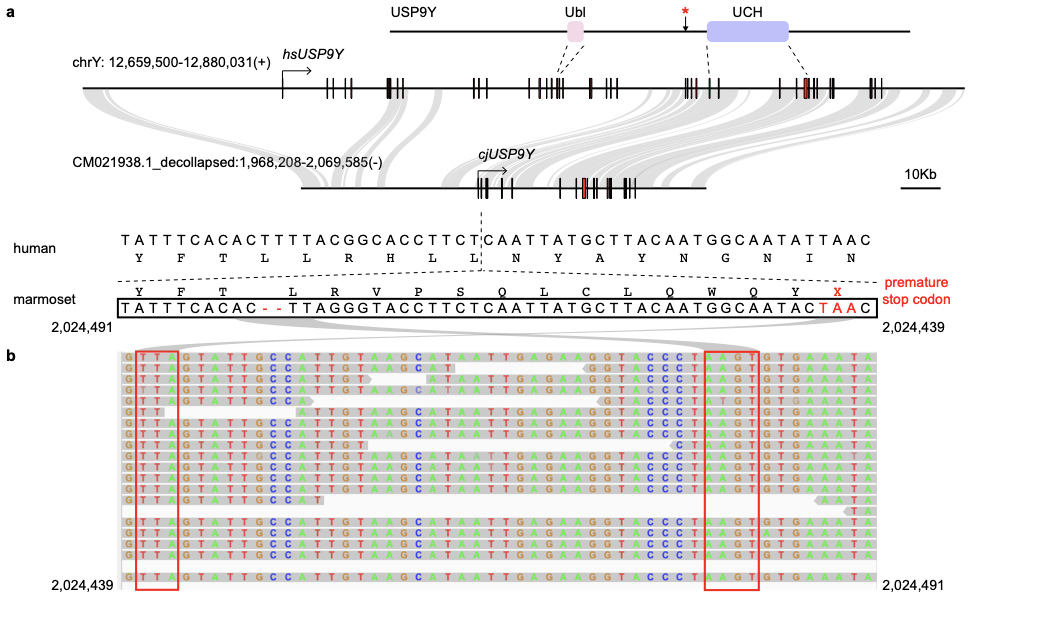


## Supplementary Figure 26. Examination of *USP9Y* in marmoset. a, Alignment of the *USP9Y* genomic region between human and marmoset. Asterisk is the location of the premature stop codon. The first 25 exons are deleted in marmoset, thus it is not able to translate the ubiquitin-like (Ubl) domain as in human. b, Alignment of 19 illumina short reads, verifying the 2bp deletion and the premature stop codon (red boxes) in the marmoset *USP9Y* gene.

##
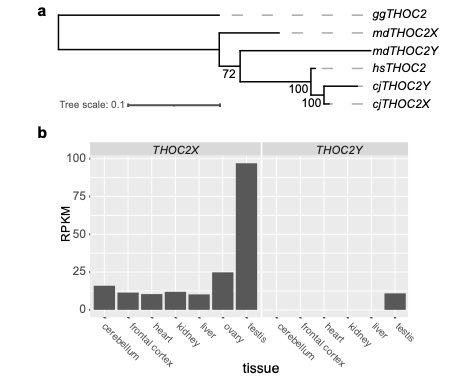


## Supplementary Figure 27. *THOC2X/Y* phylogeny and expression. a, Phylogenetic analysis of *THOC2X-Y*. The clustering of marmoset X-Y genes suggests that the gametologues were evolved after the split with human, but unlikely to be derived from the ancestral proto-Y as in opossum. gg: chicken, md: opossum, cj: marmoset, hs: human. b, Expression of *THOC2X-Y* in marmoset shows that both genes are overexpressed in testis.

##
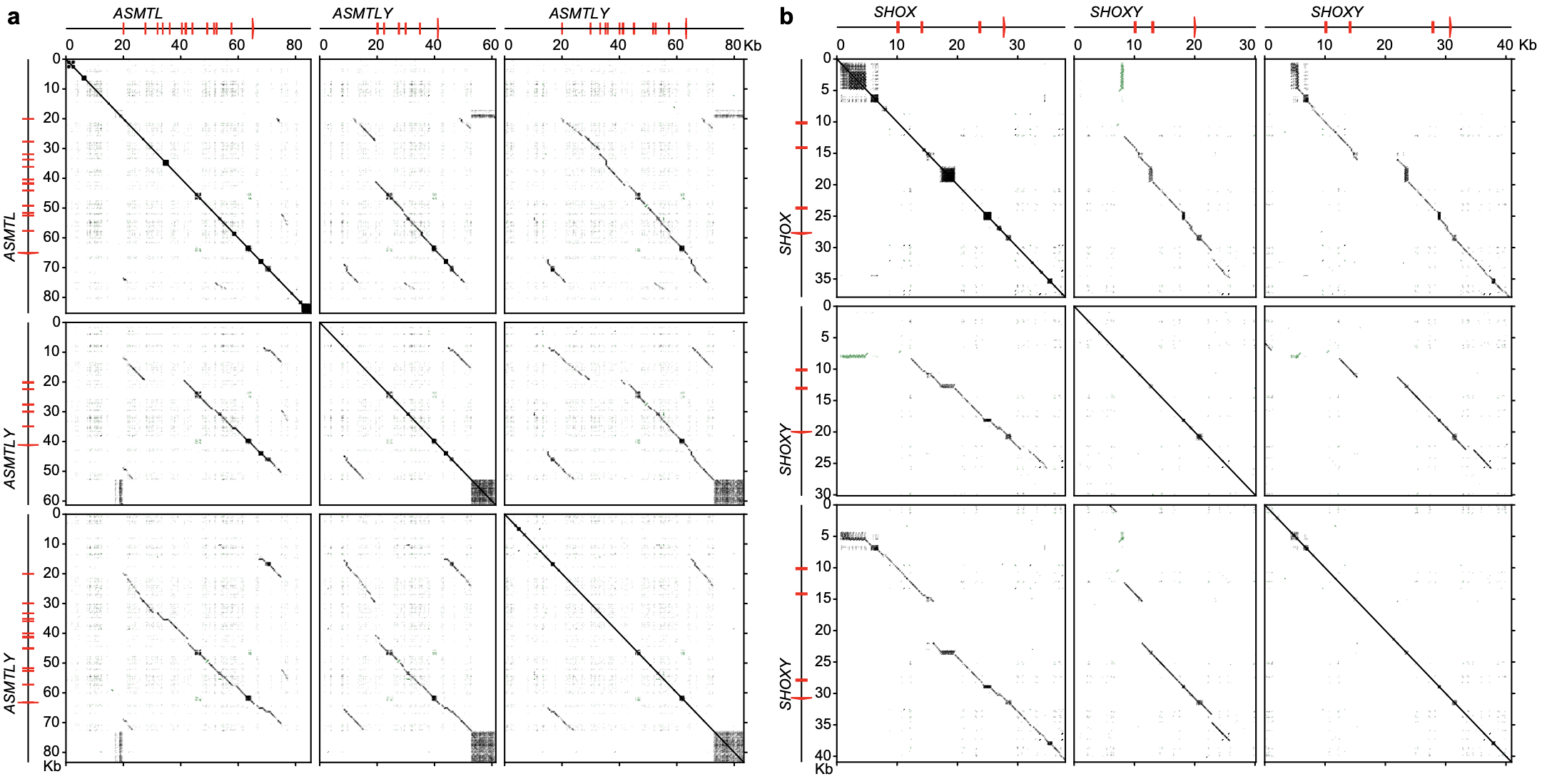


## Supplementary Figure 28. Dotplot of *ASMTL*-*ASTMLY*s (a) and *SHOX*-*SHOXY*s (b). The alignments between *ASMTL* and *ASMTLY*s, *SHOX* and *SHOXY*s, and the gene structures suggest that *ASMTLY*s and *SHOXY*s are derived from gene duplication events instead of retrotransposition.


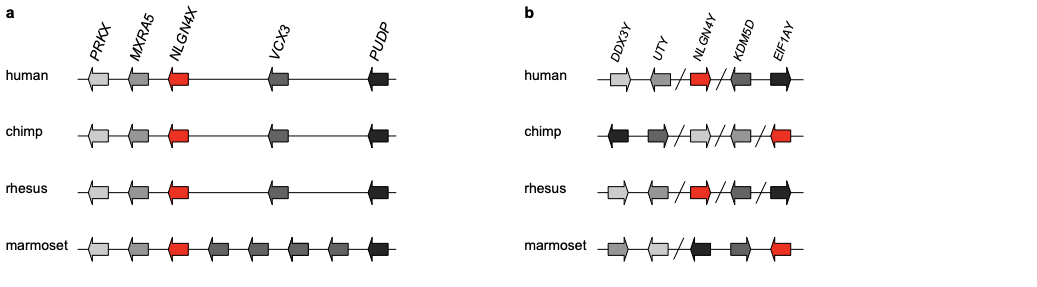


## Supplementary Figure 29. Synteny of *NLGN4X/Y* and their flanking genes. *NLGN4X* and its flanking genes show a conserved synteny among human, chimp, rhesus macaque, and marmoset, while *NLGN4Y* and its flanking genes remain partly conserved synteny among species, due to lineage specific rearrangements. Genes are color coded according to their orthologous relationship. Four *VCX3*s are found in the orthologous region in marmoset while there is only one in human, chimp, and rhesus macaque. Regions are not drawn to scale. Slashes separate genes that are not neighbouring each other.


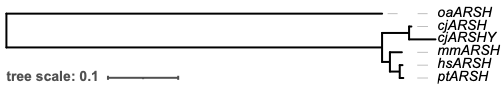


## Supplementary Figure 30. Phylogenetic analysis of gametologues *ARSHX-Y*. The clustering of Old World Monkey *ARSH*s and marmoset X-Y genes suggests that the gametologues evolved independently in these two lineages. oa: platypus, cj: marmoset, mm: rhesus, pt: chimp, hs: human.

**
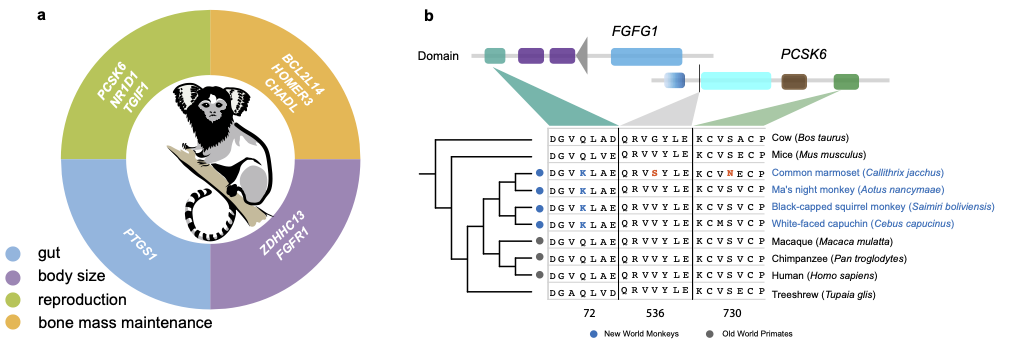
Supplementary Figure 31. Positively selected sites in marmoset related to biological aspects.** **a**, Nine positively selected genes are related to marmoset special biological traits, such as small body size, high fecundity of twinning, exudate-feeding, and maintaining bone density during aging without gonadal estrogen. **b**, *FGFR1* is under positive selection in the common ancestor of New World Monkeys (Q72K). *PCSK6* has two marmoset-specific PSG sites.


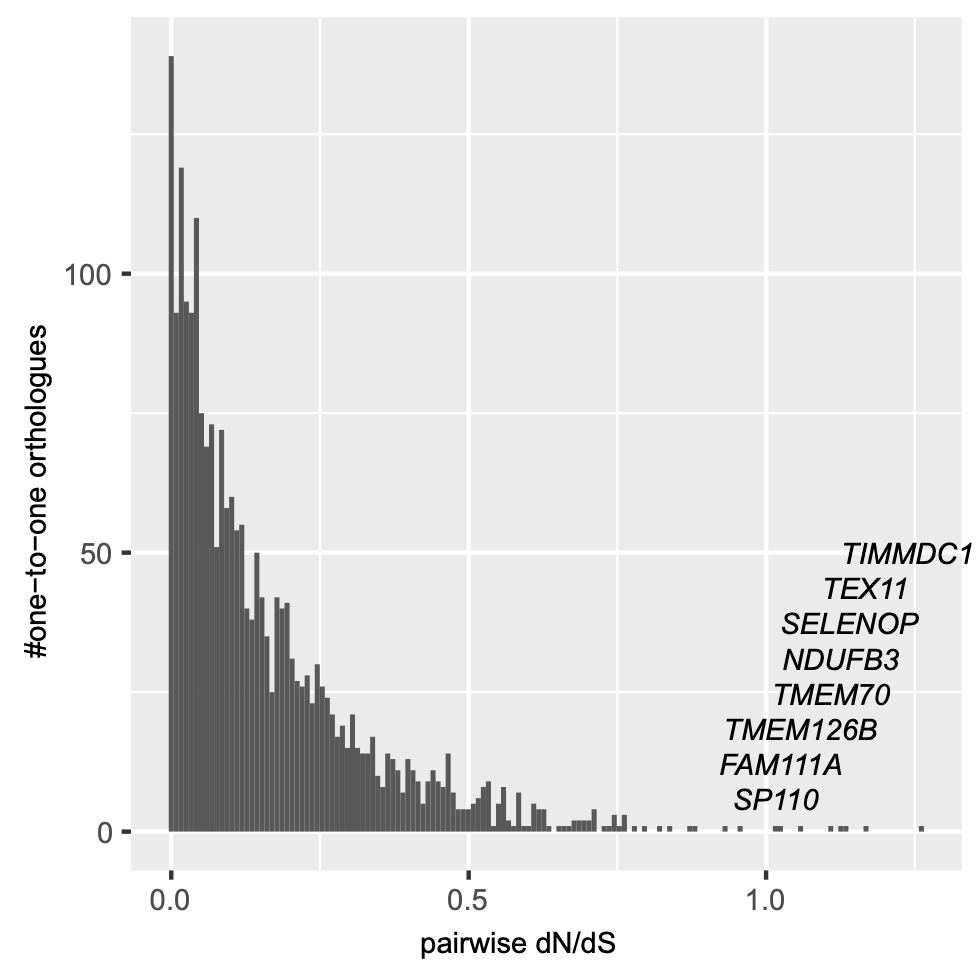


## Supplementary Figure 32. Pairwise dN/dS distribution of one-to-one orthologues related to brain development and neurodegenerative disease between human and marmoset. Names of the eight orthologues with pairwise dN/dS > 1 are also shown.

#
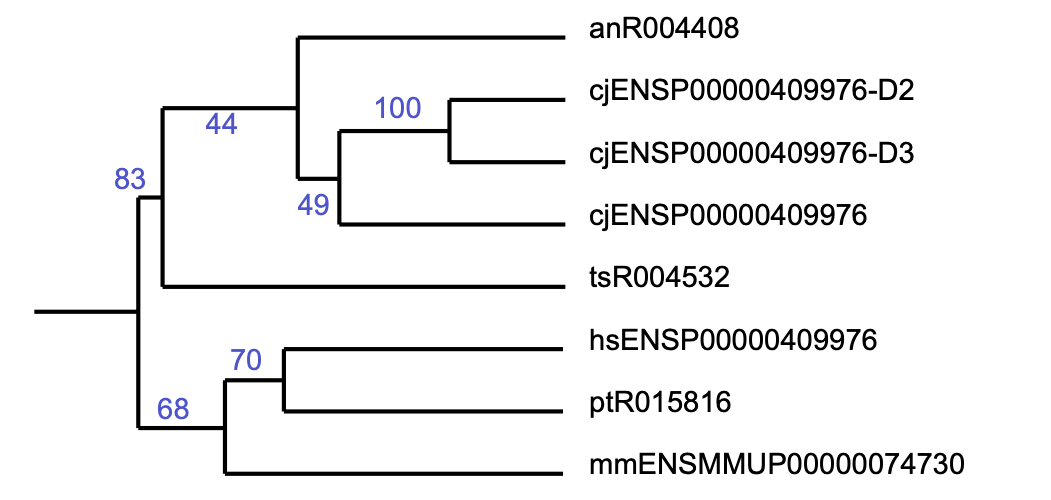


## Supplementary Figure 33. Phylogenetic tree of *APSA*. Gene tree shows lineage-specific duplications in marmoset.

##


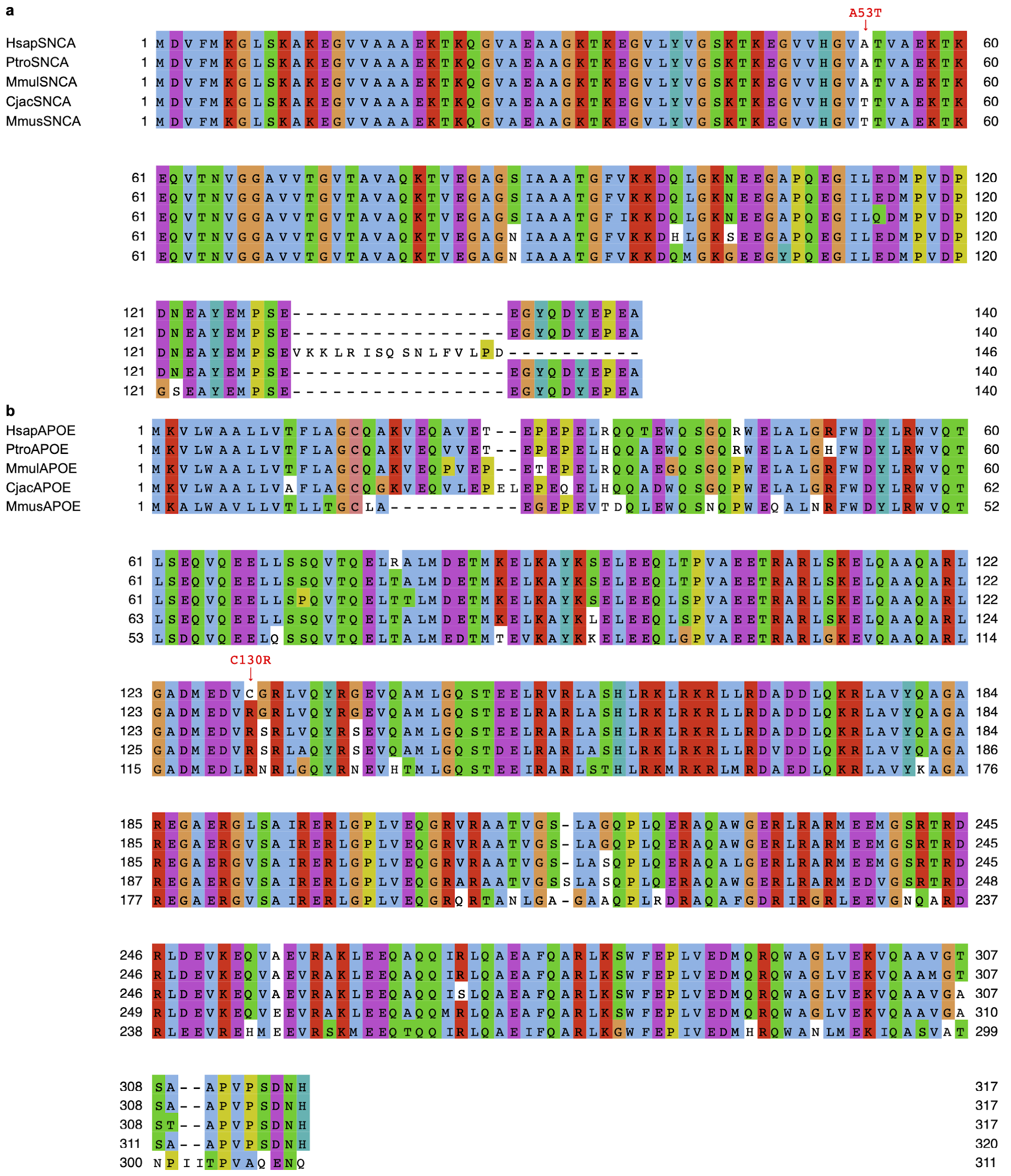


## Supplementary Figure 34. Protein alignments of SNCA (a) and APOE (b). Human pathogenic or risky mutation sites are highlighted with red arrows. Hsap: human, Ptro: chimp, Mmul: macaque, Cjac: marmoset, Mmus: mouse.

##
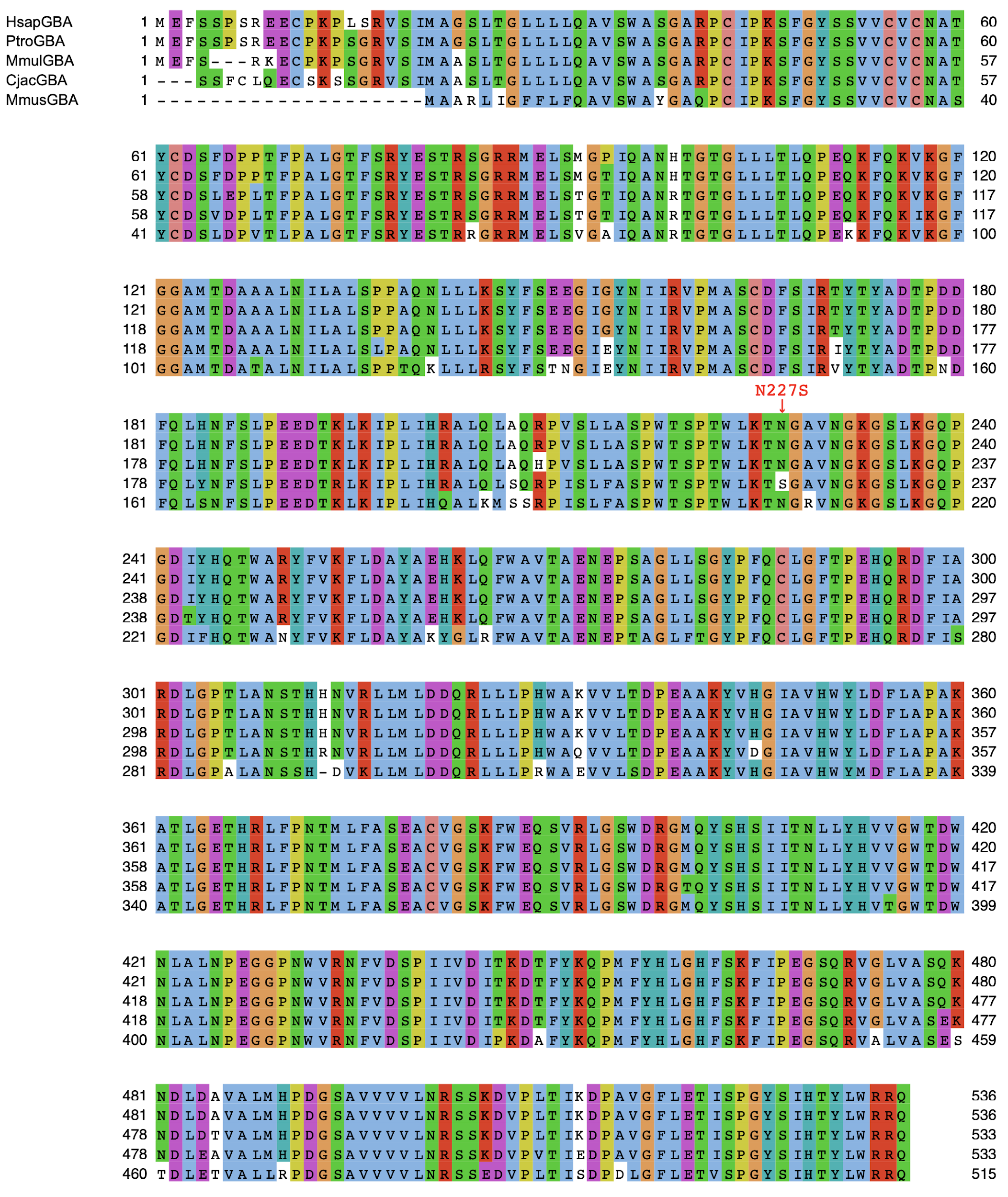
Supplementary Figure 35. Protein alignments of GBA. The human pathogenic or risky mutation site is highlighted with a red arrow. Hsap: human, Ptro: chimp, Mmul: macaque, Cjac: marmoset, Mmus: mouse.


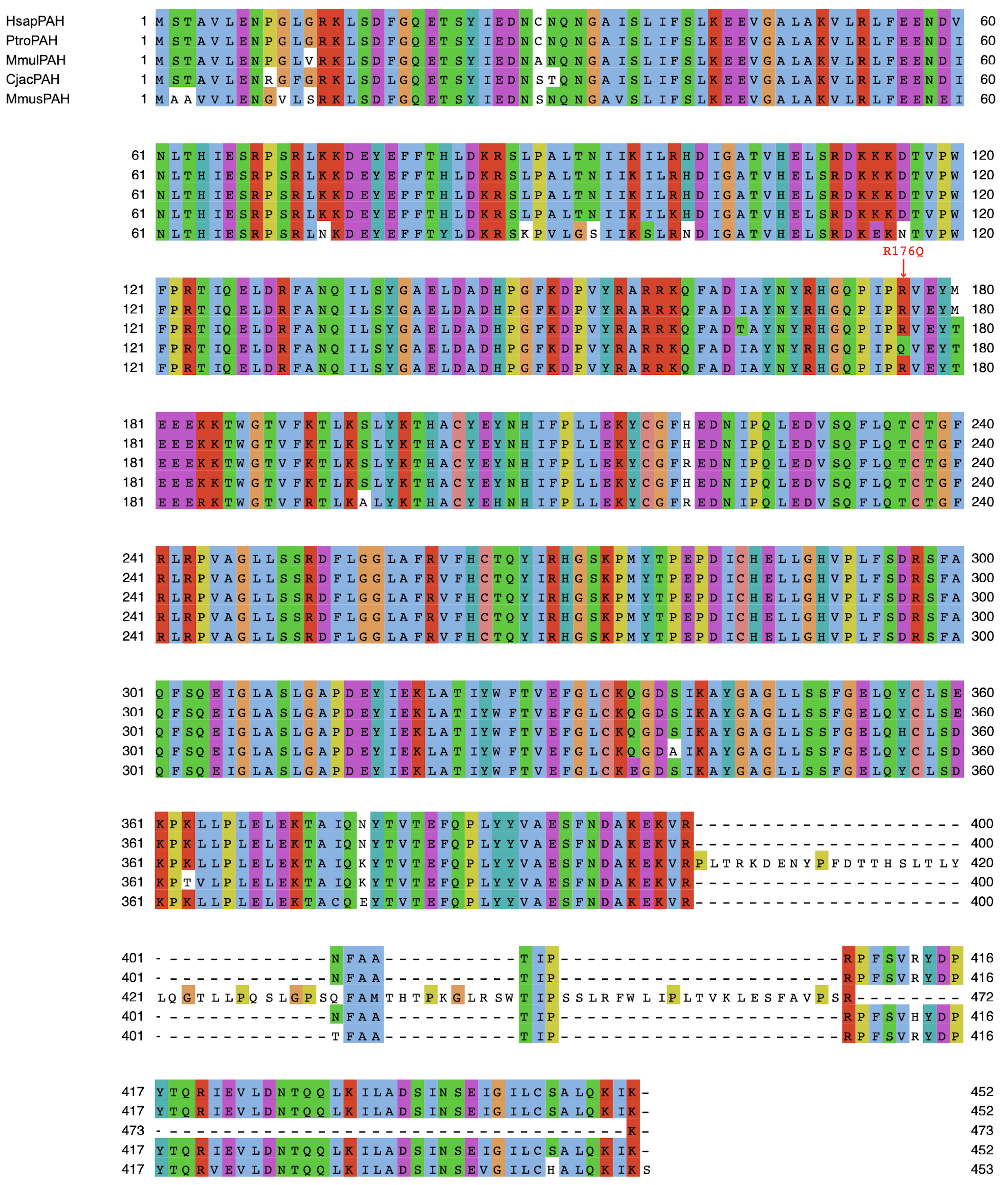


## Supplementary Figure 36. Protein alignments of PAH. The human pathogenic or risky mutation site is highlighted with a red arrow. Hsap: human, Ptro: chimp, Mmul: macaque, Cjac: marmoset, Mmus: mouse.

# **Supplementary Tables**

**Supplementary Table 1.** Sample metadata.

**Supplementary Table 2.** List of tools used in the trio-phased assembly process, actual versions used, and availability.

**Supplementary Table 3.** Additional functional and analytic tools used during assembly.

**Supplementary Table 4.** Completeness of marmoset assembly analyzed by correlation of assembled maternal and paternal chromosome lengths to size estimations of five published marmoset karyotypes.

**Supplementary Table 5.** Correlation of maternal autosomes, X chromosome, + paternal Y chromosome lengths to chromosome lengths estimated from five different published karyotypes.

**Supplementary Table 6.** Length statistics of total phased region, chimeric regions and non-chimeric region.

**Supplementary Table 7.** Third/fourth allele region ratio in mCalJac1.

**Supplementary Table 8.** Assembly statistics through each stage of the pipeline.

**Supplementary Table 9.** BUSCO analysis across marmoset assemblies.

**Supplementary Table 10.** Comparison of Iso-Seq mapped reads in different marmoset assemblies.

**Supplementary Table 11.** Using Iso-Seq data to evaluate the integrity of different marmoset assembly.

**Supplementary Table 12.** Intrachromosomal structural variations identified from the comparison of different marmoset assemblies

**Supplementary Table 13.** Conflicting chromosome anchor between different marmoset assemblies.

**Supplementary Table 14.** Supports of 10X linked reads at gaps within the breakage regions.

**Supplementary Table 15.** SNV detection and polishing error, sequencing error estimation

**Supplementary Table 16.** Experimental validation of SNVs.

**Supplementary Table 17.** Experimental validation of 285 small INDELs.

**Supplementary Table 18.** Run of homozygosity (ROH) analysis of 9 published samples and our trio samples.

**Supplementary Table 19.** Statistics of structural variations by chromosomes.

**Supplementary Table 20.** Experimental validation of 17 large indels.

**Supplementary Table 21.** 50 genes affected by translocations.

**Supplementary Table 22.** Experimental validation of 9 de novo mutations.

**Supplementary Table 23.** Identified X-linked in mCalJac1.

**Supplementary Table 24**. Identified Y-linked sequences in decollapsed mCalJac1 combining depth method and Hi-C method.

**Supplementary Table 25**. Statistics of X-linked and Y-linked sequences in available marmoset assembly. Fragmented genes and missing genes are evaluated using Iso Seq high quality transcripts produced in this study.

**Supplementary Table 26**. Y BAC alignment in mCalJac1 paternal assembly.

**Supplementary Table 27**. Genomic coordinates and pairwise values of marmoset gametologues.

**Supplementary Table 28**. Divergence estimates and numbers of aligned sites of the PAR and the genes in the MSSDR based on pairwise whole gene Cactus alignments of marmoset sex chromosomes (X and Y) and the human X chromosome (X_H).

**Supplementary Table 29**. Dates of birth for the parents-offspring trio.

**Supplementary Table 30**. Per position per year (PPPY) estimates of the autosomal mutation rate μ_A for different primates.

**Supplementary Table 31**. Estimates of the time of MSSDR formation t using eqs. (1) and (2) and a range of values for μ_A and α.

**Supplementary Table 32**. Expression profile of marmoset Y-linked genes.

**Supplementary Table 33.** Positively selected genes in common marmoset.

**Supplementary Table 34.** Positively selected sites in common marmoset related to biological aspects.

**Supplementary Table 35.** Positively selected genes in NWM.

**Supplementary Table 36**. Genes related to brain development and disease-causing showing multi-copy in marmoset.

**Supplementary Table 37**. Genes related to brain development showing pairwise dN/dS > 1 between human and marmoset.

**Supplementary Table 38.** Confirmation of marmoset codons which encode amino acids that are different from human.

**Supplementary Table 39.** Identified Y-linked sequences in mCalJac1 (GCA_011100555.1) before SDA, combining depth method and Hi-C method.

# **Supplementary References**

1 Jain, C., Koren, S., Dilthey, A., Phillippy, A. M. & Aluru, S. A fast adaptive algorithm for computing whole-genome homology maps. *Bioinformatics* **34**, i748-i756, doi:10.1093/bioinformatics/bty597 (2018).

2 Vurture, G. W. *et al.* GenomeScope: fast reference-free genome profiling from short reads. *Bioinformatics* **33**, 2202-2204, doi:10.1093/bioinformatics/btx153 (2017).

3 Koren, S. *et al.* De novo assembly of haplotype-resolved genomes with trio binning. *Nat Biotechnol* (2018).

4 Rhie, A. *et al.* Towards complete and error-free genome assemblies of all vertebrate species. *bioRxiv*, 2020.2005.2022.110833 (2020).

5 Formenti, G. *et al.* Complete vertebrate mitogenomes reveal widespread gene duplications and repeats. *bioRxiv*, 2020.2006.2030.177956 (2020).

6 Guan, D. *et al.* Identifying and removing haplotypic duplication in primary genome assemblies. *Bioinformatics* **36**, 2896-2898 (2020).

7 Ghurye, J. *et al.* Integrating Hi-C links with assembly graphs for chromosome-scale assembly. *PLoS Comput Biol* **15**, e1007273 (2019).

8 Chow, W. *et al.* gEVAL - a web-based browser for evaluating genome assemblies. *Bioinformatics* **32**, 2508-2510 (2016).

9 Benirschke, K., Anderson, J. M. & Brownhill, L. E. Marrow chimerism in marmosets. *Science* **138**, 513-515 (1962).

10 Ardito, G., Lamberti, L., Bigatti, P., Stanyon, R. & Govone, D. NOR distribution and satellite associations in Callithrix jacchus. *Caryologia* **40**, 185-194 (1987).

11 Sherlock, J. K., Griffin, D. K., Delhanty, J. D. A. & Parrington, J. M. Homologies between human and marmoset (*Callithrix jacchus*) chromosomes revealed by comparative chromosome painting. *Genomics* **33**, 214-219 (1996).

12 Cellamare, A. *et al.* New insights into centromere organization and evolution from the white-cheeked gibbon and marmoset. *Molecular biology and evolution* **26**, 1889-1900 (2009).

13 Porubsky, D. *et al.* Fully phased human genome assembly without parental data using single-cell strand sequencing and long reads. *Nat Biotechnol*, 1-7 (2020).

14 Kurtz, S. *et al.* Versatile and open software for comparing large genomes. *Genome Biol* **5**, R12 (2004).

15 Li, H. Minimap2: pairwise alignment for nucleotide sequences. *Bioinformatics* **34**, 3094-3100 (2018).

16 Li, H. & Durbin, R. Fast and accurate short read alignment with Burrows-Wheeler transform. *Bioinformatics* **25**, 1754-1760 (2009).

17 Servant, N. *et al.* HiC-Pro: an optimized and flexible pipeline for Hi-C data processing. *Genome Biol* **16**, 259 (2015).

18 You, F. M. *et al.* BatchPrimer3: a high throughput web application for PCR and sequencing primer design. *BMC bioinformatics* **9**, 253 (2008).

19 Camacho, C. *et al.* BLAST+: architecture and applications. *BMC Bioinformatics* **10**, 421 (2009).

20 Fu, L., Niu, B., Zhu, Z., Wu, S. & Li, W. CD-HIT: accelerated for clustering the next-generation sequencing data. *Bioinformatics* **28**, 3150-3152 (2012).

21 Altschul, S. F., Gish, W., Miller, W., Myers, E. W. & Lipman, D. J. Basic local alignment search tool. *J Mol Biol* **215**, 403-410 (1990).

22 Birney, E., Clamp, M. & Durbin, R. GeneWise and Genomewise. *Genome Res* **14**, 988-995 (2004).

23 Slater, G. S. & Birney, E. Automated generation of heuristics for biological sequence comparison. *BMC Bioinformatics* **6**, 31 (2005).

24 McKenna, A. *et al.* The Genome Analysis Toolkit: a MapReduce framework for analyzing next-generation DNA sequencing data. *Genome Res* **20**, 1297-1303 (2010).

25 Li, H. *et al.* The sequence alignment/map format and SAMtools. *Bioinformatics* **25**, 2078-2079 (2009).

26 Sedlazeck, F. J. *et al.* Accurate detection of complex structural variations using single-molecule sequencing. *Nat Methods* **15**, 461-468 (2018).

27 Sato, K. *et al.* Resequencing of the common marmoset genome improves genome assemblies and gene-coding sequence analysis. *Sci Rep* **5**, 16894 (2015).

28 Kendig, K. I. *et al.* Sentieon DNASeq Variant Calling Workflow Demonstrates Strong Computational Performance and Accuracy. *Front Genet* **10**, 736 (2019).

29 Li, H. A statistical framework for SNP calling, mutation discovery, association mapping and population genetical parameter estimation from sequencing data. *Bioinformatics* **27**, 2987-2993 (2011).

30 Wickham, H. ggplot2: Elegant Graphics for Data Analysis. Ggplot2: Elegant Graphics for Data Analysis: 1-212. (doi 10.1007/978-0-387-98141-3, 2009).

31 Krzywinski, M. *et al.* Circos: an information aesthetic for comparative genomics. *Genome Res* **19**, 1639-1645 (2009).

32 Rhie, A., Walenz, B. P., Koren, S. & Phillippy, A. M. Merqury: reference-free quality, completeness, and phasing assessment for genome assemblies. *bioRxiv*, 2020.2003.2015.992941 (2020).

33 Goel, M., Sun, H., Jiao, W. B. & Schneeberger, K. SyRI: finding genomic rearrangements and local sequence differences from whole-genome assemblies. *Genome Biol* **20**, 277 (2019).

34 Paten, B. *et al.* Cactus: Algorithms for genome multiple sequence alignment. *Genome Res* **21**, 1512-1528, doi:10.1101/gr.123356.111 (2011).

35 Harris, R. S. Improved pairwise Alignmnet of genomic DNA. (2007).

36 Loytynoja, A. Phylogeny-aware alignment with PRANK. *Methods Mol Biol* **1079**, 155-170 (2014).

37 Talavera, G. & Castresana, J. Improvement of phylogenies after removing divergent and ambiguously aligned blocks from protein sequence alignments. *Syst Biol* **56**, 564-577 (2007).

38 Yang, Z. PAML 4: phylogenetic analysis by maximum likelihood. *Mol Biol Evol* **24**, 1586-1591 (2007).

39 Kim, D., Langmead, B. & Salzberg, S. L. HISAT: a fast spliced aligner with low memory requirements. *Nat Methods* **12**, 357-360 (2015).

40 Anders, S. & Huber, W. Differential expression analysis for sequence count data. *Genome Biol* **11**, R106 (2010).

41 Yanai, I. *et al.* Genome-wide midrange transcription profiles reveal expression level relationships in human tissue specification. *Bioinformatics* **21**, 650-659 (2005).

42 Stamatakis, A. RAxML version 8: a tool for phylogenetic analysis and post-analysis of large phylogenies. *Bioinformatics* **30**, 1312-1313 (2014).

43 Lucotte, E. A. *et al.* Dynamic copy number evolution of X- and Y-Linked ampliconic genes in human populations. *Genetics* **209**, 907-920 (2018).

44 Guy, L., Kultima, J. R. & Andersson, S. G. genoPlotR: comparative gene and genome visualization in R. *Bioinformatics* **26**, 2334-2335, doi:10.1093/bioinformatics/btq413 (2010).

45 Avdeyev, P., Jiang, S., Aganezov, S., Hu, F. & Alekseyev, M. A. Reconstruction of Ancestral Genomes in Presence of Gene Gain and Loss. *J Comput Biol* **23**, 150-164, doi:10.1089/cmb.2015.0160 (2016).

46 Li, L., Stoeckert, C. J., Jr. & Roos, D. S. OrthoMCL: identification of ortholog groups for eukaryotic genomes. *Genome Res* **13**, 2178-2189 (2003).

47 Ruan, J. *et al.* TreeFam: 2008 Update. *Nucleic Acids Res* **36**, D735-740 (2008).

48 Kaul, R., Gao, G. P., Balamurugan, K. & Matalon, R. Cloning of the human aspartoacylase cDNA and a common missense mutation in Canavan disease. *Nat Genet* **5**, 118-123 (1993).

49 Surendran, S., Campbell, G. A., Tyring, S. K. & Matalon, R. Aspartoacylase gene knockout results in severe vacuolation in the white matter and gray matter of the spinal cord in the mouse. *Neurobiol Dis* **18**, 385-389 (2005).

50 Ge, J., Wang, S., Wu, Y., Tang, H. & Yuepeng, E. Performance improvement for source mobility in named data networking based on global–local FIB updates. *Peer-to-Peer Networking and Applications* **9**, 670-680 (2016).

51 Collins, C. E., Leitch, D. B., Wong, P., Kaas, J. H. & Herculano-Houzel, S. Faster scaling of visual neurons in cortical areas relative to subcortical structures in non-human primate brains. *Brain Struct Funct* **218**, 805-816 (2013).

52 Guarani, V. *et al.* TIMMDC1/C3orf1 functions as a membrane-embedded mitochondrial complex I assembly factor through association with the MCIA complex. *Mol Cell Biol* **34**, 847-861 (2014).

53 Kremer, L. S. *et al.* Genetic diagnosis of Mendelian disorders via RNA sequencing. *Nat Commun* **8**, 15824 (2017).

54 Solovyev, N. Selenoprotein P and its potential role in Alzheimer's disease. *Hormones (Athens)* **19**, 73-79 (2020).
