# Supplementary material for: Evolutionary and biomedical insights from a marmoset diploid genome assembly
Source: Nature. 2021 Apr 28;594(7862):227–33. doi: 10.1038/s41586-021-03535-x (PMC8189906; doi:10.1038/s41586-021-03535-x)
Supplement: Supplementary file 2 — Reporting Summary [file 41586_2021_3535_MOESM2_ESM.pdf]

nature research

Corresponding author(s): Guojie Zhang

Last updated by author(s): Mar 31, 2021

Reporting Summary

Nature Research wishes to improve the reproducibility of the work that we publish. This form provides structure for consistency and transparency in reporting. For further information on Nature Research policies, see our Editorial Policies and the Editorial Policy Checklist.

Statistics

For all statistical analyses, confirm that the following items are present in the figure legend, table legend, main text, or Methods section.

n/a	Confirmed	
☐	☒	The exact sample size (*n*) for each experimental group/condition, given as a discrete number and unit of measurement
☐	☒	A statement on whether measurements were taken from distinct samples or whether the same sample was measured repeatedly
☐	☒	The statistical test(s) used AND whether they are one- or two-sided<br>*Only common tests should be described solely by name; describe more complex techniques in the Methods section.*
☒	☐	A description of all covariates tested
☐	☒	A description of any assumptions or corrections, such as tests of normality and adjustment for multiple comparisons
☐	☒	A full description of the statistical parameters including central tendency (e.g. means) or other basic estimates (e.g. regression coefficient) AND variation (e.g. standard deviation) or associated estimates of uncertainty (e.g. confidence intervals)
☐	☒	For null hypothesis testing, the test statistic (e.g. *F*, *t*, *r*) with confidence intervals, effect sizes, degrees of freedom and *P* value noted<br>*Give P values as exact values whenever suitable.*
☒	☐	For Bayesian analysis, information on the choice of priors and Markov chain Monte Carlo settings
☒	☐	For hierarchical and complex designs, identification of the appropriate level for tests and full reporting of outcomes
☐	☒	Estimates of effect sizes (e.g. Cohen's *d*, Pearson's *r*), indicating how they were calculated

Our web collection on statistics for biologists contains articles on many of the points above.

Software and code

Policy information about availability of computer code

Data collection	Data collection did not involve any software or code.
Data analysis	Common bioinformatic and statistical analysis software packages were used, including: TrioCanu (v1.8+287), smrtlink (v6.0.0.47841), purge_dups (v1.0.0), scaff10x (v4.1.0), Bionano Solve (v3.2.1_04122018), Salsa2 (v2.2), mitoVGP pipeline (v2.2), longranger (v2.2.2), freebayes (v1.3.1), gEVAL (https://vgp-geval.sanger.ac.uk), Mummer (v3.23), minimap2 (v2.13), bwa (v0.7.17-r1188), refaligner (7437.7523rel), hicpro (v2.10.0), svmu (v0.4-alpha), Assemblytics (v1.2), SyRi (v1.0), Integrative Genomics Viewer (v2.8.6), GATK (v4.1.4.1), samtools (v1.8 & v1.2), NGMLR (v0.2.7), BCFtools (v1.8, v1.9-102-g958180e), ggplot2 (v3.3.2), circos (v0.69-8), BatchPrimer3 (v1.0), BLAST+ (v2.9.0+), cdhit (v4.8.1), BLAST (v2.7.1, v2.2.26), GeneWise (v2.4.1), exonerate (v2.2.0), LASTZ (v1.04.00), PRANK (v150803, v170427), Gblocks (v0.91b), PAML (v4.8 & v4.9i),HISTA2 (v2.0.5), DESeq (v1.9.12), RaxML (v8.2.9), orthoMCL (v1.4), TreeBest (v1.9.2), Jalview (v2.11.1.0), Mashmap (v2.0), proc10xG (v0.0.2), meryl (v1.0), Merqury (v1.0), genoPlotR (v0.8.9), MGRA2 (v2.2), SDA (git commit 4ca0c07), guidance (v2.02)<br>Custom scripts are open source and available on GitHub at https://github.com/comery/marmoset and https://github.com/gf777/misc/tree/master/marmoset%20Y.

For manuscripts utilizing custom algorithms or software that are central to the research but not yet described in published literature, software must be made available to editors and reviewers. We strongly encourage code deposition in a community repository (e.g. GitHub). See the Nature Research guidelines for submitting code & software for further information.

Data

Policy information about availability of data

All manuscripts must include a data availability statement. This statement should provide the following information, where applicable:

- Accession codes, unique identifiers, or web links for publicly available datasets
- A list of figures that have associated raw data
- A description of any restrictions on data availability

Raw sequencing data for the marmoset trio is available under the GenomeArk github (https://vgp.github.io/genomeark/Callithrix_jacchus/). Curatorial information and data mappings to maternal and paternal assemblies are available on the genome evaluation browser, gEVAL (https://vgp-geval.sanger.ac.uk/all_genomes.html). The maternal, paternal, and combined (paternal autosomes and Y chromosome + maternal X chromosome + mitochondrial) assemblies, as well as PacBio Iso-Seq data for annotation, are available under the NCBI BioProject PRJNA560230 (http://www.ncbi.nlm.nih.gov/bioproject/PRJNA560230). The genome assemblies have also been deposited at CNSA of CNGBdb with accession CNP0001310 and CNP0001311. Chimpanzee NGS reads are obtained from ERP002376. The human SNV data of HG00096 was obtained from https://www.internationalgenome.org/. Published marmoset genomes are obtained with accession code GCA_000004665.1, GCA_001269965.1, GCA_002754865.1, GCA_009663435.1, GCA_009811775.1. Genomes used in brain related genes study include: human (hg38), marmoset (mCalJac1), chimpanzee (Clint_PTRv2), rhesus macaque (rheMacS), Ma's night monkey (Anan_2.0), and Chinese tree shrew (TS_2.0). Genomes used in positive selection section include: cow, human, chimpanzee, mouse from Ensembl 98 and Chinese tree shrew (TS_2.0), Cebus capucinus (GCF_001604975.1), Saimiri boliviensis (GCF_000235385.1), Aotus nancymaae (GCF_000952055.2) from NCBI.

Field-specific reporting

Please select the one below that is the best fit for your research. If you are not sure, read the appropriate sections before making your selection.

☒ Life sciences ☐ Behavioural & social sciences ☐ Ecological, evolutionary & environmental sciences

For a reference copy of the document with all sections, see nature.com/documents/nr-reporting-summary-flat.pdf

Life sciences study design

All studies must disclose on these points even when the disclosure is negative.

Sample size	We aim to use parental SNV to determine and phase the two offspring haplotype genome, thus the sample size for genome sequencing is three. Bioinformatic analyses were performed with all available data.
Data exclusions	Sex chromosomes are excluded in genetic variation analysis.<br>In PCR validation, we excluded SNPs located in repeat elements.<br>Variations in chimeric regions were excluded. Various filters were applied at the potential Mendelian violation to reduce false-positive calls, especially at chimerism sites. The first filter was on the site and applied as follows: QD < 2.0, FS > 20.0, MQ < 40.0, MQRankSum < -2.0, MQRankSum > 4.0, ReadPosRankSum < -3.0, ReadPosRankSum > 3.0, SOR > 3.0. The second set of filters were applied to each individual:<br>- a depth filter DP < 0.5 × individual average depth and DP > 2 × individual average (average depth offspring: 40.5X, father: 72.6X, and mother: 76.9X). This filter would remove any high coverage caused by mapping problems and low coverage sites that are more sensitive to false-positive calls.<br>- a genotype quality filter GQ < 99 for at least one individual. This filter was set particularly high (generally GQ < 40 to 60 in other de novo studies) to avoid a maximum of chimerism sites in the father, as those sites tend to have a lower genotype quality due to the presence of multiple alleles.<br>- an alternative allele filter AD > 0 allowed in the homozygous parents. Again, this filter was set stringent with no alternative allele allowed in any parents as most of the chimerism sites would present at least a few alternative alleles in the variant calling files.<br>- an allelic balance filter AB < 0.3 and AB > 0.7 on the reads supporting the alternative allele in the heterozygous offspring. This filter would remove any potential sequencing errors in the offspring or chimerism cells as those should present a lower allelic balance (~10-20 %) than the real de novo mutations (~50 %).<br>In positive selection gene analysis, to minimize effects of alignment, we filtered genes based on the condition of its positively selected sites following these criterions, 1) sites with gap number more than 2 were excluded; 2) sites with nonsynonymous substitutions larger than 2 were excluded; and 3) more complicated cases found manual checks. If one gene had no confident site, the gene would be removed.
Replication	Experiments performed in this study aim to validate the variation between the two alleles of the offspring, thus the experiments were performed based on the offspring DNA sample and replication is not applied in this study.
Randomization	Randomization for genome and transcriptome sequencing is not applied in this study. For SNV and indel PCR validation, variation sites were randomly selected by Linux command "sort -R".
Blinding	Blinding was not necessary for genome and transcriptome sequencing, as well as genetic variation PCR validation. The study aim to study the genetic difference inherent from parental genome, so only the F1 individual DNA sample is used for PCR validation.

Reporting for specific materials, systems and methods

We require information from authors about some types of materials, experimental systems and methods used in many studies. Here, indicate whether each material, system or method listed is relevant to your study. If you are not sure if a list item applies to your research, read the appropriate section before selecting a response.

Materials & experimental systems

n/a	Involved in the study
☒	☐ Antibodies
☒	☐ Eukaryotic cell lines
☒	☐ Palaeontology and archaeology
☐	☒ Animals and other organisms
☒	☐ Human research participants
☒	☐ Clinical data
☒	☐ Dual use research of concern

Methods

n/a	Involved in the study
☒	☐ ChIP-seq
☒	☐ Flow cytometry
☒	☐ MRI-based neuroimaging

Animals and other organisms

Policy information about studies involving animals; ARRIVE guidelines recommended for reporting animal research

Laboratory animals	Species: Callithrix jacchus. No unique strain. Male and female animals used. Ages: mCalJac1 (M) = 3 months, mCalJac2 (M) = 3 years, mCalJac3 (F) = 3 years, mCalJac4 (M) = 1.5 years.
Wild animals	Study did not involve wild animals.
Field-collected samples	Study did not involve field-collected samples.
Ethics oversight	USDA, AAALAC, and The Rockefeller University IACUC

Note that full information on the approval of the study protocol must also be provided in the manuscript.
